# Characterization of T cell receptors reactive to HCRT_NH2_, pHA_273-287_, and NP_17-31_ in control and narcolepsy patients

**DOI:** 10.1073/pnas.2205797119

**Published:** 2022-08-01

**Authors:** Guo Luo, Jing Zhang, Ling Lin, Emmanuel Jean-Marie Mignot

**Affiliations:** ^a^Center for Sleep Sciences and Medicine, Stanford University School of Medicine, Palo Alto, CA 94304

**Keywords:** narcolepsy, DQ0602, hypocretin, tetramer, TCR

## Abstract

Narcolepsy type 1 (NT1), a disorder caused by hypocretin/orexin (HCRT) cell loss, is associated with human leukocyte antigen (HLA)-DQ0602 (98%) and T cell receptor (TCR) polymorphisms. Increased CD4^+^ T cell reactivity to HCRT, especially DQ0602-presented amidated C-terminal HCRT (HCRT_NH2_), has been reported, and homology with pHA_273–287_ flu antigens from pandemic 2009 H1N1, an established trigger of the disease, suggests molecular mimicry. In this work, we extended DQ0602 tetramer and dextramer data to 77 cases and 44 controls, replicating our prior finding and testing 709 TCRs in Jurkat 76 T cells for functional activation. We found that fewer TCRs isolated with HCRT_NH2_ (∼11%) versus pHA_273–287_ or NP_17–31_ antigens (∼50%) were activated by their ligand. Single-cell characterization did not reveal phenotype differences in influenza versus HCRT_NH2_-reactive T cells, and analysis of TCR CDR3αβ sequences showed TCR clustering by responses to antigens but no cross-peptide class reactivity. Our results do not support the existence of molecular mimicry between HCRT and pHA_273–287_ or NP_17–31_.

## Significance

Amidated hypocretin peptides (HCRT_NH2_) are autoantigens in narcolepsy type 1, an autoimmune disorder targeting HCRT neurons. The autoimmune process can be initiated by exposure to the influenza A flu, and a particular piece of the hemagglutinin (HA) of the pandemic 2009 H1N1 strain, as well as a piece of the nucleoprotein segment, were identified as particularly reactive in narcolepsy patients. One hypothesis has been that T cells reactive to these flu segments can also react to HCRT, starting the autoimmune process through molecular mimicry. Although we replicated the finding of a higher number of T cells reactive to HA and HCRT, we could not find evidence for cross-reactivity. More work is needed to explain how the flu can trigger narcolepsy.

Narcolepsy type 1 (NT1) is caused by a loss of hypocretin/orexin (HCRT) neurons in the mediolateral hypothalamus (1–3), with recent data suggesting reversion of the human and animal phenotype with orexin agonists. The disease is strongly associated with human leukocyte antigen (HLA) DQB1*06:02/DQA1*01:02 (98% vs. 25%) (DQ0602) and displays weaker genetic associations with other immune loci, thus suggesting autoimmunity (4–9), although not meeting all criteria for being classified as an autoimmune disease (10). Like other autoimmune diseases, NT1 presents with increased comorbidity with other autoimmune conditions and asthma (11–13).

Onset of NT1 is often abrupt and seasonal, and association with both *Streptococcus pyogenes* (14, 15) and influenza A infections (16) suggests that it may be triggered by winter infections. Most strikingly, prevalence of NT1 increased several folds in mainland China and Taiwan following the 2009 to 2010 “swine flu” H1N1 influenza pandemic (pH1N1) (4, 17, 18), although association with the pandemic is less clear in other countries (19). Vaccination with the pH1N1 vaccine Pandemrix has also been associated with an elevated relative risk for developing narcolepsy of 5- to 14-fold in children and adolescents and 2- to 7-fold in adults (18, 20–22). As Pandemrix is an AS03-adjuvanted vaccine containing the artificially produced reassortant strain X-179A, a mix of *A/Puerto Rico/8/1934* (PR8), an old H1N1 strain derived from pre-2009 seasonal H1N1, and the key H1N1 2009 surface proteins hemagglutinin (HA) and neuraminidase (NA) (23), flu proteins are likely critically involved in triggering NT1. Evidence showing that HLA and T cell receptor (TCR) genetic associations are universal (9, 24–27) is also consistent with a flu trigger, as influenza A infections occur on a global basis (28). Importantly, however, even with Pandemrix vaccination in Europe, only ∼1 in 16,000 vaccinated children developed NT1, thus demanding the consideration of additional factors to fully explain the initiation of NT1 (29).

Unlike in other autoimmune diseases, autoantibodies against HCRT cell proteins, HCRT itself (30–32), or other targets such as TRIB2 (33, 34) or HCRT receptor 2 (35–38) have not been consistently found. This has led to the suggestion that HCRT cell loss may be primarily T cell mediated, with limited or no involvement of autoantibodies. Consistent with this hypothesis, mounting evidence suggests involvement of CD4^+^ T cell reactivity to HCRT in NT1 (39–41), notably toward amidated fragments of the secreted, mature peptide (HCRT_54–66-NH2_ and HCRT_86–97-NH2_, homologous peptides collectively denoted as HCRT_NH2_) (42), as critical factors in the development of the disease. Furthermore, CD8^+^ mediation of HCRT cell death has also been shown to cause NT1 in an animal model (43) and Pedersen and colleagues (44) recently highlighted the presence of CD8^+^ T cell responses against intracellular proteins contained in HCRT neurons in narcolepsy patients. Of additional interest is the observation that the TCR polymorphisms associated with NT1 are quantitative trait loci for TRAJ24 (decreasing), TRAJ28, and TRBV4-2 (increasing) usage in peripheral T cells in both controls and patients (29). A significant L to F coding polymorphism located within the antigen-binding complementarity-determining region (CDR) 3 loop of TRAJ24 expressing TCRs is also associated with NT1. Altogether, this suggests that T cell responses involving TRAJ24- or TRAJ28- and TRBV4-2–bearing TCRs may be bottleneck responses in a causative autoimmune T cell response, leading to HCRT cell death (4, 14, 17–19, 45).

Based on the evidence provided above, our group hypothesized that a CD4^+^ T cell–mediated response directed against specific flu epitopes could lead to molecular mimicry with HCRT itself, potentially HCRT_NH2_, subsequently recruiting CD8^+^ cytotoxic T cells and leading to HCRT cell death. To test this hypothesis, we screened 135 DQ0602 tetramers binding peptides originating from Pandemrix, wild-type 2009 H1N1, and two autoantigens (HCRT and RFX4) for the presence of antigen-restricted CD4^+^ T cells (42). After this systematic survey, it was established that CD4^+^ T cell populations recognizing influenza pHA_273–287_ (pH1N1 specific) and PR8 (H1N1 pre-2009 and H2N2)-restricted NP_17–31_ epitopes were increased in NT1 versus DQ0602 controls. Supporting this finding, this difference was also present in post-Pandemrix cases versus controls and was stronger in recent onset cases (42). Additionally, studies of single cells recognizing these peptides revealed that TCR clones carrying TRBV4-2 and TRAJ24 were retrieved from both HCRT_NH2_ and pHA_273–287_ tetramers (42), suggesting involvement of these clones in molecular mimicry and disease pathophysiology. Similarly, Jiang et al. (39) isolated TRAJ24-positive cells recognizing DQ0602 bound to HCRT_87–100_ tetramer, many of which expressed perforin and granzyme-B, suggesting a terminally differentiated effector T cell (T_EMRA_) phenotype. In one case, a TRAJ24 clone isolated from a narcoleptic patient showed elevated TCR reactivity toward HCRT_87–97-NH2_ when transfected in Jurkat 76 (J76) cells, thus implying a role for TRAJ24 reactivity toward DQ0602-HCRT in narcolepsy autoimmunity (39).

Here, we extend prior work from our group by doubling the number of patients and controls and increasing the representation of TRAJ24F narcolepsy susceptibility–associated alleles in these subjects. Results validated an increased frequency of pHA_273–287_ and HCRT_54–66-NH2_ tetramer-positive CD4^+^ T cells in NT1, while also testing isolated T cell clones for potential activation by their cognate ligands when expressed in J76 cells. Importantly, we also analyzed TCR CDR3αβ sequences in this larger dataset and conducted expression profiling of the corresponding T cells, providing insights into T cell characteristics in narcolepsy.

## Results

### CD4^+^ T Cell Recognizing pHA_273–287_ and HCRT_54–66-NH2_ in NT1.

**Fig. 1.**
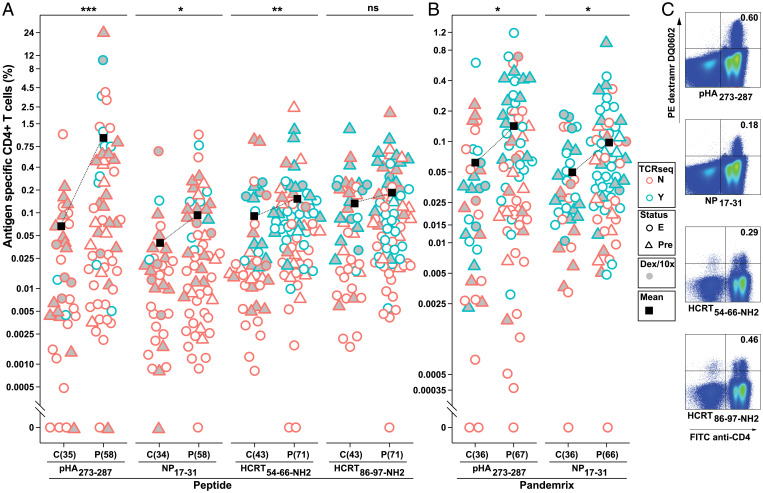
Antigen-restricted CD4^+^ T cells detected with DQ0602 tetramer and dCODE dextramer. (*A* and *B*) PBMCs of 77 NT1 cases and 44 healthy controls [of these, 35 NT1 cases and 22 healthy controls were previously reported in Luo et al. (42)] were cultured individually with the cognate peptides (*A*) or Pandemrix (*B*) and then stained with tetramer DQ0602 of pHA_273–287_, NP_17–31_, HCRT_54–66-NH2_, and HCRT_86–97-NH2_. Subjects previously reported (42) are plotted as triangles and new data as circles. Frequency was calculated in live CD3^+^ T cells. Each triangle or circle represents one subject. If multiple cultures or FACS recordings for one subject occurred, the mean of frequency was used. Subjects carried forward for single-cell sorting and TCR sequencing (TCRseq) are shown in blue and all other individuals in red. PBMCs cultured individually and pooled with the same cognate peptide for dCODE dextramer (Dex/10x) DQ0602 staining and 10x genomics sequencing are shown in gray. Mean frequency is shown in black square. (*C*) FACS plots of live CD3^+^ T cells isolated with dextramer were divided in a quadruple gate. (For complete FACS plots, refer to *SI Appendix*, Fig. S1). N, not sorted. Y, sorted and sequenced in 96-well plates. E, extended; Pre, previously reported in Luo et al. (42); C, control; P, patient; PE, phycoerythrin; FITC, fluorescein isothiocyanate. *P < 0.05; **P < 0.01; ***P < 0.001; ns, not significant.

Ten-day peripheral blood mononuclear cell (PBMC) cultures using pHA_273–287_, NP_17–31_, HCRT_54–66-NH2_ and HCRT_86–97-NH2_ (HCRT_NH2_), and Pandemrix were stained with the corresponding tetramers in 42 NT1 cases and 22 healthy DQ0602 controls (Table 1 and Dataset S2). Together with previously reported subjects (42), a total of 77 NT1 cases and 44 healthy controls have now been studied with now comparable counts of TRAJ24 F/L allele in each group (Table 1 and Dataset S2). Differential reactivity to pHA_273–287_ and HCRT_54–66-NH2_ was reproduced in the second dataset, resulting in increased significance in the combined study (Fig. 1 *A* and *B* and Table 1). Interestingly, reactivity to these antigens was not dependent on age, sex, and past Pandemrix vaccination status (Table 1).

**Table 1.** Frequency of antigen-restricted CD4^+^ T cells in DQ0602 tetramer

	Extended				Previously reported in Luo et al. (42)				Total			
	Case	Healthy control	*P* value	Adj. P*	Case	Healthy control	*P* value	Adj. P*	Case	Healthy control	*P* value	Adj. P*
No.	42	22			35	22			77	44		
Age, y, median [range]	19.9 [7.5–89.8]	30.7 [5.7–64.6]	0.5269		17.9 [6.2–52.3]	19.85 [10.2–56.4]	0.9796		18.7 [6.2–89.8]	20.6 [5.7–64.6]	0.211	
Female, No. (%)	26 (61.9)	11 (50)	0.307		18 (51.4)	9 (40.9)	0.5252		44 (57.1)	20 (45.5)	0.3534	
EO, No. (%)	5 (11.9)	n.a.			10 (28.6)	n.a.			15 (19.5)	n.a.		
Px, No. (%)	4 (9.5)	4 (18.2)	0.0098		16 (45.7)	11 (50)	0.2349		20 (26)	15 (34.1)	0.3198	
AJ24 FF, No. (%)	24 (57.1)	9 (40.9)	0.1261^†^		1 (2.9)	1 (4.5)	0.8919^†^		25 (32.5)	10 (22.7)	0.4274^†^	
AJ24 FL, No. (%)	6 (14.3)	8 (36.4)			21 (60)	12 (54.5)			27 (35.1)	20 (45.5)		
AJ24 LL, No. (%)	12 (28.6)	5 (22.7)			13 (37.1)	9 (40.9)			25 (32.5)	14 (31.8)		
pHA_273–287_^‡^	0.027 [0–10.4] (35)	0.0072 [0–1.09] (21)	0.0087	0.0234	0.12 [0–23.8] (23)	0.037 [0–0.22] (14)	0.0173	0.0059	0.0725 [0–23.8] (58)	0.0074 [0–1.09] (35)	6.00E-04	7.87E-04
NP_17–31_^‡^	0.017 [0–1.08] (35)	0.013 [9E-04–0.65] (21)	0.2901	0.377	0.049 [0.0021–0.3] (23)	0.017 [0–0.095] (13)	0.0412	0.0216	0.0265 [0–1.08] (58)	0.014 [0–0.65] (34)	0.0372	0.0294
HCRT_54–66-NH2_^‡^	0.0568 [0–0.26] (42)	0.0205 [8E-04–0.26] (22)	0.1554	0.0204	0.0957 [0.0073–2.43] (29)	0.019 [0.005–0.9227] (21)	0.0013	0.0065	0.065 [0–2.43] (71)	0.02 [8E-04–0.9227] (43)	0.0017	7.20E-04
HCRT_86–97-NH2_^‡^	0.036 [0–0.445] (42)	0.0195 [0.0017–0.27] (22)	0.3294	0.2645	0.188 [0.0185–2.0745] (29)	0.113 [0.0058–1.272] (21)	0.0561	0.0388	0.0736 [0–2.0745] (71)	0.055 [0.0017–1.272] (43)	0.2195	0.0916
Px-pHA_273–287_^‡^	0.0525 [0–1.1833] (43)	0.0225 [0–0.6] (22)	0.1255	0.3275	0.085 [0.0017–0.49] (24)	0.028 [0.0023–0.23] (14)	0.0474	0.0162	0.059 [0–1.1833] (67)	0.024 [0–0.6] (36)	0.0148	0.0315
Px-NP_17–31_^‡^	0.045 [0–0.44] (43)	0.0395 [0–0.185] (22)	0.4624	0.3413	0.065 [0.005–0.94] (23)	0.0225 [0.0036–0.089] (14)	0.0319	0.0096	0.0513 [0–0.94] (66)	0.0278 [0–0.185] (36)	0.0402	0.0129

EO, early onset; n.a., not applicable; Px, Pandemrix.

^*^Adjusted *P* value by age at blood draw, gender, diagnosis, and Pandemrix vaccination. Refer to Datasets S2 and S10 for details.

^†^χ^2^ test of TRAJ24 alleles and diagnosis.

^‡^Frequency of antigen-specific CD4^+^ T cells is shown as % [range] No. of tested individuals.

Another aim was to characterize the TCRs of tetramer/dextramer-isolated T cells and determine cross-reactivity within and between peptides of interest. To do so, in addition to tetramers, we also used DQ0602 DNA–bar-coded dextramers (dCODE dextramers) in pooled PMBCs, cultured individually with pHA_273–287_, NP_17–31_, HCRT_54–66-NH2_, HCRT_86–97-NH2_, and other peptides (Fig. 1*C* and *SI Appendix*, Fig. S1 and Dataset S1). Although both techniques successfully retrieved antigen-restricted cells, recovered TCR clones were less enriched in dCODE dextramer experiments in comparison to DQ0602 tetramer studies (8.3% vs. 61.6% in total) (Dataset S5). Altogether, 542 tetramer-isolated and 167 dextramer-isolated TCR clones, including 52 found in both tetramer and dextramer experiments, were selected for functional testing in J76 cells that do not express any TCR. These TCRs include 252 previously isolated TCRs reported in Luo et al. (42).

### Testing of TRAJ24-, TRAJ28-, and TRBV4-2–Positive Clones for Activation.

Continuing on from our prior study (42) and considering intervening literature (39), we first tested TRAJ24-, TRAJ28-, and TRBV4-2–positive clones that were suggested to be involved in the pathophysiology of NT1. This included the TRAJ24-positive, HCRT_87–97-NH2_–activated clone TCRbm (TRAV6-CALTTDSWGKLQF-TRAJ24/TRBV29-1-CSVEGDRGRSETQYF-TRBJ2-5) reported by Jiang and colleagues (39) and prior TRAJ24 and TRBV4-2 sequences isolated in Luo et al. (42). Disappointingly, we could not confirm activation of TCRbm by HCRT_NH2_ (Dataset S6). Similarly, we found no activation of 53 clones containing a prior TRAJ24 sequence that was enriched across pHA_273–287_, NP_17–31_, HCRT_54–66-NH2_, and HCRT_86–97-NH2_ antigens in our previous work (CDR3α, TRAV2-CAVETDSWGKLQF-TRAJ24) (Dataset S6 and *SI Appendix*, Fig. S3) (42).

Additional functional testing was carried forward with other TCRs either because they were highly enriched, bearing TRAJ24, TRBV4-2, or TRAJ28 or having CDR3α/β sequence similarity (188 clones), or because they were found at least three times (448 clones) (Dataset S6). These were individually expressed in J76 cells, and the resulting cell lines were tested for immune signaling by coculturing J76-TCR, artificial DQ0602-positive antigen-presenting cells (APCs) (RM3-DQ0602), and each corresponding antigen.

Out of 709 TCRs screened (53 TRAJ24, 21 TRBV4-2, including 1 TCR bearing both TRAJ24 and TRBV4-2, plus 188 and 448 above), 103 (tetramer, 67; dextramer, 36; including 13 shared), 89 (tetramer, 77; dextramer, 12; including 16 shared), and 37 (tetramer, 31; dextramer, 6; including 2 shared) clones were activated by pHA_273–287_, NP_17–31_, and HCRT_NH2_, respectively (Dataset S6).

Distinct clonotypes in cases and controls were detected using tetramers and dextramers for restricted antigens of pHA_273–287_ (tetramer, 44 patients only [P], 20 controls [C], 3 controls and patients [C/P]; dextramer, 21 P, 10 C, 5 C/P), NP_17–31_ (tetramer, 38 P, 32 C, 7 C/P; dextramer, 9 P, 3 C, 0 C/P), and HCRT_NH2_ (tetramer, 20 P, 8 C, 3 C/P; dextramer, 4 P, 1 C, 1 C/P) (Table 2). Of note, two TCR clones containing TRBV4-2-CASSQETQGRNYGYTF-TRBJ1-2 (TCR43 and 275) pairing only with TRAV13-1-CAASDNDMRF/CAANNNDMRF-TRAJ43 were activated by pH_A273-287_ but were negative with 19 other CDR3α (Dataset S6 and *SI Appendix*, Fig. S3). While 41 TCRs out of 542 in tetramer experiments and 95 out of 167 in dextramer experiments were recognized by two or three peptides encompassing pHA_273–287_, NP_17–31_, and HCRT_NH2_, none was ever activated across peptide classes (Datasets S6 and S7).

**Table 2.** TCRs retrieved and activated using DQ0602 tetramers and dextramers

Antigen	Dx	Total				Tetramer				Dextramer				Shared in tetramer and dextramer			
		No.	apHA_273–287_, No. (%)	aNP_17–31_, No. (%)	aHCRT_NH2_, No. (%)	No.	apHA_273–287_, No. (%)	aNP_17–31_, No. (%)	aHCRT_NH2_, No. (%)	No.	apHA_273–287_, No. (%)	aNP_17–31_, No. (%)	aHCRT_NH2_, No. (%)	No.	apHA_273–287_, No. (%)	aNP_17–31_, No. (%)	aHCRT_NH2_, No. (%)
All	T	709	103 (14)	89 (12)	37 (5.2)	542	67 (12)	77 (14)	31 (5.7)	167	36 (22)	12 (7.2)	6 (3.6)	52	13 (25)	16 (31)	2 (3.8)
	P	475	65 (13.7)	47 (9.9)	24 (5.1)	367	44 (12)	38 (10)	20 (5.4)	108	21 (19)	9 (8.3)	4 (3.7)	33	10 (30)	9 (27)	
	C	187	30 (16)	35 (19)	9 (4.8)	149	20 (13)	32 (22)	8 (5.4)	38	10 (26)	3 (7.9)	1 (2.6)	4		3 (75)	
	C|P	47	8 (17)	7 (15)	4 (8.5)	26	3 (12)	7 (27)	3 (12)	21	5 (24)		1 (4.8)	15	3 (20)	4 (27)	2 (13)
pHA_273–287_	T	124	62 (50)		1 (0.8)	110	61 (56)			14	1 (7.1)		1 (7.1)	4	3 (75)		
	P	87	42 (48)		1 (1.1)	81	42 (52)			6			1 (17)	4	3 (75)		
	C	36	20 (56)			29	19 (66)			7	1 (14)						
	C|P	1								1							
NP_17–31_	T	137	1 (0.7)	61 (45)		133		61 (46)		4	1 (25)						
	P	81		31 (38)		78		31 (40)		3							
	C	55	1 (1.8)	29 (53)		54		29 (54)		1	1 (100)						
	C|P	1		1 (100)		1		1 (100)									
HCRT_NH2_	T	293	3 (1)	1 (0.3)	29 (9.9)	253			27 (11)	40	3 (7.5)	1 (2.5)	2 (5)	8			2 (25)
	P	212	2 (0.9)	1 (0.5)	18 (8.5)	185			17 (9.2)	27	2 (7.4)	1 (3.7)	1 (3.7)	4			
	C	68	1 (1.5)		8 (12)	61			8 (13)	7	1 (14)						
	C|P	13			3 (23)	7			2 (29)	6			1 (17)	4			2 (50)

All are TCRs retrieved from any peptide combination; pHA_273–287_, NP_17–31_, and HCRT_NH2_ are TCRs retrieved from a single peptide; No. is the count of TCR clones tested; T is the total number of subjects; P are patients only; C are controls only; P|C include both patients and controls; apHA_273–287_ are clones activated by pHA_273–287_; aNP_17–31_ are clones activated by NP_17–31_; aHCRT_NH2_ are clones activated by HCRT_NH2_; Dx is diagnosis. TCRs shared in tetramer and dextramer are included in both tetramer and dextramer counts.

### Probability of Activation of All Isolated TCRs by Their Cognate Ligands.

TCRs retrieved by tetramer experiments (that used single-peptide tetramers) were always found to be activated by their corresponding cognate peptide. In addition, tetramers retrieved using viral peptide had a much higher probability of activation by their cognate ligand than those retrieved with HCRT_NH2_ tetramers (pHA_273–287_, 55.5%; NP_17–31_, 45.9%; HCRT_NH2_, 10.7%) (Table 2). For dextramer-isolated clones (that used a single peptide), the specificity was not as good. These were less frequently activated by their cognate ligand (pHA_273–287_, 7.1%; NP_17–31_, 0; HCRT_NH2_, 5%) (Table 2) and could occasionally be activated by one of our other tested peptides (Table 2). For instance, 1 of 14 TCRs retrieved with pHA_273–287_ was activated by HCRT_NH2_, and one out of four TCRs retrieved with NP_17–31_ was activated by pHA_273–287_. Further, 3 and 1 of 40 TCRs retrieved with HCRT_NH2_ were activated by pHA_273–287_ and NP_17–31_, respectively (Table 2). This suggests that dextramer specificity was lower.

Next, we investigated TCR sequences that have been retrieved by more than one tetramer/dextramer ligand. Here again, dextramer-retrieved TCR clones were more frequently found across peptide ligands, with 109 TCRs out of 167 cross peptide TCR sequences found in dextramer experiments compared with 46 out of 542 in tetramer experiments (65.3% vs. 8.5%; see Dataset S7 for further details). We hypothesized that the lack of specificity was due to bulk sorting and pooled library sequencing of CD4^+^ T cells. For this reason, we considered a given TCR retrieved by dextramer as “peptide restricted” only when activation in Jurkat cells had been verified.

Unsurprisingly, the probability for any isolated clone to be activated in J76 cells increased with clonal abundance (*SI Appendix*, Fig. S5). Interestingly, however, the relationship was different for viral antigens (pHA_273–287_/NP_17–31_) versus autoantigen HCRT_NH2_ (*SI Appendix*, Fig. S5); for example, activation increased to 70% for pHA_273–287_/NP_17–31_ TCR clones present more than five times, whereas for HCRT_NH2_, clones needed to be present over 35 times to reach that probability of activation. Similarly, dextramer TCR activation probability increased up to 20% and 7.5% from 1 to 10 TCR counts for pHA_273–287_ and HCRT_NH2_, respectively (*SI Appendix*, Fig. S5). Altogether, these findings demonstrate cross-reactivity within peptide classes (e.g., between pHA_273–287_ and pHA_329–343_) but not across peptide classes (e.g., between HA and NP segments of Pandemrix or between virus and autoantigens) and higher activation probability for viral cognate antigens rather than autoantigens, thus not supporting our hypothesis of molecular mimicry between pHA_273–287_ and HCRT_NH2_ in the pathophysiology of NT1.

### TCR Activation across Antigens (Cross-Reactivity).

**Fig. 2.**
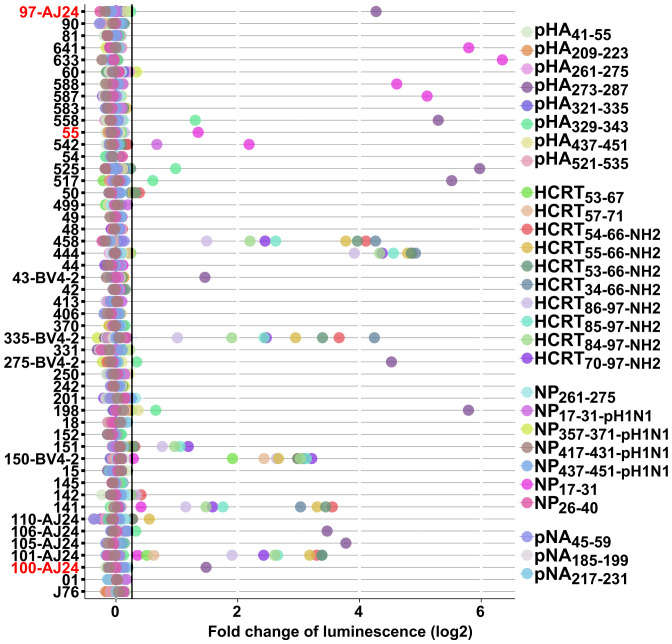
Specificity of TCR responses to flu and HCRT antigens. Forty-eight TCRs were randomly selected and cocultured with artificial APCs and antigens originating from Pandemrix and HCRT for 8 h in triplicate (*n* = 3). Luciferase activity was measured. Threshold of fold change of luminescence (threshold = 1.2) is shown. J76 without transfection was used as a control. TCR clones retrieved by both tetramer and dextramer DQ0602 are highlighted in red. See Datasets S1 and S6 for peptides and TCRs, respectively.

To investigate TCR activation specificity by antigens, we randomly selected 48 clones cocultured with previously reported 28 binders originating from different Pandemrix and virus segments (HA, 8; NP, 7; NA, 3) and HCRT (10, 42), including pHA_273–287_, NP_17–31_, HCRT_54–66-NH2_, and HCRT_86–97-NH2_ (Dataset S1). These latter HCRT peptides contain C-terminal binding core GNHAAGILTL/M but differ in length and amidation (preproHCRT14/15 are C-terminal nonamidated; Dataset S1). We also confirmed specific TCR reactions to pHA_273–287_, NP_17–31_, HCRT_54–66-NH2_, and HCRT_86–97-NH2_ (Fig. 2 and Dataset S6). Importantly, TCRs activated by HCRT_54–66-NH2_ and/or HCRT_86–97-NH2_ were also activated by other HCRT_NH2_ peptides of different length, thus further validating their specific activation by HCRT_NH2_ (Fig. 2). We noticed that three TCR clones, TCR101 (TRAV20-CAVQARSWGKLQF-TRAJ24/TRBV2-CASTGSYNSPLHF-TRBJ1-6), TCR141 (TRAV29/DV5-CAASDTGTASKLTF-TRAJ44/TRBV9-CASSVVGSYGYTF-TRBJ1-2), and TCR150 (TRAV12-2-CAVVHNAGNMLTF-TRAJ39/TRBV4-2-CASSQGPDSRETQYF-TRBJ2-5) were activated by both NP_17–31_ and HCRT_NH2_ with one replication, though the signal of NP_17–31_ decreased in the second experiment (Fig. 2 and Dataset S6). Unfortunately, however, cross-reactivity with NP_17–31_ could not be confirmed by multiple subsequent experiments. Surprisingly, 6 out of 10 TCR clones (TCR106, 198, 275, 517, 525, and 558) activated by pHA_273–287_ (HA69, 273-AMERNAGSGIIISDT-287) were also activated by pHA_329–343_ (HA83, 329-LRLATGLRNIPSIQS-343), suggesting cross-reactivity between these antigens (Fig. 2). Interestingly, despite high sequence similarity (Dataset S1), only one out of six clones (TCR542) activated by NP_17–31_ (NP136, 17-GERQNATEIRASVGK-31) was also activated by NP_17–31-pH1N1_ (NP37, 17-GERQDATEIRASVGR-31) (Fig. 2) (NP136 originates from PR8 and NP37 from *A/California/07/2009*) (42). Taken together, these findings indicate that cross-reactivity between HCRT, pHA_273–287_, or NP_17–31_ must be nonexistent or rare as it was not observed in 709 clones.

### TCR Segment Usage in TCR Sequences Retrieved by Tetramer/Dextramer Experiments.

In a next step, we looked at preferential TCR segment usage in distinct TCR reactions. Extending on preliminary data, we found preferential usage of TRBV19 and TRAV12-2 for pHA_273–287_ and NP_17–31_, respectively, while HCRT_NH2_ weakly preferred to use TRAV12-3 and TRBV2 (*SI Appendix*, Fig. S6). TRAJ24- and TRBV4-2–bearing TCRs were activated by all three antigens (*SI Appendix*, Fig. S3), although TRBV4-2 and TRAJ24 were preferentially used by TCRs recognizing NP_17–31_ and pHA_273–287_, respectively. Notably, only two case-associated TRAJ28-bearing clones were found, one reactive to pHA_273–287_ and the other to HCRT_NH2_ (*SI Appendix*, Fig. S6). Of clones bearing TRAJ24, both F and L alleles were present (*SI Appendix*, Fig. S6).

### Phylogenetic Tree of Isolated TCR Clones.

**Fig. 3.**
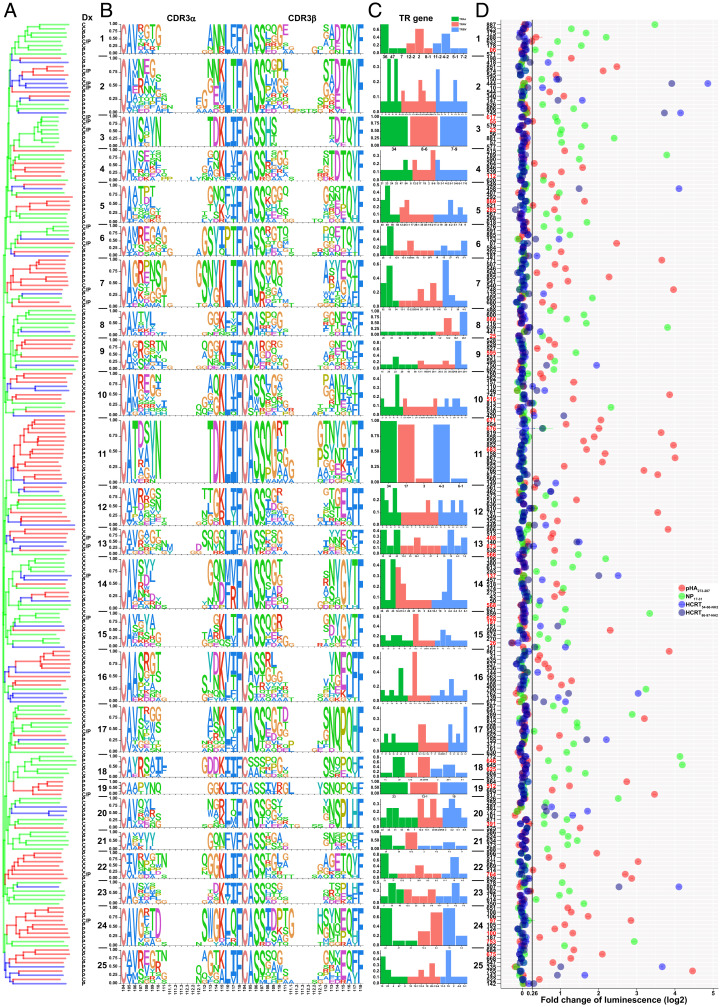
TCR activation by pHA_273–287_, NP_17–31_, HCRT_54–66-NH2_, and HCRT_86-97-NH2_. Each paired TCR was transfected to engineered J76 cells (not expressing any TCR) featuring NFAT-luciferase and sorted for positive population. They were cocultured with artificial APCs at the presence of pHA_273–287_, NP_17–31_, HCRT_54–66-NH2_, HCRT_86–97-NH2_, and RFX4-43 for 8 h in triplicate (*n* = 3). TCR was considered as activated by a peptide when ≥1.2-fold change of luminescence compared to RFX4-43 with *P* < 0.05 was observed. (*A*) A phylogenetic tree was constructed using paired CDR3αβ sequences with BLSOUM62 matrix, and 25 groups were designated based on sequence similarity. Branch color corresponds to activation peptide: red, pHA_273–287_; green, NP_17–31_; blue, HCRT_54–66-NH2_ and/or HCRT_86–97-NH2_. Each branch is denoted by the diagnosis (Dx) of the subjects from whom the TCRs were recovered. C, control; P, patient; C/P in both controls and patients. (*B*) Sequence logo for each group CDR3αβ was generated using the unique numbering and gaps of TCR V-region. Amino acid height is proportional to prevalence at that position. (*C*) Usage frequency of TRAV, TRAJ, and TRBV. *x*-axis: TR genes of each category of TRAJ (green), TRAV (red), and TRBV(blue). *y*-axis: Frequency of each TR gene in corresponding category. (*D*) Fold change of luminescence for activated TCRs by each peptide. TCR clones retrieved by both DQ0602 tetramers and dextramers are highlighted in red. Group (1 to 25) and TCR numbers (01 to 890) are labeled at *y*-axis. A vertical line of threshold fold change is shown. For full set of TCRs, refer to Dataset S6.

To investigate TCR similarity across activation groups, several approaches were taken: construction of phylogenetic trees, substitution analysis, and grouping of lymphocyte interactions by paratope hotspots (GLIPH) motif identification. Regarding the first approach, a phylogenetic tree containing all activated CDR3αβ TCR sequences was constructed, defining 25 groups (Fig. 3). TCRs activated by pHA_273–287_ or NP_17–31_ were primarily clustered into clades, five of which included at least five TCRs in each. In contrast, HCRT_NH2_ CDR3αβ-reactive clones were distributed sporadically across many clades and often comprised single clones with minimal homology between them (Fig. 3). These HCRT_NH2_ CDR3αβ-reactive clones were also present across both primary pHA_273–287_ and NP_17–31_ clades (Fig. 3). A major clade of pHA_273–287_ (group 11) included TRAV17/TRAJ34 paired with TRBV4-3 and included one HCRT_NH2_ TCR outgroup. Another pHA_273–287_ clade (group 24) bearing TRAJ24/TRBV19 from both tetramer and dextramer was found in both NT1 cases and controls (Fig. 3). For NP_17–31_–activated TCRs, one clade (group 3) was TRAV8-6/TRAJ34 pairing TRBV7-9/TRBJ2-3 with one and two amino acid differences in CDR3α and CDR3β, respectively. Four distinct TRBV4-2 TCRs were in another clade (group 1) (Fig. 3). Despite a distant relationship to flu TCRs, five minor HCRT_NH2_ clades were found in groups 2, 10, 14, 16, and 20 (Fig. 3). Although TRBV4-2 was widely used by these epitopes, clades containing TRAJ24 were rare and included solely 4, 8, 10, 20, and 24 (Fig. 3). Of note, TRAJ24F to L substitution (TCR727 vs. 101) isolated from tetramer in an NT1 case was also found to be reactive to HCRT_NH2_ in clade 20, a poorly homologous mix of pHA_273–287_ and NP_17–31_ clade (Fig. 3).

**Fig. 4.**
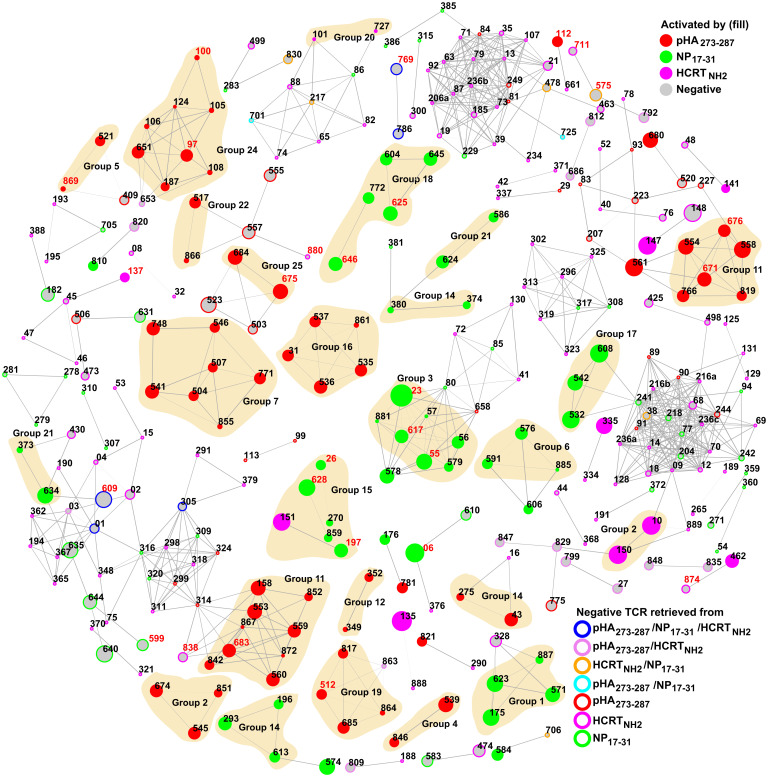
Similarity of activated TCR CDR3 sequences. The network was constructed using hamming distances computed for paired TCR CDR3 amino acid sequences tested in J76 cells (see Dataset S6). Edges (gray lines) connect sequences that differ by up to 10 amino acid substitutions. Edge width indicates the number of substitutions, whereby the thinner the edge, the fewer substitutions detected. Each vertex (node) represents a TCR clone, and the corresponding TCR number is annotated. TCR clones retrieved by both DQ0602 tetramers and dextramers are highlighted in red. Color of vertex encodes activation by the different peptides pHA_273–287_, NP_17–31_, and HCRT_NH2_. Negative clones are shown in gray with different border colors indicating the antigens used to retrieve them. The size of the vertex is scaled by clone count of each TCR. Group members (see Fig. 3) that are connected are highlighted in yellow background.

We next computed hamming distances across all identified 709 TCRs (activated or not, using tetramer or dextramer) containing up to 30 amino acid substitutions. The percentage of TCRs activated by the cognate peptide versus not with a given number of substitutions was calculated and sorted by numeric order of substitutions (Dataset S8). As can be seen in Dataset S8, ∼70% of TCRs remained activated by the same viral cognate ligand when containing up to 10 CDR3α/β substitutions (pHA_273–287_, 76%; NP_17–31_, 69%), after which the chance of nonactivation started to increase (Dataset S8). Insertions and deletions were also tested but resulted in immediate loss of activation. We further built a network using connected TCRs based on the cutoff of 10 CDR3α/β substitutions (Fig. 4). This revealed clear clustering by peptides, with at least two connected TCR clones belonging to 20 out of 25 groups in most cases (except for 8, 9, 10, 13, and 23) (Figs. 3 and 4), validating both approaches of phylogenesis and substitution analyses. As can be seen (Fig. 4), TCRs activated by each virus antigen were clustered, while HCRT_NH2_-reactive TCRs were closer to nonactivated clones (Fig. 4). Of note, TCR150 and TCR10, clones activated by HCRT_NH2_ in group 2, were connected to each other (three substitutions in CDR3α and six in CDR3β; Fig. 4 and Dataset S6). Interestingly, another HCRT_NH2_-reactive TCR151 was connected to TCR197 activated by NP_17–31_ in group 15 (six substitutions in CDR3α and three in CDR3β; Figs. 3 and 4 and Dataset S6). We also found several large clusters of nonactivated clones either isolated or weakly connected to activated TCRs (Fig. 4), further supporting our effective substitution cutoff of 10. Furthermore, nonactivated TCRs retrieved from HCRT_NH2_ peptides constituted most of the nonactivated clusters (Fig. 4), in agreement with our phylogenetic analysis (*SI Appendix*, Fig. S7). Interestingly, however, several connections reflecting homology between HCRT_NH2_-reactive TCRs and nonactivated TCRs retrieved by HCRT_NH2_ were observed (e.g., 141–48, 462–874, 150–829; Fig. 4), suggesting that some nonactivated clones may be sequence specific.

Lastly, GLIPH motifs (46) were generated and compared by activation group (Dataset S9), identifying motifs for pHA_273–287_– and NP_17–31_–activated clusters, but not for HCRT_NH2_ clones due to fewer activated clones identified. As an example, SQG was found in group 11 activated by pHA_273–287_ and in group 15 activated by NP_17–31_ (Fig. 4 and Dataset S9). TGH and SID were detected in group 24 activated by pHA_273–287_. SHT/S and TDSN were found in group 3 and group 17 activated by NP_17–31_, respectively (Fig. 4 and Dataset S9). Overall, these analyses indicate clear distinctions between clusters activated by each peptide and provide little evidence for resemblance across groups of TCRs activated by each peptide (46).

### Conserved CDR3α/β Amino Acid Residues.

**Fig. 5.**
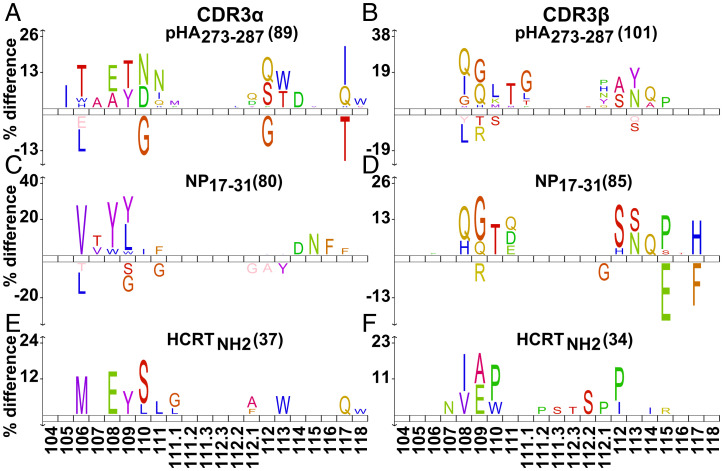
Activation TCR motif of pHA_273–287_, NP_17–31_, and HCRT_NH2_. (*A*–*F*) Unique CDR3α (*A*, *C*, *E*) and CDR3β (*B*, *D*, *F*) activated by each peptide were compared with the public single-cell CD4 TCR reference set ranging from 9 to 21 amino acids in length (CDR3α, *n* = 56,501; CDR3β, *n* = 65,688) (see *Materials and Methods*). Each amino acid was assigned a position using unique numbering of TCR V-region, and gaps were inserted in shorter sequences. Only amino acids with significantly different usage frequency (*P* < 0.05) are shown and visualized using Icelogo. Positively associated amino acids at each relative position are shown above the *x*-axis, and negatively associated amino acids are shown below. Amino acid height is proportional to prevalence at that position. The counts of unique CDR3 sequences are shown in parentheses.

We finally examined preferential positioning of specific amino acids within CDR3s per antigen (pHA_273–287_, NP_17–31_, HCRT_NH2_) using reference numbering (47). These were then compared to two reference sets, a public single-cell CD4^+^ TCR set (CDR3α, *n* = 56,501; CDR3β, *n* = 65,688; see *Materials and Methods*) and our own set of negative, nonactivated TCRs (CDR3α, *n* = 385; CDR3β, *n* = 368) (47). As expected, amino acid usage varied mostly at positions 107 to 110 for CDR3α and at 108 to 111 for CDR3β, while conserved amino acids were largely found in known consensus positions (Fig. 5 and *SI Appendix*, Fig. S8). This analysis revealed clear preferential amino acid usage per ligand in contrast to what was observed for negative (nonactivated) TCRs (Fig. 5 and *SI Appendix*, Fig. S8). For example, CDR3α 108-E is shared across clones activated by pHA_273–287_ and HCRT_NH2_. Further, 108-W is shared by NP_17–31_, while 109-Y is shared across all three antigens. Similarly, CDR3β 109-G/114-Q and 109-E/114-I are frequently found in TCRs activated by pHA_273–287_/NP_17–31_ and HCRT_NH2_, respectively. While CDR3α 114-D and CDR3β 113-N were shared by both virus antigens, CDR3β 112.1-P was shared by pHA_273–287_ and HCRT_NH2_. CDR3α 110 and CDR3β 110 used distinct amino acids across all three antigens (Fig. 5 and *SI Appendix*, Fig. S8). These results indicate unique and shared amino acid usage for pHA_273–287_–, NP_17–31_–, and HCRT_NH2_–activated TCRs, without clear resemblance across groups.

In a final analysis, amino acid usage was computed for CDR3 sequences isolated in NT1 versus control subjects and compared to public CD4^+^ TCR reference (see *Materials and Methods*). Although CDR3α 107-A, 109-T, 110-N/D, 113-T/W, 114-D and CDR3β 111-T, 111.1-G, 113-N, 114-Q were preferentially used in NT1 (*SI Appendix*, Fig. S9 *A*–*D*), the significance of this result is difficult to assess. Lastly, as NT1 is genetically associated with TRAJ24 and TRBV4-2, activated and negative clones using these segments were separately compared to public CD4^+^ TCR reference sequences carrying the same segments (see *Materials and Methods*). Activated TRAJ24 and TRBV4-2 preferentially used CDR3α 107-G/Q, 108-A and CDR3β 109-G, 114-D/S, 115-P (*SI Appendix*, Fig. S9 *E*–*H*), suggesting their roles in immune responses even with fewer activated TRAJ24 clones (12).

### Gene Expression.

#### Tetramer experiments.

**Fig. 6.**
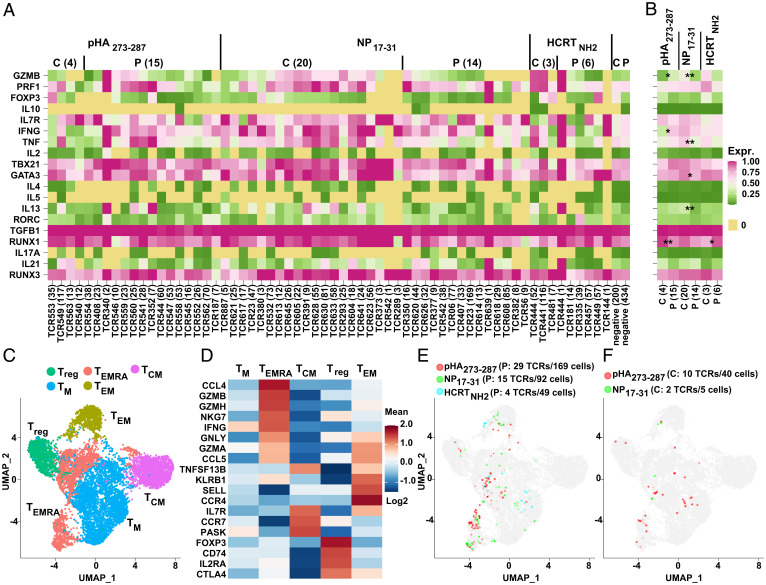
Single-cell gene profiling with TCR. (*A*) Phenotyping of tetramer-sorted single cells of activated TCR clones in NT1 cases and healthy controls. To eliminate multiple-round nested PCR bias while sequencing, each gene expression was assigned to 0 (reads are zero in Dataset S3) or 1 (reads are nonzero in Dataset S3) to indicate the absence or presence of transcripts regardless of actual read counts. Cells with less than five gene markers detected were removed. The mean expression of the remaining narcolepsy or control single cells sharing the same activated TCR clones was calculated and is shown as a heatmap with corresponding TCR number along the *x*-axis. Cells containing nonactivated TCRs were divided into only two groups, depending on whether they were isolated from cases or controls. For cells with alternative TCRα chains, all were tested for reactivity in both α/β combinations, and only the combination found to be activated is reported. Counts of unique TCR clones are shown after diagnosis. (*B*) The mean expression (Expr.) of cells in NT1 cases and controls. Student *t* test was performed between cases and controls for each peptide. (*C*) UMAP of antigen-restricted CD4^+^ T cells of pHA_273–287_, NP_17–31_, and HCRT_NH2_ using DQ0602 dCODE dextramer. Gene expression of filtered single cells (see quality control in *Materials and Methods*) were scaled and clustered using Seurat SCTransform. Five distinct groups of cells were identified. (*D*) Combined well-established gene markers of each cluster (Wilcoxon rank sum test) from the literature. Colors encode the Expr. of each gene. (*E* and *F*) Case (*E*) and control (*F*) cells with activated TCR clones were highlighted with different colors in the UMAP. The counts of total activated TCR clones and cells for each peptide are shown in parentheses. C, control; P, patient; T_EMRA_, effector memory T cell re-expressing CD45RA. *P < 0.05; **P < 0.01; ***P < 0.001.

For tetramer experiments, 31 selected markers were amplified using three rounds of nested PCR in 96-well plates(48). Because nested PCR reactions bias transcript level, read counts (Dataset S3) only represent the presence or absence of transcript and were assigned 1 (nonzero) or 0 (reads, zero) (Dataset S3) in each case. Cells with less than five amplified markers were removed (Dataset S3), leaving 6,435 cells, 2,722 of which had TCRs tested in J76 for activation. Out of 31 markers tested, only 19 were analyzed, including two for T_EMRA_, two for T regulatory cell (Treg), and one for effector memory T cell (T_EM_); the other 12 markers were either not amplified or not available (Fig. 6 and Dataset S3 and *SI Appendix*, Fig. S10). Altogether, 2,088 cells with activated TCR clones (pHA_273–287_: C, 177; P, 441; NP_17–31_: C, 563; P, 563; HCRT_NH2_: C, 175; P, 169) and 634 cells with negative clones (C, 200; P, 434) were clustered according to donor diagnosis and activation antigens (*SI Appendix*, Fig. S10*A*). Surprisingly, T_EMRA_ markers GZMB and/or PRF1 were detected more frequently in control versus NT1 cells (*SI Appendix*, Fig. S10 *B* and *C*), suggesting a possible role of T_EMRA_. Further, activated TCR clones from cases and controls were grouped using the mean expression of each gene (Fig. 6*A*). While GZMB showed differential expression between case and control clones for virus antigens, no significant difference was evident for autoantigen HCRT_NH2_ (Fig. 6*B*) (48).

#### Dextramer experiments.

Expression profiles of CD4^+^ T cells can be better examined in dextramer experiments, as the transcriptome is better interrogated using 10X technology. A total of 9,257 filtered single cells from DQ0602 dextramer (pHA_273–287_, 1,117; NP_17–31_, 469; HCRT_NH2_, 7,671; case, 8,834; control, 423) were studied, undergoing dimensionality reduction, embedding with uniform manifold approximation (UMAP) (49), and unsupervised clustering (see *Materials and Methods*). Using these methods, five distinct groups of DQ0602 dextramer CD4^+^ T cells could be identified, Treg, T_EM_, central memory T cells (T_CM_), T_EMRA_ cells, and memory T cells (T_M_) (Fig. 6 *C* and *D*). Of note, however, only a small portion of T_EMRA_ cells were located close to T_CM_ (Fig. 6 *C* and *D*).

With use of this map, 38 (tetramer, 6; dextramer, 32; total cells, 209), 17 (tetramer, 10; dextramer, 7; total cells, 97), and 4 (all dextramer; total cells, 49) TCR clones activated by pHA_273–287_, NP_17–31_, and HCRT_NH2_, as well as 54 negative TCR clones (total cells, 368), could be positioned. As shown, cells with identical TCR clonotypes had similar profiles and were closely clustered, validating the methodology. Activated clones were mainly located in the T_EMRA_ and T_EM_ compartments, although a few Tregs were also observed. Interestingly, very few activated pHA_173–287_ and NP_17–31_ clones and one HCRT_NH2_ clone (22 cells of TCR750) were found in the T_M_ compartment, together with some negative TCRs. Of note, negative TCRs were evenly scattered, except for T_CM_; only one NP_17–31_–activated and one negative TCR clone were found to derive from this population (Fig. 6 *E* and *F* and *SI Appendix*, Fig. S10*D*). These results were largely congruent with those obtained with tetramer-isolated cells (49).

## Discussion

In this study, we confirmed that NT1 is associated with higher frequencies of DQ0602 antigen–restricted CD4^+^ T cells reactive to pHA_273–287_, NP_17–31_, and HCRT_54–66-NH2_. These results were most striking for pHA_273–287_, a pH1N1-specific antigen, and for HCRT_54–66-NH2_, the HCRT_NH2_ peptide with the clearest separation of tetramer-positive cells in fluorescence-activated cell sorting (FACS) plots. Interestingly, the frequency of flu-epitope–restricted cells varied by diagnosis but not by age or prior vaccination with Pandemrix many years ago, reflecting the fact that almost everyone was exposed to pH1N1 within a few seasons past 2009 (50) and suggesting that the remaining pHA_273–287_ reactivity difference was intrinsic to narcolepsy. Isolating and functionally testing the corresponding TCRs for activation, we found large sequence diversity and shared motifs for pHA_273–287_ and NP_17–31_. Isolation of autoreactive HCRT_NH2_ clones was much more difficult, leading to smaller numbers than for the other antigens.

These results need to be interpreted in the context of the recent literature. Latorre et al. (41) were the first to report an increased T cell response against HCRT in NT1 versus controls. In this study, memory CD45RA^−^CD4^+^ T cells from 15 patients with NT1 and 3 patients with type 2 narcolepsy versus 12 controls were expanded polyclonally and screened for their capacity to proliferate in response to autologous B cells pulsed with an HCRT peptide pool, leading to the demonstration of an increased response to HCRT in narcolepsy (41). A similar experiment was also conducted with seasonal influenza A antigens; however, no differences were identified between narcolepsy versus controls. Leading on from these findings, 184 HCRT-specific CD4^+^ T cell clones isolated in nine patients were found to be of T helper 1 (Th1) and to target multiple epitopes of the prepro-HCRT peptide. Most of the isolated clones were DRB1 restricted, however, suggesting that the response was downstream of the disease process, as NT1 is primarily DQB1*06:02 associated. Additional cellular studies were also performed by Cogswell et al. (40), who observed a higher frequency of interferon-γ– and tumor necrosis factor-α–producing CD4^+^ (Th1) and CD8^+^ T cells in response to HCRT in 27 children with NT1 compared to 15 healthy control children, but no differences in 14 NT1 and 16 adult controls. In addition, priming with flu peptides amplified the T cell response to HCRT in children with NT1. Overall, these studies indicate that polyclonal CD4^+^ T cell responses to HCRT presented by multiple major histocompatibility complex class II molecules are increased in NT1 and, more specifically, in children close to disease onset.

Because NT1 is HLA-DQ0602 associated, Luo et al. (42) and Jiang et al. (39) specifically studied DQ0602-presented fragments of HCRT and corresponding T cells using tetramer studies. Luo et al. (42), studying 35 cases and 22 DQ0602 controls, found increased CD4^+^ T cells, recognizing C-terminal fragments of HCRT in cases, most notably when HCRT was amidated (HCRT_NH2_), a posttranslational modification that occurs in vivo in these peptides. In this study as well, responses were more strongly present in recent onset cases. A large number of flu antigen fragments binding to DQ0602 were also identified and tested, with the finding that increased T cell frequency recognizing pHA_273–287_, a pH1N1 2009 antigen, and NP_17–31,_ a PR8 (vaccine) peptide, was also found in NT1 versus controls. TCR sequencing in single cells was also performed and identified sharing of specific TRAJ24 and TRBV4-2 segments in HCRT_NH2_ and pHA_273–287_ TCR chains across flu peptides and hypocretin clones, suggesting cross-reactivity. Similarly, using 12 patients and 12 DQ0602 controls, Jiang et al. (39) isolated TCR clones carrying TRAJ24 using hypocretin-DQ0602 tetramer, finding that one such clone (TCR27) carried a cytotoxic CD4^+^ T cell phenotype, closely resembling T_EMRA_, a type of terminally differentiated CD4^+^ T cell with high expression of perforin and Granzyme B (51). In this study, the authors also found that the corresponding receptor was reactive to HCRT and HCRT_NH2_ when expressed in a Jurkat T cell model.

In the current study, complementing our previous results (42), we screened more NT1 cases (42 vs. 35) and a similar number of controls (22). We also rebalanced the sample so that similar proportions of TRAJ24 alleles (polymorphisms most strongly associated with NT1) were present across disease groups (Table 1). Most notably, we increased testing of subjects with TRAJ24 F/F, the genotype most strongly associated with NT1, expecting to isolate further clones with a TRAJ24F sequence. Importantly however, fewer early-onset (half, 5 vs. 10) and post-Pandemrix subjects (cases, 4 vs. 16; controls, 4 vs. 11) were included in this new sample (Table 1). Nonetheless, the results of 77 cases and 44 controls replicated our initial finding and, together with the literature, strongly suggest that a polyclonal CD4^+^ T cell response directed toward HCRT fragments that includes a subset with DQ0602 restriction is more prominent in NT1 cases than in matched controls.

We next studied functional effects of these T cell clones. Because NT1 is genetically associated with polymorphisms affecting TRAJ24, TRAJ28, and TRBV4-2 expression (42), all clones bearing these segments were prioritized for testing in J76 cells, even if found at low abundance. We thereby tested 121 TRAJ24, 22 TRAJ28, and 150 TRBV4-2 TCRs (out of the 709), finding that most of these isolated clones (108 TRAJ24, 20 TRAJ28, and 117 TRBV4-2) were negative when tested for activation (Dataset S6). Notably, these included 66 clones bearing the F/L allele of TRAV2-CAVETDSWGKL/FQF-TRAJ24, 24 TRBV4-2-CASSPDGTGVGNTIYF-TRBJ1-3, and 19 TRBV4-2-CASSQETQGRNYGYTF-TRBJ1-2 positive clones (Dataset S6). Despite this low yield, we identified a few distinct TRAJ24 and TRBV4-2 clones activated by pHA_273–287_, NP_17–31_, or HCRT_NH2_ (Dataset S6 and *SI Appendix*, Fig. S3); however, no cross-reactivity between antigens has been observed so far. This finding was disappointing considering our prior results (42).

Interestingly, only 11% of isolated HCRT_NH2_ tetramer TCR sequences reacted functionally to HCRT_NH2_ versus ∼50% of isolated pHA_273–287_, NP_17–31_ TCRs, explaining why we recovered fewer HCRT_NH2_ versus flu-activated TCRs. Staining patterns for each antigen were similar to those previously reported (42). Notably, tetramer-positive CD4^+^ T cells stained with pHA_273–287_ and NP_17–31_ were detected as subpopulations clearly separated from tetramer-negative CD4^+^ cells in almost every individual, suggesting high specificity (*SI Appendix*, Fig. S1). In contrast, HCRT_NH2_ staining was more tail-like with a few separated clusters, perhaps indicating polyclonality and less genuine selectivity to HCRT_NH2_ (*SI Appendix*, Fig. S1). This, together with the fact that activation was predicted by the presence of a larger amount of clonality in tetramer studies (>35 for HCRT_NH2_ vs. >5 times for flu), likely explains the lower yield.

Although these differences may be due primarily to lower affinities of isolated TCRs for HCRT_NH2_ versus flu antigens, other explanations may be involved. Indeed, given that expansion is dependent on activation, we would not expect needing higher amounts of clonality in cultures (35 vs. 5) to predict activation with HCRT_NH2_; thus, another possibility for these differences may be engagement of different downstream activation pathways. Indeed, the J76 cell line used in this study was engineered with nuclear factor of activated T cells (NFAT)-luciferase so that T cell activation using other pathways, such as activator protein–1, nuclear factor (NF)-κB, mammalian target of rapamycin, and other known pathways (52), was not explored. It is also possible that specific TCRs can bind without activation, although in this case, it would be difficult to explain how these clones could expand in culture.

Despite screening of a larger number of tetramers and dextramers (709 clones retrieved in both DQ0602 tetramer and dextramers were tested; see Dataset S6), limited cross-reactivity was detected across flu and HCRT_NH2_ antigens. Rather, many unique TCR clones were found (CDR3α: pHA_273–287_, 89; NP_17–31_, 80; HCRT_NH2_, 37; CDR3β: pHA_273–287_, 101; NP_17–31_, 85; HCRT_NH2_, 34) (Fig. 5 and *SI Appendix*, Fig. S8), making it possible to compare antigen-restricted sequences with previously published reference data (Fig. 5 and *SI Appendix*, Figs. S8 and S9) as well as between NT1 patients versus controls (*SI Appendix*, Fig. S9). Three HCRT_NH2_ clones (TCR101, 141, and 150) were found to weakly react to NP_17–31_ in initial experiments, but activation with NP_17–31_ was much weaker in replicate (Fig. 2 and Dataset S6), thus consolidating findings from J76 cells and suggesting that cross-reactivity is weak and unlikely to be of significance to the pathophysiology of disease onset.

Phenotyping of activated (and nonactivated) cells conducted for both tetramer- and dextramer-isolated cells also did not reveal significant differences across peptide (HCRT_NH2_ and pHA_273–287_ or NP_17–31_) specificity. These cells were characterized after peptide culture with low-dose interleukin-2; however, this commonly used procedure has been shown not to alter T cell phenotype (53, 54). Of note, many T_EMRA_ cytotoxic CD4^+^ cells were detected across all antigens, which was in line with results by Jiang and colleagues (39), who identified DQ6-restricted eTRAJ24L^+^ cells with higher levels of PRF1 and TGF-β in patients as opposed to controls. It is possible that TCR-binding tetramers without the ability to activate may exist and may be more frequent as a ratio of TCRs reactive to auto versus foreign antigens, since they would not be subjected to central tolerance. However, the literature on this subject remains inconclusive (55, 56). In all cases, it was notable that staining with HCRT_NH2_ was much higher than staining with native HCRT (42), a finding that was also concordant with results by Jiang et al. (39), who observed stronger signaling in a TCR transfectant (TCR27) with HCRT_87–97-NH2_. This implicates reduced tolerance toward posttranslationally modified peptides in the pathology of NT1, in line with evidence from previous studies, as well as other autoimmune diseases (42).

The observation that a large number of T cells reactive to HCRT in the context of DQ0602 presentation were T_EMRA_ cells is worth noting. These cells are a type of terminally differentiated CD4^+^ T cell expressing high levels of cytotoxic proteins (not unlike CD8^+^ T and NK cells) (55) and thus are likely able to directly mediate HCRT cell killing independent of CD8^+^ T cells. Markedly, several studies have recently suggested changes in T_EMRA_ populations in narcolepsy (57, 58).

Our experiments also allowed us to contrast the advantages of using dCODE dextramer versus tetramer. Since more streptavidin molecules are present on each backbone for dextramers, more CD4^+^ T cells were recognizable by dextramers for both foreign and HCRT_NH2_ (Fig. 1 and *SI Appendix*, Fig. S1). Critically, however, this also increased background for nonspecific CD4^+^ T cells and reduced enrichment (Datasets S5 and S7), making it a challenge to attribute each clone to each unique antigen using multiple DNA barcodes to each dextramer-stained CD4^+^ T cell (Dataset S5). In comparison with tetramer, dextramer-retrieved TCRs were often labeled with multiple antigens and were found to be activated by a different peptide than the one assigned by its barcode (Table 2 and Dataset S7). We hypothesize that this is an artifact due to the pooling of T cells for sequencing and suggest that loading and sequencing each dCODE dextramer reaction in separate lanes could produce better results. As a consequence, clones retrieved by dextramer experiments could not be analyzed by peptide specificity unless they showed activation in Jurkat cell lines by a single peptide. Analysis of the phenotypes of activated (Fig. 6) and nonactivated clones (for tetramer only, to keep ligand specificity) did not reveal differences across NT1 and controls, although interestingly, HCRT_NH2_-recognizing T cells were predominantly T_EMRA_ cells rather than Tregs, thus suggesting they may play a role in the pathophysiology of NT1.

Our study contributes to the understanding of T cell–mediated reactivity in narcolepsy by extending previous findings related to both epitope-restricted reactivity of CD4^+^ T cells and phenotypic characterization of reactive cells. The interplay between flu antigens and HCRT in autoimmunity remains enigmatic, and our study supports that the pathophysiological mechanisms that may explain their relationship likely extend beyond molecular mimicry and immune cell cross-reactivity. Evidence in support of the T cell–mediated killing of HCRT cells is increasing, whereby the exact roles of CD4^+^ and CD8^+^ cells in this trajectory remain to be deciphered. Studies in mice have shown that both pathogenic CD4^+^ Th1 cells, as well as cytotoxic CD8^+^ T cells, infiltrate the hypothalamus; however, destruction of HCRT neurons was specifically mediated by only the latter (43). Concurrently, Pedersen et al. (44) investigated CD8^+^ T cells, also finding HCRT-targeting cells that were, however, not exclusive to the patient population and could also be identified in healthy controls. Interestingly, however, CD4^+^ T cells with a cytotoxic potential may be cardinally implicated in this process; hence, with possible advances in single-cell sequencing technology and more extensive phenotypic characterization of T cell subtypes beyond TCR diversity, we can expect important new insights to the specificities and functions of these cells in the near future.

## Materials and Methods

This study was reviewed and approved by the Stanford University Institutional Review Board (Protocol # 14325, Registration # 5136). Informed consent was obtained from each participant. Detailed information on subjects is described in the *SI Appendix*. Details of vaccine, peptides, tetramer staining, TCR sequencing, and network analysis are provided in the *SI Appendix* and Datasets S1–S11.

## Supplementary Material

Supplementary File

Supplementary File

Supplementary File

Supplementary File

Supplementary File

Supplementary File

Supplementary File

Supplementary File

Supplementary File

Supplementary File

Supplementary File

Supplementary File

## Data Availability

All study data are included in the article and/or supporting information.

## References

[1] S. Nishino, T. Kanbayashi, Symptomatic narcolepsy, cataplexy and hypersomnia, and their implications in the hypothalamic hypocretin/orexin system. Sleep Med. Rev. 9, 269–310 (2005). 16006155 10.1016/j.smrv.2005.03.004

[2] C. Peyron , A mutation in a case of early onset narcolepsy and a generalized absence of hypocretin peptides in human narcoleptic brains. Nat. Med. 6, 991–997 (2000). 10973318 10.1038/79690

[3] T. C. Thannickal , Reduced number of hypocretin neurons in human narcolepsy. Neuron 27, 469–474 (2000). 11055430 10.1016/s0896-6273(00)00058-1 PMC8760623

[4] F. Han , Genome wide analysis of narcolepsy in China implicates novel immune loci and reveals changes in association prior to versus after the 2009 H1N1 influenza pandemic. PLoS Genet. 9, e1003880 (2013). 24204295 10.1371/journal.pgen.1003880 PMC3814311

[5] J. Faraco , ImmunoChip study implicates antigen presentation to T cells in narcolepsy. PLoS Genet. 9, e1003270 (2013). 23459209 10.1371/journal.pgen.1003270 PMC3573113

[6] B. R. Kornum, J. Faraco, E. Mignot, Narcolepsy with hypocretin/orexin deficiency, infections and autoimmunity of the brain. Curr. Opin. Neurobiol. 21, 897–903 (2011). 21963829 10.1016/j.conb.2011.09.003

[7] H. Hor , Genome-wide association study identifies new HLA class II haplotypes strongly protective against narcolepsy. Nat. Genet. 42, 786–789 (2010). Correction in: *Nat. Genet.* **43**, 388 (2011). 20711174 10.1038/ng.647

[8] J. Hallmayer , Narcolepsy is strongly associated with the T-cell receptor alpha locus. Nat. Genet. 41, 708–711 (2009). Correction in: *Nat. Genet.* **41**, 859 (2009). 19412176 10.1038/ng.372 PMC2803042

[9] H. M. Ollila, M. Fernandez-Vina, E. Mignot, HLA-DQ allele competition in narcolepsy: A comment on Tafti et al. DQB1 locus alone explains most of the risk and protection in narcolepsy with cataplexy in Europe. Sleep (Basel) 38, 147–151 (2015). 10.5665/sleep.4342 PMC4262948 25325462

[10] M. Vringer, B. R. Kornum, Emerging therapeutic targets for narcolepsy. Expert Opin. Ther. Targets, 10.1080/14728222.2021.1969361 (2021). 34402358

[11] F. J. Martinez-Orozco, J. L. Vicario, C. De Andres, M. Fernandez-Arquero, R. Peraita-Adrados, Comorbidity of narcolepsy type 1 with autoimmune diseases and other immunopathological disorders: A case-control study. J. Clin. Med. Res. 8, 495–505 (2016). 27298657 10.14740/jocmr2569w PMC4894018

[12] E. Feketeova , Narcolepsy in Slovakia – Epidemiology, clinical and polysomnographic features, comorbid diagnoses: A case-control study. Sleep Med. 67, 15–22 (2020). 31884306 10.1016/j.sleep.2019.10.012

[13] T. Y. Chen , The association between asthma and narcolepsy: A nationwide case-control study in Taiwan. Nat. Sci. Sleep 13, 1631–1640 (2021). 34584477 10.2147/NSS.S317746 PMC8464343

[14] A. Aran , Elevated anti-streptococcal antibodies in patients with recent narcolepsy onset. Sleep 32, 979–983 (2009). 19725248 10.1093/sleep/32.8.979 PMC2717204

[15] W. T. Longstreth Jr. , Prevalence of narcolepsy in King County, Washington, USA. Sleep Med. 10, 422–426 (2009). 19013100 10.1016/j.sleep.2008.05.009 PMC2754568

[16] F. Han , Narcolepsy onset is seasonal and increased following the 2009 H1N1 pandemic in China. Ann. Neurol. 70, 410–417 (2011). 21866560 10.1002/ana.22587

[17] H. Wu , Symptoms and occurrences of narcolepsy: A retrospective study of 162 patients during a 10-year period in eastern China. Sleep Med. 15, 607–613 (2014). 24767723 10.1016/j.sleep.2013.12.012

[18] D. Weibel , Narcolepsy and adjuvanted pandemic influenza A (H1N1) 2009 vaccines— Multi-country assessment. Vaccine 36, 6202–6211 (2018). 30122647 10.1016/j.vaccine.2018.08.008 PMC6404226

[19] T. J. Dye, N. Gurbani, N. Simakajornboon, Epidemiology and pathophysiology of childhood narcolepsy. Paediatr. Respir. Rev. 25, 14–18 (2018). 28108192 10.1016/j.prrv.2016.12.005

[20] T. Sarkanen, A. Alakuijala, I. Julkunen, M. Partinen, Narcolepsy associated with Pandemrix vaccine. Curr. Neurol. Neurosci. Rep. 18, 43 (2018). 29855798 10.1007/s11910-018-0851-5

[21] H. Nohynek , AS03 adjuvanted AH1N1 vaccine associated with an abrupt increase in the incidence of childhood narcolepsy in Finland. PLoS One 7, e33536 (2012). 22470453 10.1371/journal.pone.0033536 PMC3314666

[22] M. Partinen , Increased incidence and clinical picture of childhood narcolepsy following the 2009 H1N1 pandemic vaccination campaign in Finland. PLoS One 7, e33723 (2012). 22470463 10.1371/journal.pone.0033723 PMC3314680

[23] L. Jacob , Comparison of Pandemrix and Arepanrix, two pH1N1 AS03-adjuvanted vaccines differentially associated with narcolepsy development. Brain Behav. Immun. 47 (suppl. C), 44–57 (2015). 25452148 10.1016/j.bbi.2014.11.004

[24] F. Han , HLA-DQ association and allele competition in Chinese narcolepsy. Tissue Antigens 80, 328–335 (2012). 22862152 10.1111/j.1399-0039.2012.01948.x

[25] T. Miyagawa , New susceptibility variants to narcolepsy identified in HLA class II region. Hum. Mol. Genet. 24, 891–898 (2015). 25256355 10.1093/hmg/ddu480

[26] E. Mignot , Complex HLA-DR and -DQ interactions confer risk of narcolepsy-cataplexy in three ethnic groups. Am. J. Hum. Genet. 68, 686–699 (2001). 11179016 10.1086/318799 PMC1274481

[27] M. Tafti , DQB1 locus alone explains most of the risk and protection in narcolepsy with cataplexy in Europe. Sleep (Basel) 37, 19–25 (2014). 10.5665/sleep.3300 PMC3865351 24381371

[28] T. Bedford, S. Cobey, P. Beerli, M. Pascual, Global migration dynamics underlie evolution and persistence of human influenza A (H3N2). PLoS Pathog. 6, e1000918 (2010). 20523898 10.1371/journal.ppat.1000918 PMC2877742

[29] H. Ollila , Narcolepsy risk loci are enriched in immune cells and suggest autoimmune modulation of the T cell receptor repertoire. *bioRxiv* [Preprint] (2018). 10.1101/373555.

[30] B. R. Kornum , Absence of autoreactive CD4^+^ T-cells targeting HLA-DQA1*01:02/DQB1*06:02 restricted hypocretin/orexin epitopes in narcolepsy type 1 when detected by EliSpot. J. Neuroimmunol. 309, 7–11 (2017). 28601291 10.1016/j.jneuroim.2017.05.001

[31] M. Ramberger , CD4^+^ T-cell reactivity to orexin/hypocretin in patients with narcolepsy type 1. Sleep (Basel) 40, zsw070 (2017). 10.1093/sleep/zsw070 PMC5806576 28364420

[32] S. Tanaka, Y. Honda, Y. Inoue, M. Honda, Detection of autoantibodies against hypocretin, hcrtrl, and hcrtr2 in narcolepsy: Anti-Hcrt system antibody in narcolepsy. Sleep 29, 633–638 (2006). 16774153 10.1093/sleep/29.5.633

[33] V. Cvetkovic-Lopes , Elevated Tribbles homolog 2-specific antibody levels in narcolepsy patients. J. Clin. Invest. 120, 713–719 (2010). 20160349 10.1172/JCI41366 PMC2827962

[34] A. Lind , A/H1N1 antibodies and TRIB2 autoantibodies in narcolepsy patients diagnosed in conjunction with the Pandemrix vaccination campaign in Sweden 2009–2010. J. Autoimmun. 50, 99–106 (2014). 24485154 10.1016/j.jaut.2014.01.031

[35] S. S. Ahmed , Antibodies to influenza nucleoprotein cross-react with human hypocretin receptor 2. Sci. Transl. Med. 7, 294ra105 (2015). 10.1126/scitranslmed.aab2354 26136476

[36] M. P. Giannoccaro , Antibodies against hypocretin receptor 2 are rare in narcolepsy. Sleep (Basel) 40, zsw056 (2017). 10.1093/sleep/zsw056 28364500

[37] G. Luo , Absence of anti-hypocretin receptor 2 autoantibodies in post Pandemrix narcolepsy cases. PLoS One 12, e0187305 (2017). 29220370 10.1371/journal.pone.0187305 PMC5722318

[38] A. van der Heide, I. M. Hegeman-Kleinn, E. Peeters, G. J. Lammers, R. Fronczek, Immunohistochemical screening for antibodies in recent onset type 1 narcolepsy and after H1N1 vaccination. J. Neuroimmunol. 283, 58–62 (2015). 26004157 10.1016/j.jneuroim.2015.04.008

[39] W. Jiang , In vivo clonal expansion and phenotypes of hypocretin-specific CD4^+^ T cells in narcolepsy patients and controls. Nat. Commun. 10, 5247 (2019). 31748512 10.1038/s41467-019-13234-x PMC6868281

[40] A. C. Cogswell , Children with narcolepsy type 1 have increased T-cell responses to orexins. Ann. Clin. Transl. Neurol. 6, 2566–2572 (2019). 31730293 10.1002/acn3.50908 PMC6917326

[41] D. Latorre , T cells in patients with narcolepsy target self-antigens of hypocretin neurons. Nature 562, 63–68 (2018). 30232458 10.1038/s41586-018-0540-1

[42] G. Luo , Autoimmunity to hypocretin and molecular mimicry to flu in type 1 narcolepsy. Proc. Natl. Acad. Sci. U.S.A. 115, E12323–E12332 (2018). 30541895 10.1073/pnas.1818150116 PMC6310865

[43] R. Bernard-Valnet , CD8 T cell-mediated killing of orexinergic neurons induces a narcolepsy-like phenotype in mice. Proc. Natl. Acad. Sci. U.S.A. 113, 10956–10961 (2016). 27621438 10.1073/pnas.1603325113 PMC5047186

[44] N. W. Pedersen , CD8^+^ T cells from patients with narcolepsy and healthy controls recognize hypocretin neuron-specific antigens. Nat. Commun. 10, 837 (2019). 30783092 10.1038/s41467-019-08774-1 PMC6381094

[45] W. T. Longstreth Jr., T. G. Ton, T. D. Koepsell, Narcolepsy and streptococcal infections. Sleep 32, 1548 (2009). 20041589 10.1093/sleep/32.12.1548 PMC2786037

[46] H. Huang, C. Wang, F. Rubelt, T. J. Scriba, M. M. Davis, Analyzing the *Mycobacterium tuberculosis* immune response by T-cell receptor clustering with GLIPH2 and genome-wide antigen screening. Nat. Biotechnol. 38, 1194–1202 (2020). 32341563 10.1038/s41587-020-0505-4 PMC7541396

[47] M. P. Lefranc, Unique database numbering system for immunogenetic analysis. Immunol. Today 18, 509 (1997). 9386342 10.1016/s0167-5699(97)01163-8

[48] A. Han, J. Glanville, L. Hansmann, M. M. Davis, Linking T-cell receptor sequence to functional phenotype at the single-cell level. Nat. Biotechnol. 32, 684–692 (2014). 24952902 10.1038/nbt.2938 PMC4337815

[49] L. McInnes, J. Healy, N. Saul, L. Großberger, UMAP: Uniform manifold approximation and projection. J. Open Source Softw. 3, 861 (2018).

[50] J. McVernon , Seroprevalence of antibody to influenza A(H1N1)pdm09 attributed to vaccination or infection, before and after the second (2010) pandemic wave in Australia. Influenza Other Respir. Viruses 8, 194–200 (2014). 24382379 10.1111/irv.12225 PMC4186467

[51] E. Cano-Gamez , Single-cell transcriptomics identifies an effectorness gradient shaping the response of CD4^+^ T cells to cytokines. Nat. Commun. 11, 1801 (2020). 32286271 10.1038/s41467-020-15543-y PMC7156481

[52] J. R. Hwang, Y. Byeon, D. Kim, S. G. Park, Recent insights of T cell receptor-mediated signaling pathways for T cell activation and development. Exp. Mol. Med. 52, 750–761 (2020). 32439954 10.1038/s12276-020-0435-8 PMC7272404

[53] H. Uchtenhagen , Efficient ex vivo analysis of CD4+ T-cell responses using combinatorial HLA class II tetramer staining. Nat. Commun. 7, 12614 (2016). 27571776 10.1038/ncomms12614 PMC5013714

[54] Y. Lin , Optimization and validation of a robust human T-cell culture method for monitoring phenotypic and polyfunctional antigen-specific CD4 and CD8 T-cell responses. Cytotherapy 11, 912–922 (2009). 19903103 10.3109/14653240903136987 PMC2932850

[55] T. P. Riley , T cell receptor cross-reactivity expanded by dramatic peptide-MHC adaptability. Nat. Chem. Biol. 14, 934–942 (2018). 30224695 10.1038/s41589-018-0130-4 PMC6371774

[56] L. V. Sibener , Isolation of a structural mechanism for uncoupling T cell receptor signaling from peptide-MHC binding. Cell 174, 672–687.e27 (2018). 30053426 10.1016/j.cell.2018.06.017 PMC6140336

[57] M. Moresco , Flow cytometry T cell profiling in a recent-onset narcoleptic type 1 child: A case report. Sleep Med. 68, 21–23 (2020). 32018187 10.1016/j.sleep.2019.08.017

[58] M. Moresco , Flow cytometry analysis of T-cell subsets in cerebrospinal fluid of narcolepsy type 1 patients with long-lasting disease. Sleep Med. 44, 53–60 (2018). 29530370 10.1016/j.sleep.2017.11.1150

