# Supplementary material for: Characterization of T cell receptors reactive to HCRTNH2, pHA273-287, and NP17-31 in control and narcolepsy patients
Source: Proc Natl Acad Sci U S A. 2022 Aug 1;119(32):e2205797119. doi: 10.1073/pnas.2205797119 (PMC9371724; doi:10.1073/pnas.2205797119)
Supplement: Supplementary File [file pnas.2205797119.sapp.pdf]

PNAS
www.pnas.org

Supplementary Information for

Characterization of T cell receptors reactive to $HCRT_{NH2}$, $pHA_{273-287}$ and NP_{17-31} in control and narcolepsy patients

Guo Luo[1], Jing Zhang[1], Ling Lin[1], Emmanuel Jean-Marie Mignot[1]

[1] Center for Sleep Sciences and Medicine, Stanford University School of Medicine, Palo Alto CA 94304, USA.

* Correspondence should be addressed to: Emmanuel Mignot, Director, Center For Sleep Sciences and Medicine, 3165 Porter Drive, #2178, Palo Alto CA 94304, USA. (1)650-725-6517 (tel), (1)650-725-7341 (fax), mignot@stanford.edu.

This PDF file includes:

Supplementary Methods

Figures S1 to S11

SI references

Other supplementary materials for this manuscript include the following:

Datasets S1 to S9

Supplementary Methods

Participants

In this extended research, all narcolepsy patients with cataplexy (n = 42; 61.9% female; median age with range: 19.9 [7.5-89.8] years) meet criteria for International Classification of Sleep Disorders 3 (ICSD 3) for type 1 narcolepsy (NT1). Disease onset in 4 out of 42 (9.5%) patients followed vaccination with Pandemrix® (Table 1). Healthy controls (n = 22; 11% female; median age with range: 30.7 [5.7-64.6]) were unrelated subjects. 4 out of 22 (18.2%) controls were vaccinated with Pandemrix®. All patients and controls were DQB1*06:02 positive. Peripheral blood mononuclear cells (PBMCs) were collected by apheresis at the Stanford blood center or through phlebotomy, followed by ficoll isolation and storage in liquid nitrogen until use.

Vaccine

The Pandemrix® vaccine (A/California/7/2009 (H1N1) NYMC X-179A monovalent bulk (inactivated, sterile)) (Batch# AFLSFDA280) used throughout this study was manufactured with HA content at 139 µg/ml (determined with single radial diffusion (SRD)) by GlaxoSmithKline (GSK) Dresden in January 2010. This batch has been used during the 2009-2010 vaccination campaign in Europe. Summary of Pandemrix® characteristics can be found in European medicines agency document

(http://www.ema.europa.eu/docs/en_GB/document_library/Other/2010/05/WC500091295.pdf).

Peptides

Peptides were synthesized with >95% purity at GenScript NJ and dissolved in dimethyl sulfoxide (DMSO) at a stock concentration of 10 mM. Their binding affinity to DQ0602 was previously reported(1) (Dataset S1). Of note, 17 additional peptides were tested with dCODE® dextramer DQ0602 (Dataset S1 and Fig. S1B).

Generation of tetramer and dextramer DQ0602

Peptide exchange and generation of class II tetramer was previously described(1-4). Briefly, biotinylated DQ0602 bound with peptide was incubated with phycoerythrin (PE) conjugated streptavidin (Cat# 405204, BioLegend) or custome dCODE® Dextramer backbone (Immudex). This reaction was cleaned up by removal of extra DQ0602. Tetramer and dextramer DQ0602 was concentrated and stored in phosphate buffered saline (PBS, pH=7.4). Before staining with cells, aggregation, if any, was removed by centrifugation.

Tetramer and dextramer DQ0602 sorting

Tetramer staining and single-cell sorting was previously described(1-5). Briefly, PBMCs were stimulated with100 ng/ml (final concentration of HA) Pandemrix® in a 50 ml-culture flask (2.5×10^6 cells/ml) or 6.25 μM peptide in a 96-well plate ($1-2.5\times10^6$ cells/ml) for 10 days at 37°C, 5% CO_2. 20 IU/ml IL-2 (Cat# 14-8029-81,

eBiosciences) was supplemented from day 7 in complete RPMI medium (RPMI (Cat# 61870-036, Gibco) supplemented with 10% fetal bovine serum (FBS) and 1% penicillin/streptomycin). Medium supplemented with Pandemrix® or peptide was changed every 2-4 days. Cell culture was harvested and incubated with multimer DQ0602 in 100 μl of complete RPMI medium for 90 min at 37°C, 5% CO_2 and then stained with a combination of Brilliant Violet 421® (BV421) anti-CD3, Alexa Fluor® 488 (AF488) anti-CD4, Alexa Fluor® 700 (AF700) anti-CD8, Brilliant Violet 605® (BV605) anti-CD45RA and APC/Cy7 anti-CD45RO antibodies (all from BioLegend), followed by single-cell sorting in 96-well plates for tetramer or bulk sorting for dextramer with BD ARIA II at Stanford shared FACS facility (SSFF). Propidium iodide (PI) was added to separate live cells. Data were analyzed using FlowJo (v10.0.8r1) (See Fig. S1 and Dataset S11(1) for FACS).

Single-cell TCR sequencing

Single-cell paired TCR sequencing was performed as described(1, 4, 6, 7). Briefly, single cell of CD3+CD4+CD8-Tetramer+ was sorted directly into OneStep® reverse transcription polymerase chain reaction (RT-PCR) buffer (Cat# 210215, Qiagen) in a 96-well plate. Paired TCR CDR3 sequences were obtained by a series of three nested PCR reactions and sequenced on MiSeq platform for a pair-ended 2x250 run. Fastq files were analyzed with a customized TCRseq pipeline at the Human Immune Monitoring Center (HIMC) at Stanford University. Further analysis was performed with R (R Core Team, 2020)(8).

10x sequencing

Single Cell 5' Library was prepared by following the instruction of Chromium Next GEM Single Cell V(D)J Reagent Kits v1.1 with Feature Barcode technology for Cell Surface Protein (https://support.10xgenomics.com/single-cell-vdj/library-prep/doc/user-guide-chromium-single-cell-vdj-reagent-kits-user-guide-v11-chemistry-with-feature-barcoding-technology-for-cell-surface-protein). Briefly, individual PMBCs were cultured and harvested as above. Cell cultures stimulated with the same peptide were pooled and stained by cognate peptide-dCODE® dextramer DQ0602 with a specific DNA barcode. dCODE® dextramer+CD3+CD4+CD8- T cells were sorted into complete RPMI medium in a 1.5 ml tube and loaded in 10x chip. Three libraries of gene expression, TCR VDJ and cell surface barcode were constructed and sequenced on a HiSeq4000 platform at Stanford Genomics for a paired end 2x150 run with a depth of >20,000, 5,000, 5,000 read pairs per cell, respectively. Sequencing data were processed using the Cell Ranger (v6.0, 10x Genomics). Single cell was identified using demuxlet(9). Only singlets with >200 total features were used for further analysis with R (R Core Team, 2020)(8). The highest unique molecular identifier (UMI) count of cell surface barcode (peptide) was used to identify each singlet (if same, removal). Of note, one TCR clonotype from different cells may have different peptides.

Phenotype Clustering

10x sequencing results were imported into R (R Core Team, 2020)(8) and analyzed using Seurat (v4)(10-14). Cells with less than 250 genes or 500 UMI counts detected, with more than 5% mitochondrial genes, or more than 6.3 genes pre UMI were removed from the data set. Myeloid cells, B cells, TRBV7-9, TRBV7-2, TRBV19, TRBV6-5, TRBV5-1, class II HLA and TRBV20-1 cells were also removed, as they were likely from contamination or affect downstream analysis. Genes of CD8, haemoglobin, MS4A1, immunoglobulin, TCR, tubulin and long intergenic non-protein coding RNA were removed from list of features. Counts were normalized. Each cell was assigned to a respective stage of cell cycle using a publicly available list of cell cycle genes(15). Next, cell cycle, mitochondrial content and UMI size were regressed out using Seurat's SCTransform. Highly variable genes were identified and used to perform principal component analysis (PCA). The first 20 principal components were used as an input for SNN clustering and for embedding using the uniform manifold approximation and projection (UMAP). Marker genes for each cluster were identified computationally using default Seurat setting. Clusters were manually annotated according to their gene expression pattern.

Luciferase activity

Transfected TCR activation was previously reported(4, 7). Briefly, TCRαβ chains tagged with GFP were cloned to N103 vector (a gift from Dr. Mark Davis) by IDT (Integrated DNA Technologies, Inc.). Jurkat 76 (J76)-NFATRE-luc cells (a gift from Dr. Mark Davis) were transfected with TCR using the Lipofectamine 3000 reagent

(Cat# L3000015, Invitrogen). TCR positive cells were sorted using anti-human TCRα/β antibody (Cat# 306702, BioLegend). In 200 µl of complete RPMI medium, 0.1 million of J76-TCR cells were co-cultured with 0.1 million of RM3-DQ0602(16) cells at the presence of 10 µM of pHA273-287, NP17-31, HCRT54-66-NH2, HCRT86-97-NH2, or RFX4-43, or 0.2 µl DMSO for 8 hours. Luciferase activity was detected with Nano-Glo Luciferase Assay Kit (Cat# N1130, Promega). Each co-culture was triplicated.

Statistical analyses

Two-tailed Mann-Whitney U test was used to compare frequency of antigen specific CD4+ T cells between narcolepsy patients and healthy controls and results were plotted using R (R Core Team, 2020)(8). Student t test was used for luciferase activity of transgenic TCR in J76 cells and for the mean expression of tetramer isolated TCR clones. Phylogenetic trees and networks were constructed using R package msa(17) and igraph(18), respectively. Sequence logos were created with R package ggseqlogo(19) and IceLogo(20) using negative TCRs or public CD4+ TCRs as reference(7, 21-39). P-value <0.05 was considered statistically significant. *, $p < 0.05$; **, $p < 0.01$; ***, $p < 0.001$; ns, not significant.

Supplementary Figures

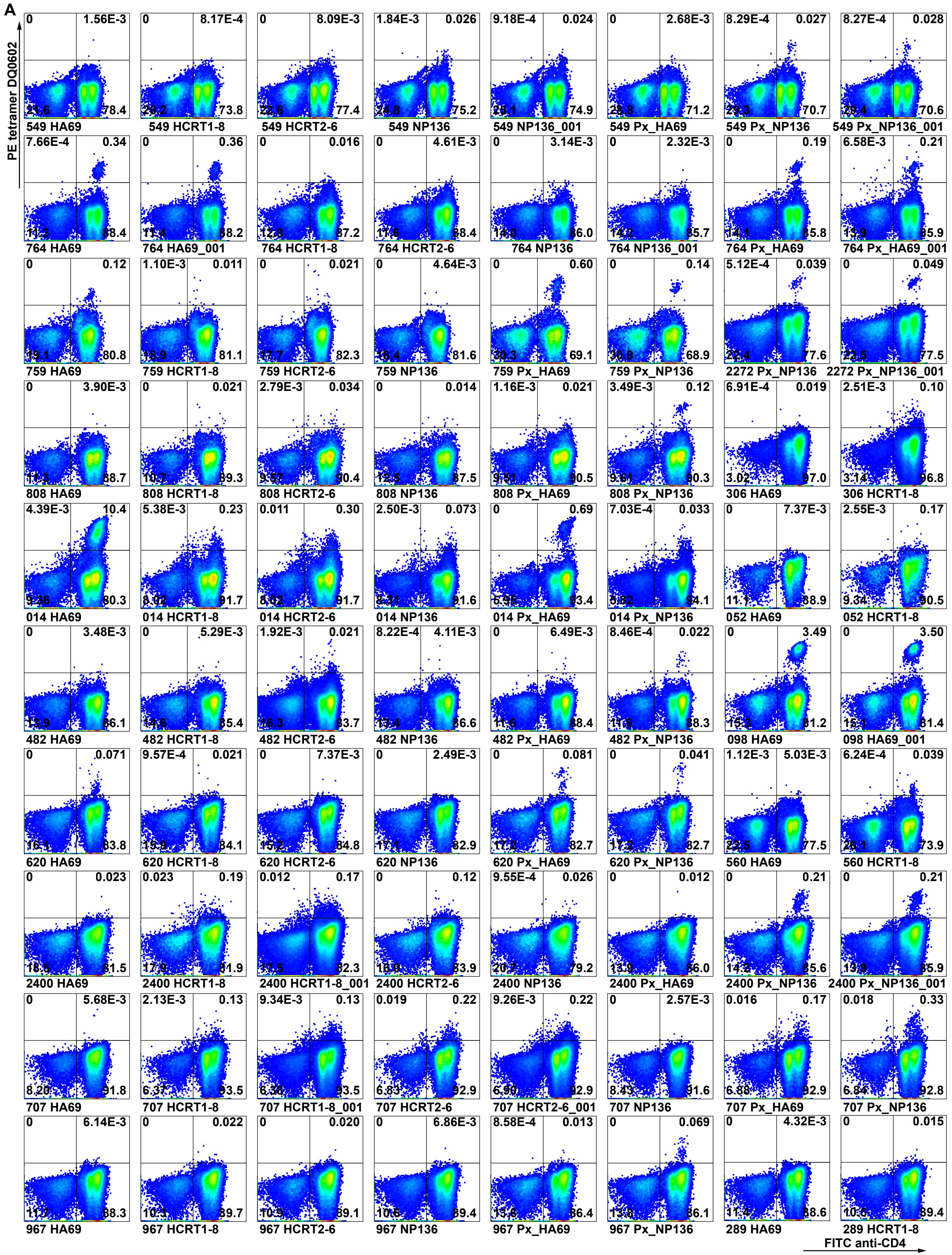
A
PE tetramer DQ0602
FITC anti-CD4
0 1.56E-3 21.6 78.4 549 HA69
0 8.17E-4 26.2 73.8 549 HCRT1-8
0 8.09E-3 22.6 77.4 549 HCRT2-6
1.84E-3 0.026 24.8 75.2 549 NP136
9.18E-4 0.024 25.1 74.9 549 NP136_001
0 2.68E-3 28.8 71.2 549 Px_HA69
8.29E-4 0.027 29.3 70.7 549 Px_NP136
8.27E-4 0.028 29.4 70.6 549 Px_NP136_001
7.66E-4 0.34 11.3 88.4 764 HA69
0 0.36 11.4 88.2 764 HA69_001
0 0.016 12.8 87.2 764 HCRT1-8
0 4.61E-3 11.6 88.4 764 HCRT2-6
0 3.14E-3 14.0 86.0 764 NP136
0 2.32E-3 14.2 85.7 764 NP136_001
0 0.19 14.1 85.8 764 Px_HA69
6.58E-3 0.21 13.9 85.9 764 Px_HA69_001
0 0.12 19.1 80.8 759 HA69
1.10E-3 0.011 18.9 81.1 759 HCRT1-8
0 0.021 17.7 82.3 759 HCRT2-6
0 4.64E-3 18.4 81.6 759 NP136
0 0.60 30.3 69.1 759 Px_HA69
0 0.14 30.9 68.9 759 Px_NP136
5.12E-4 0.039 22.4 77.6 2272 Px_NP136
0 0.049 22.5 77.5 2272 Px_NP136_001
0 3.90E-3 11.3 88.7 808 HA69
0 0.021 10.7 89.3 808 HCRT1-8
2.79E-3 0.034 9.57 90.4 808 HCRT2-6
0 0.014 12.5 87.5 808 NP136
1.16E-3 0.021 9.51 90.5 808 Px_HA69
3.49E-3 0.12 9.61 90.3 808 Px_NP136
6.91E-4 0.019 3.02 97.0 306 HA69
2.51E-3 0.10 3.14 96.8 306 HCRT1-8
4.39E-3 10.4 9.36 80.3 014 HA69
5.38E-3 0.23 8.02 91.7 014 HCRT1-8
0.011 0.30 8.02 91.7 014 HCRT2-6
2.50E-3 0.073 8.31 91.6 014 NP136
0 0.69 5.95 93.4 014 Px_HA69
7.03E-4 0.033 5.82 94.1 014 Px_NP136
0 7.37E-3 11.1 88.9 052 HA69
2.55E-3 0.17 9.34 90.5 052 HCRT1-8
0 3.48E-3 13.9 86.1 482 HA69
0 5.29E-3 14.6 85.4 482 HCRT1-8
1.92E-3 0.021 16.3 83.7 482 HCRT2-6
8.22E-4 4.11E-3 13.4 86.6 482 NP136
0 6.49E-3 11.6 88.4 482 Px_HA69
8.46E-4 0.022 11.6 88.3 482 Px_NP136
0 3.49 15.3 81.2 098 HA69
0 3.50 15.1 81.4 098 HA69_001
0 0.071 16.1 83.8 620 HA69
9.57E-4 0.021 15.9 84.1 620 HCRT1-8
0 7.37E-3 15.2 84.8 620 HCRT2-6
0 2.49E-3 17.1 82.9 620 NP136
0 0.081 17.2 82.7 620 Px_HA69
0 0.041 17.2 82.7 620 Px_NP136
1.12E-3 5.03E-3 22.5 77.5 560 HA69
6.24E-4 0.039 26.1 73.9 560 HCRT1-8
0 0.023 18.5 81.5 2400 HA69
0.023 0.19 17.9 81.9 2400 HCRT1-8
0.012 0.17 17.5 82.3 2400 HCRT1-8_001
0 0.12 16.0 83.9 2400 HCRT2-6
9.55E-4 0.026 20.7 79.2 2400 NP136
0 0.012 13.9 86.0 2400 Px_HA69
0 0.21 14.2 85.6 2400 Px_NP136
0 0.21 13.9 85.9 2400 Px_NP136_001
0 5.68E-3 8.20 91.8 707 HA69
2.13E-3 0.13 6.37 93.5 707 HCRT1-8
9.34E-3 0.13 6.38 93.5 707 HCRT1-8_001
0.019 0.22 6.83 92.9 707 HCRT2-6
9.26E-3 0.22 6.90 92.9 707 HCRT2-6_001
0 2.57E-3 8.43 91.6 707 NP136
0.016 0.17 6.88 92.9 707 Px_HA69
0.018 0.33 6.84 92.8 707 Px_NP136
0 6.14E-3 11.7 88.3 967 HA69
0 0.022 10.3 89.7 967 HCRT1-8
0 0.020 10.9 89.1 967 HCRT2-6
0 6.86E-3 10.6 89.4 967 NP136
8.58E-4 0.013 13.6 86.4 967 Px_HA69
0 0.069 13.8 86.1 967 Px_NP136
0 4.32E-3 11.4 88.6 289 HA69
0 0.015 10.6 89.4 289 HCRT1-8

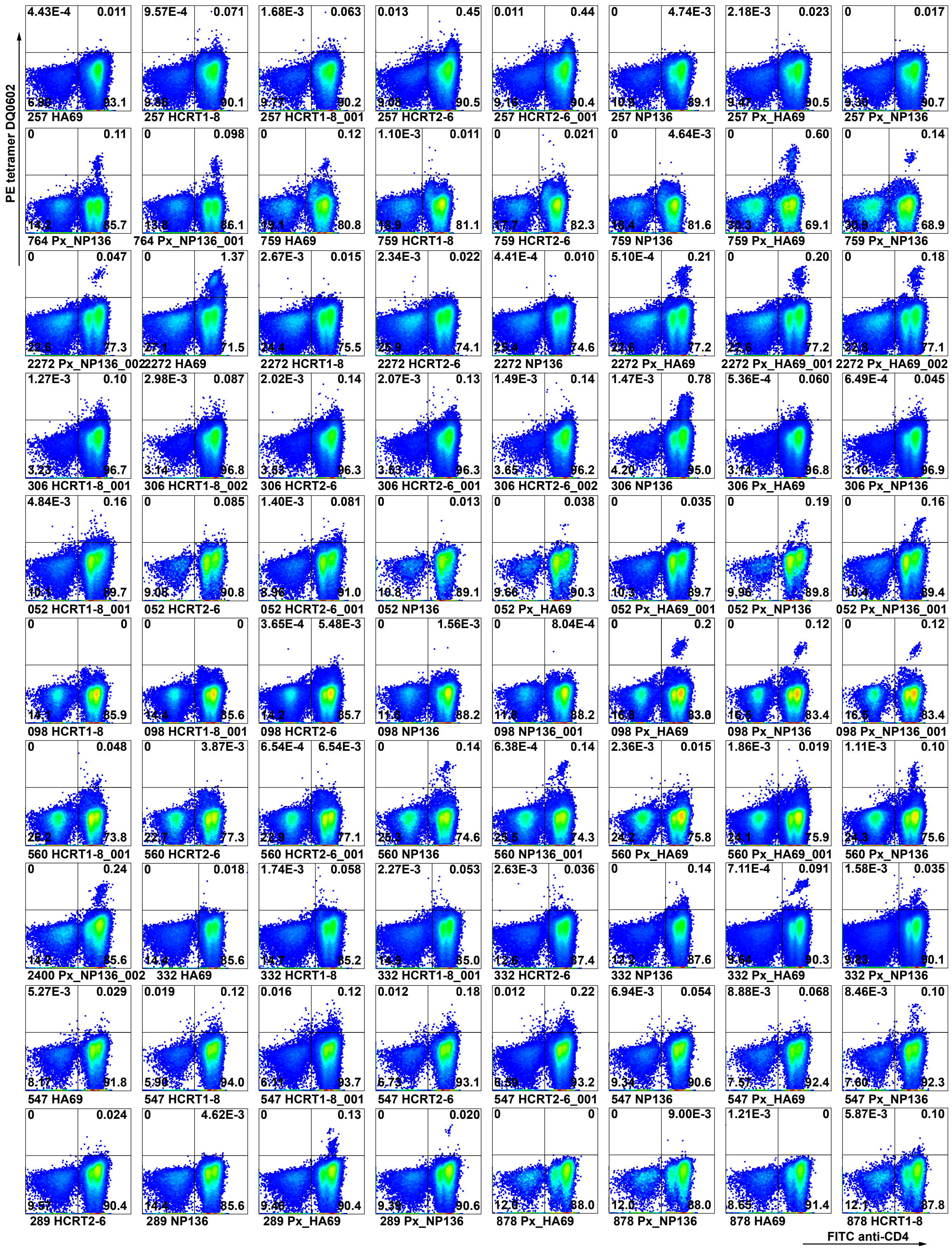
PE tetramer DQ0602
FITC anti-CD4
257 HA69
257 HCRT1-8
257 HCRT1-8_001
257 HCRT2-6
257 HCRT2-6_001
257 NP136
257 Px_HA69
257 Px_NP136
764 Px_NP136
764 Px_NP136_001
759 HA69
759 HCRT1-8
759 HCRT2-6
759 NP136
759 Px_HA69
759 Px_NP136
2272 Px_NP136_002
2272 HA69
2272 HCRT1-8
2272 HCRT2-6
2272 NP136
2272 Px_HA69
2272 Px_HA69_001
2272 Px_HA69_002
306 HCRT1-8_001
306 HCRT1-8_002
306 HCRT2-6
306 HCRT2-6_001
306 HCRT2-6_002
306 NP136
306 Px_HA69
306 Px_NP136
052 HCRT1-8_001
052 HCRT2-6
052 HCRT2-6_001
052 NP136
052 Px_HA69
052 Px_HA69_001
052 Px_NP136
052 Px_NP136_001
098 HCRT1-8
098 HCRT1-8_001
098 HCRT2-6
098 NP136
098 NP136_001
098 Px_HA69
098 Px_NP136
098 Px_NP136_001
560 HCRT1-8_001
560 HCRT2-6
560 HCRT2-6_001
560 NP136
560 NP136_001
560 Px_HA69
560 Px_HA69_001
560 Px_NP136
2400 Px_NP136_002
332 HA69
332 HCRT1-8
332 HCRT1-8_001
332 HCRT2-6
332 NP136
332 Px_HA69
332 Px_NP136
547 HA69
547 HCRT1-8
547 HCRT1-8_001
547 HCRT2-6
547 HCRT2-6_001
547 NP136
547 Px_HA69
547 Px_NP136
289 HCRT2-6
289 NP136
289 Px_HA69
289 Px_NP136
878 Px_HA69
878 Px_NP136
878 HA69
878 HCRT1-8

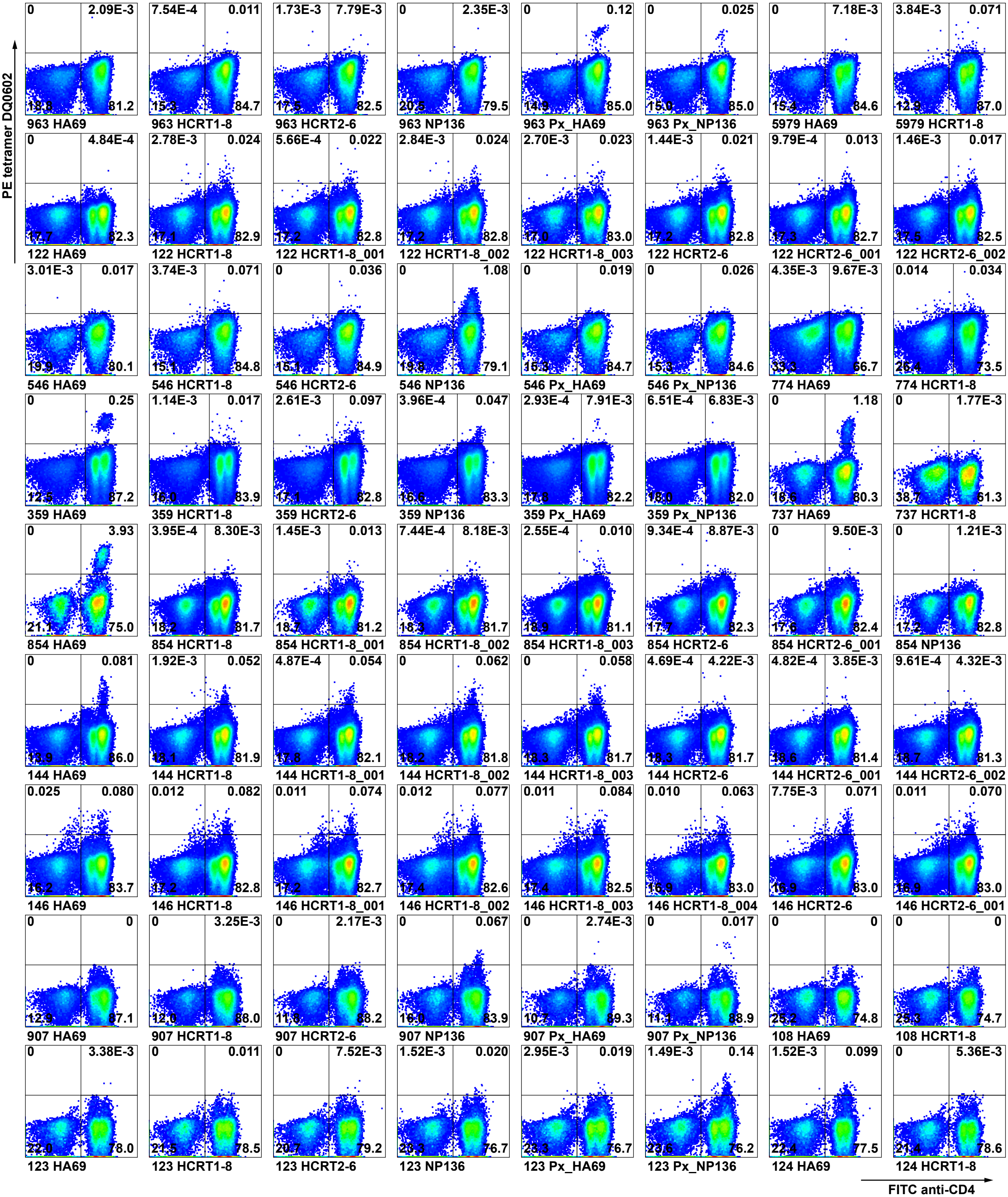
PE tetramer DQ0602
FITC anti-CD4
0 2.09E-3 18.8 81.2 963 HA69
7.54E-4 0.011 15.3 84.7 963 HCRT1-8
1.73E-3 7.79E-3 17.5 82.5 963 HCRT2-6
0 2.35E-3 20.5 79.5 963 NP136
0 0.12 14.9 85.0 963 Px_HA69
0 0.025 15.0 85.0 963 Px_NP136
0 7.18E-3 15.4 84.6 5979 HA69
3.84E-3 0.071 12.9 87.0 5979 HCRT1-8
0 4.84E-4 17.7 82.3 122 HA69
2.78E-3 0.024 17.1 82.9 122 HCRT1-8
5.66E-4 0.022 17.2 82.8 122 HCRT1-8_001
2.84E-3 0.024 17.2 82.8 122 HCRT1-8_002
2.70E-3 0.023 17.0 83.0 122 HCRT1-8_003
1.44E-3 0.021 17.2 82.8 122 HCRT2-6
9.79E-4 0.013 17.3 82.7 122 HCRT2-6_001
1.46E-3 0.017 17.5 82.5 122 HCRT2-6_002
3.01E-3 0.017 19.9 80.1 546 HA69
3.74E-3 0.071 15.1 84.8 546 HCRT1-8
0 0.036 15.1 84.9 546 HCRT2-6
0 1.08 19.8 79.1 546 NP136
0 0.019 15.3 84.7 546 Px_HA69
0 0.026 15.3 84.6 546 Px_NP136
4.35E-3 9.67E-3 33.3 66.7 774 HA69
0.014 0.034 26.4 73.5 774 HCRT1-8
0 0.25 12.5 87.2 359 HA69
1.14E-3 0.017 16.0 83.9 359 HCRT1-8
2.61E-3 0.097 17.1 82.8 359 HCRT2-6
3.96E-4 0.047 16.6 83.3 359 NP136
2.93E-4 7.91E-3 17.8 82.2 359 Px_HA69
6.51E-4 6.83E-3 18.0 82.0 359 Px_NP136
0 1.18 18.6 80.3 737 HA69
0 1.77E-3 38.7 61.3 737 HCRT1-8
0 3.93 21.1 75.0 854 HA69
3.95E-4 8.30E-3 18.2 81.7 854 HCRT1-8
1.45E-3 0.013 18.7 81.2 854 HCRT1-8_001
7.44E-4 8.18E-3 18.3 81.7 854 HCRT1-8_002
2.55E-4 0.010 18.9 81.1 854 HCRT1-8_003
9.34E-4 8.87E-3 17.7 82.3 854 HCRT2-6
0 9.50E-3 17.6 82.4 854 HCRT2-6_001
0 1.21E-3 17.2 82.8 854 NP136
0 0.081 13.9 86.0 144 HA69
1.92E-3 0.052 18.1 81.9 144 HCRT1-8
4.87E-4 0.054 17.8 82.1 144 HCRT1-8_001
0 0.062 18.2 81.8 144 HCRT1-8_002
0 0.058 18.3 81.7 144 HCRT1-8_003
4.69E-4 4.22E-3 18.3 81.7 144 HCRT2-6
4.82E-4 3.85E-3 18.6 81.4 144 HCRT2-6_001
9.61E-4 4.32E-3 18.7 81.3 144 HCRT2-6_002
0.025 0.080 16.2 83.7 146 HA69
0.012 0.082 17.2 82.8 146 HCRT1-8
0.011 0.074 17.2 82.7 146 HCRT1-8_001
0.012 0.077 17.4 82.6 146 HCRT1-8_002
0.011 0.084 17.4 82.5 146 HCRT1-8_003
0.010 0.063 16.9 83.0 146 HCRT1-8_004
7.75E-3 0.071 16.9 83.0 146 HCRT2-6
0.011 0.070 16.9 83.0 146 HCRT2-6_001
0 0 12.9 87.1 907 HA69
0 3.25E-3 12.0 88.0 907 HCRT1-8
0 2.17E-3 11.8 88.2 907 HCRT2-6
0 0.067 16.0 83.9 907 NP136
0 2.74E-3 10.7 89.3 907 Px_HA69
0 0.017 11.1 88.9 907 Px_NP136
0 0 25.2 74.8 108 HA69
0 0 25.3 74.7 108 HCRT1-8
0 3.38E-3 22.0 78.0 123 HA69
0 0.011 21.5 78.5 123 HCRT1-8
0 7.52E-3 20.7 79.2 123 HCRT2-6
1.52E-3 0.020 23.3 76.7 123 NP136
2.95E-3 0.019 23.3 76.7 123 Px_HA69
1.49E-3 0.14 23.6 76.2 123 Px_NP136
1.52E-3 0.099 22.4 77.5 124 HA69
0 5.36E-3 21.4 78.6 124 HCRT1-8

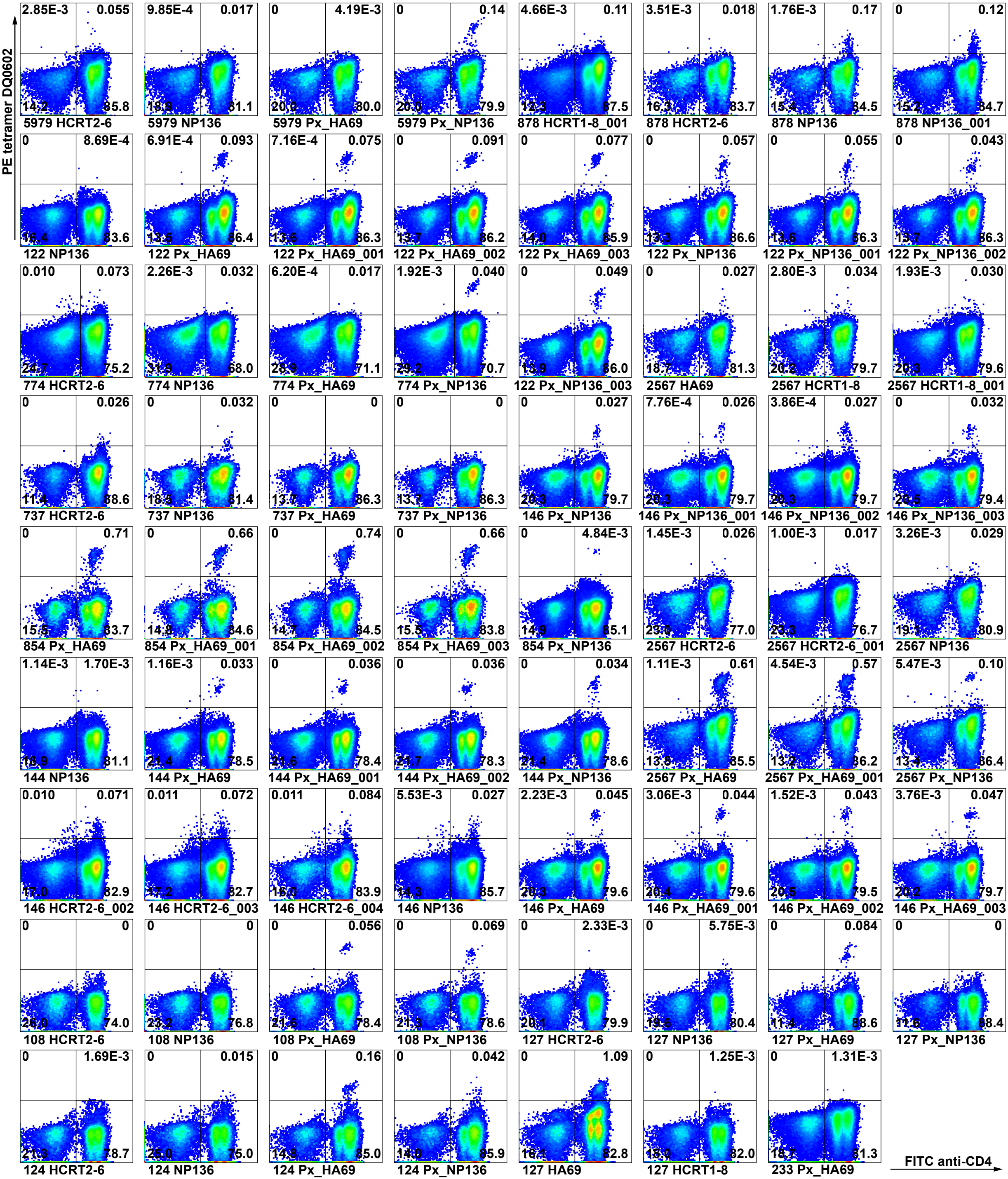
PE tetramer DQ0602
FITC anti-CD4
5979 HCRT2-6
5979 NP136
5979 Px_HA69
5979 Px_NP136
878 HCRT1-8_001
878 HCRT2-6
878 NP136
878 NP136_001
122 NP136
122 Px_HA69
122 Px_HA69_001
122 Px_HA69_002
122 Px_HA69_003
122 Px_NP136
122 Px_NP136_001
122 Px_NP136_002
774 HCRT2-6
774 NP136
774 Px_HA69
774 Px_NP136
122 Px_NP136_003
2567 HA69
2567 HCRT1-8
2567 HCRT1-8_001
737 HCRT2-6
737 NP136
737 Px_HA69
737 Px_NP136
146 Px_NP136
146 Px_NP136_001
146 Px_NP136_002
146 Px_NP136_003
854 Px_HA69
854 Px_HA69_001
854 Px_HA69_002
854 Px_HA69_003
854 Px_NP136
2567 HCRT2-6
2567 HCRT2-6_001
2567 NP136
144 NP136
144 Px_HA69
144 Px_HA69_001
144 Px_HA69_002
144 Px_NP136
2567 Px_HA69
2567 Px_HA69_001
2567 Px_NP136
146 HCRT2-6_002
146 HCRT2-6_003
146 HCRT2-6_004
146 NP136
146 Px_HA69
146 Px_HA69_001
146 Px_HA69_002
146 Px_HA69_003
108 HCRT2-6
108 NP136
108 Px_HA69
108 Px_NP136
127 HCRT2-6
127 NP136
127 Px_HA69
127 Px_NP136
124 HCRT2-6
124 NP136
124 Px_HA69
124 Px_NP136
127 HA69
127 HCRT1-8
233 Px_HA69

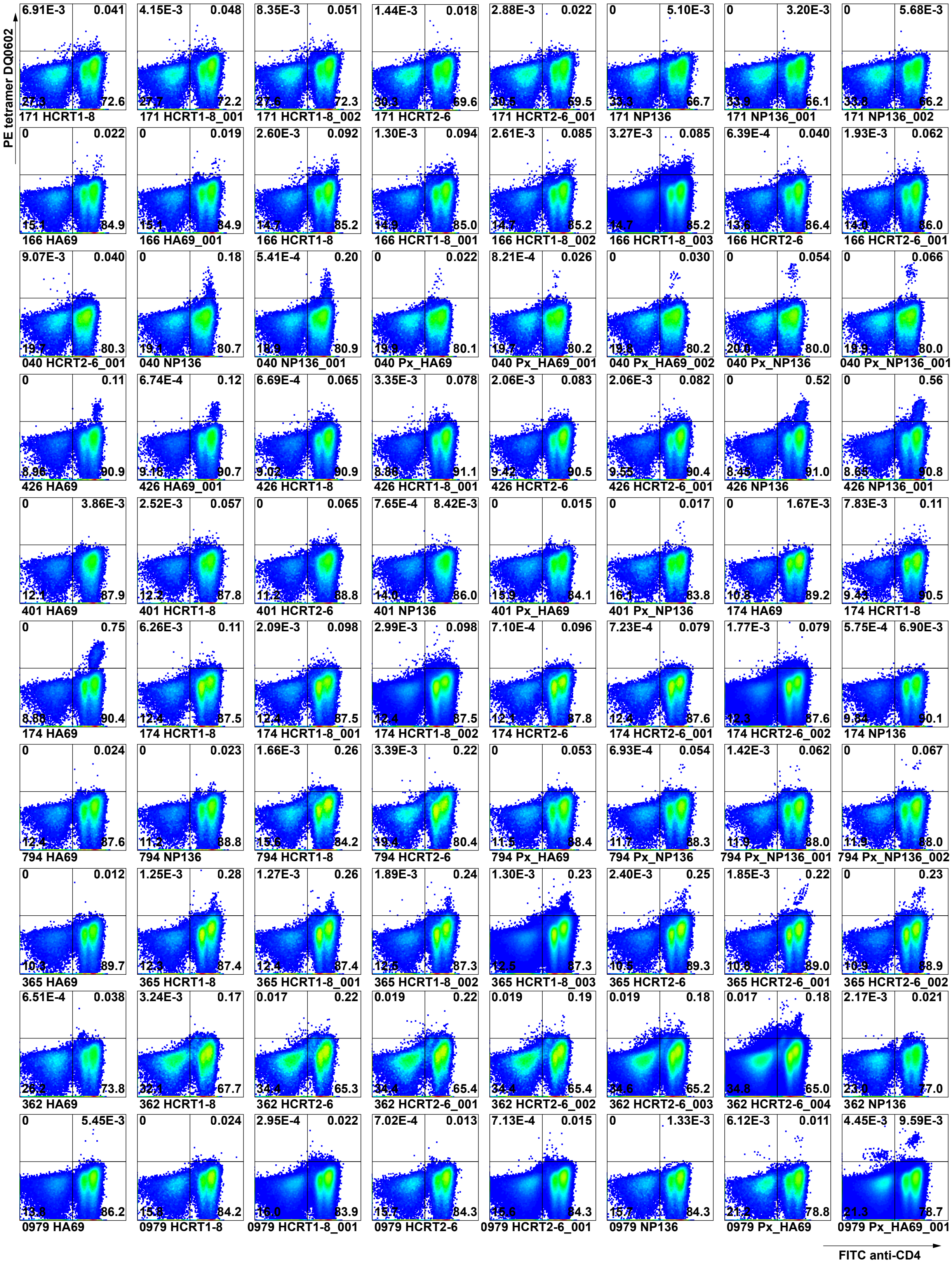
PE tetramer DQ0602
FITC anti-CD4
6.91E-3 0.041 27.3 72.6 171 HCRT1-8
4.15E-3 0.048 27.7 72.2 171 HCRT1-8_001
8.35E-3 0.051 27.6 72.3 171 HCRT1-8_002
1.44E-3 0.018 30.3 69.6 171 HCRT2-6
2.88E-3 0.022 30.5 69.5 171 HCRT2-6_001
0 5.10E-3 33.3 66.7 171 NP136
0 3.20E-3 33.9 66.1 171 NP136_001
0 5.68E-3 33.8 66.2 171 NP136_002
0 0.022 15.1 84.9 166 HA69
0 0.019 15.1 84.9 166 HA69_001
2.60E-3 0.092 14.7 85.2 166 HCRT1-8
1.30E-3 0.094 14.9 85.0 166 HCRT1-8_001
2.61E-3 0.085 14.7 85.2 166 HCRT1-8_002
3.27E-3 0.085 14.7 85.2 166 HCRT1-8_003
6.39E-4 0.040 13.6 86.4 166 HCRT2-6
1.93E-3 0.062 14.0 86.0 166 HCRT2-6_001
9.07E-3 0.040 19.7 80.3 040 HCRT2-6_001
0 0.18 19.1 80.7 040 NP136
5.41E-4 0.20 18.9 80.9 040 NP136_001
0 0.022 19.9 80.1 040 Px_HA69
8.21E-4 0.026 19.7 80.2 040 Px_HA69_001
0 0.030 19.8 80.2 040 Px_HA69_002
0 0.054 20.0 80.0 040 Px_NP136
0 0.066 19.9 80.0 040 Px_NP136_001
0 0.11 8.96 90.9 426 HA69
6.74E-4 0.12 9.16 90.7 426 HA69_001
6.69E-4 0.065 9.02 90.9 426 HCRT1-8
3.35E-3 0.078 8.88 91.1 426 HCRT1-8_001
2.06E-3 0.083 9.42 90.5 426 HCRT2-6
2.06E-3 0.082 9.55 90.4 426 HCRT2-6_001
0 0.52 8.45 91.0 426 NP136
0 0.56 8.65 90.8 426 NP136_001
0 3.86E-3 12.1 87.9 401 HA69
2.52E-3 0.057 12.2 87.8 401 HCRT1-8
0 0.065 11.2 88.8 401 HCRT2-6
7.65E-4 8.42E-3 14.0 86.0 401 NP136
0 0.015 15.9 84.1 401 Px_HA69
0 0.017 16.1 83.8 401 Px_NP136
0 1.67E-3 10.8 89.2 174 HA69
7.83E-3 0.11 9.43 90.5 174 HCRT1-8
0 0.75 8.88 90.4 174 HA69
6.26E-3 0.11 12.4 87.5 174 HCRT1-8
2.09E-3 0.098 12.4 87.5 174 HCRT1-8_001
2.99E-3 0.098 12.4 87.5 174 HCRT1-8_002
7.10E-4 0.096 12.1 87.8 174 HCRT2-6
7.23E-4 0.079 12.4 87.6 174 HCRT2-6_001
1.77E-3 0.079 12.3 87.6 174 HCRT2-6_002
5.75E-4 6.90E-3 9.84 90.1 174 NP136
0 0.024 12.4 87.6 794 HA69
0 0.023 11.2 88.8 794 NP136
1.66E-3 0.26 15.6 84.2 794 HCRT1-8
3.39E-3 0.22 19.4 80.4 794 HCRT2-6
0 0.053 11.5 88.4 794 Px_HA69
6.93E-4 0.054 11.7 88.3 794 Px_NP136
1.42E-3 0.062 11.9 88.0 794 Px_NP136_001
0 0.067 11.9 88.0 794 Px_NP136_002
0 0.012 10.3 89.7 365 HA69
1.25E-3 0.28 12.3 87.4 365 HCRT1-8
1.27E-3 0.26 12.4 87.4 365 HCRT1-8_001
1.89E-3 0.24 12.5 87.3 365 HCRT1-8_002
1.30E-3 0.23 12.5 87.3 365 HCRT1-8_003
2.40E-3 0.25 10.5 89.3 365 HCRT2-6
1.85E-3 0.22 10.8 89.0 365 HCRT2-6_001
0 0.23 10.9 88.9 365 HCRT2-6_002
6.51E-4 0.038 26.2 73.8 362 HA69
3.24E-3 0.17 32.1 67.7 362 HCRT1-8
0.017 0.22 34.4 65.3 362 HCRT2-6
0.019 0.22 34.4 65.4 362 HCRT2-6_001
0.019 0.19 34.4 65.4 362 HCRT2-6_002
0.019 0.18 34.6 65.2 362 HCRT2-6_003
0.017 0.18 34.8 65.0 362 HCRT2-6_004
2.17E-3 0.021 23.0 77.0 362 NP136
0 5.45E-3 13.8 86.2 0979 HA69
0 0.024 15.8 84.2 0979 HCRT1-8
2.95E-4 0.022 16.0 83.9 0979 HCRT1-8_001
7.02E-4 0.013 15.7 84.3 0979 HCRT2-6
7.13E-4 0.015 15.6 84.3 0979 HCRT2-6_001
0 1.33E-3 15.7 84.3 0979 NP136
6.12E-3 0.011 21.2 78.8 0979 Px_HA69
4.45E-3 9.59E-3 21.3 78.7 0979 Px_HA69_001

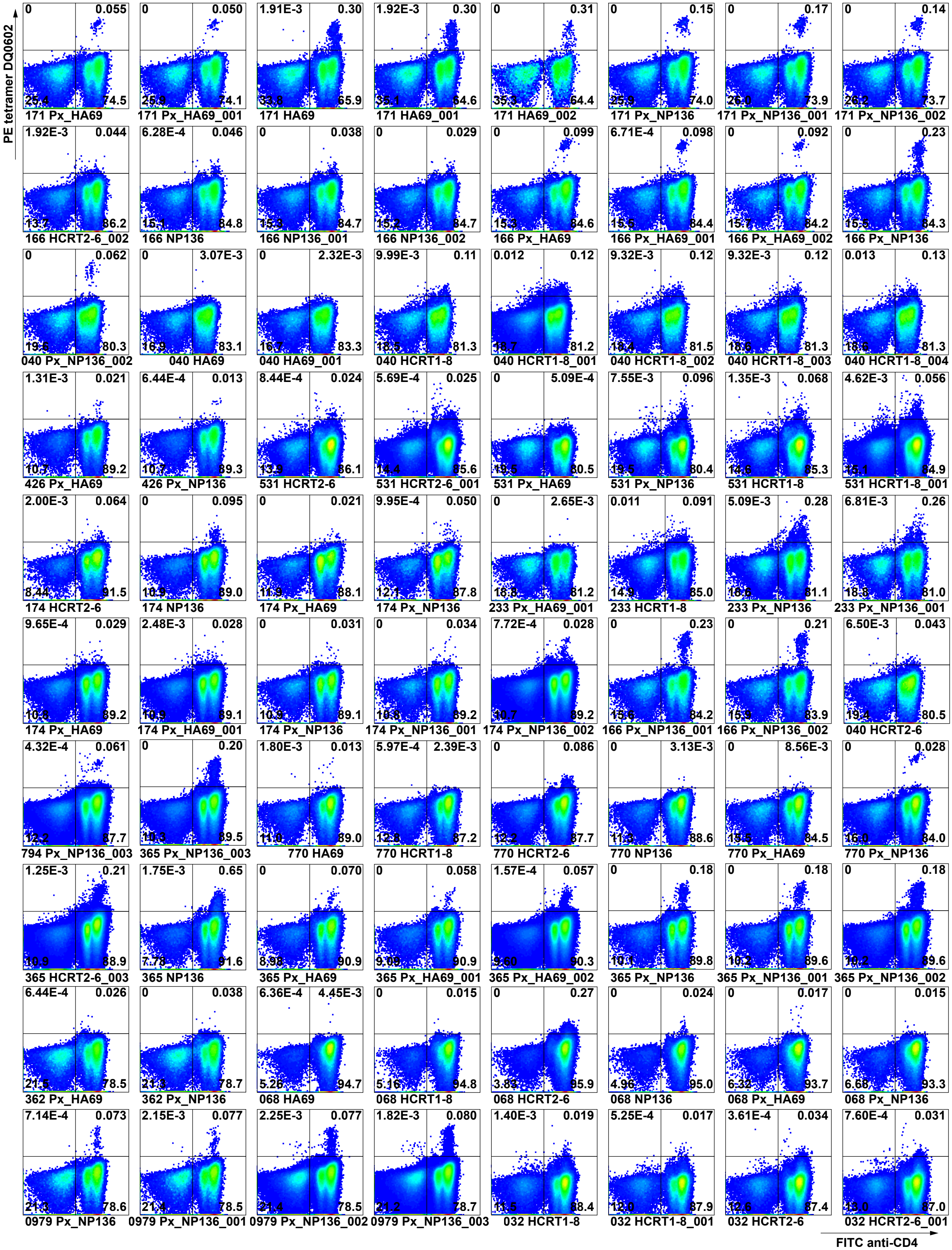
PE tetramer DQ0602
FITC anti-CD4
0 0.055 25.4 74.5 171 Px_HA69
0 0.050 25.9 74.1 171 Px_HA69_001
1.91E-3 0.30 33.8 65.9 171 HA69
1.92E-3 0.30 35.1 64.6 171 HA69_001
0 0.31 35.3 64.4 171 HA69_002
0 0.15 25.9 74.0 171 Px_NP136
0 0.17 26.0 73.9 171 Px_NP136_001
0 0.14 26.2 73.7 171 Px_NP136_002
1.92E-3 0.044 13.7 86.2 166 HCRT2-6_002
6.28E-4 0.046 15.1 84.8 166 NP136
0 0.038 15.3 84.7 166 NP136_001
0 0.029 15.2 84.7 166 NP136_002
0 0.099 15.3 84.6 166 Px_HA69
6.71E-4 0.098 15.5 84.4 166 Px_HA69_001
0 0.092 15.7 84.2 166 Px_HA69_002
0 0.23 15.6 84.3 166 Px_NP136
0 0.062 19.6 80.3 040 Px_NP136_002
0 3.07E-3 16.9 83.1 040 HA69
0 2.32E-3 16.7 83.3 040 HA69_001
9.99E-3 0.11 18.5 81.3 040 HCRT1-8
0.012 0.12 18.7 81.2 040 HCRT1-8_001
9.32E-3 0.12 18.4 81.5 040 HCRT1-8_002
9.32E-3 0.12 18.6 81.3 040 HCRT1-8_003
0.013 0.13 18.6 81.3 040 HCRT1-8_004
1.31E-3 0.021 10.7 89.2 426 Px_HA69
6.44E-4 0.013 10.7 89.3 426 Px_NP136
8.44E-4 0.024 13.9 86.1 531 HCRT2-6
5.69E-4 0.025 14.4 85.6 531 HCRT2-6_001
0 5.09E-4 19.5 80.5 531 Px_HA69
7.55E-3 0.096 19.5 80.4 531 Px_NP136
1.35E-3 0.068 14.6 85.3 531 HCRT1-8
4.62E-3 0.056 15.1 84.9 531 HCRT1-8_001
2.00E-3 0.064 8.44 91.5 174 HCRT2-6
0 0.095 10.9 89.0 174 NP136
0 0.021 11.9 88.1 174 Px_HA69
9.95E-4 0.050 12.1 87.8 174 Px_NP136
0 2.65E-3 18.8 81.2 233 Px_HA69_001
0.011 0.091 14.9 85.0 233 HCRT1-8
5.09E-3 0.28 18.6 81.1 233 Px_NP136
6.81E-3 0.26 18.8 81.0 233 Px_NP136_001
9.65E-4 0.029 10.8 89.2 174 Px_HA69
2.48E-3 0.028 10.9 89.1 174 Px_HA69_001
0 0.031 10.9 89.1 174 Px_NP136
0 0.034 10.8 89.2 174 Px_NP136_001
7.72E-4 0.028 10.7 89.2 174 Px_NP136_002
0 0.23 15.6 84.2 166 Px_NP136_001
0 0.21 15.9 83.9 166 Px_NP136_002
6.50E-3 0.043 19.4 80.5 040 HCRT2-6
4.32E-4 0.061 12.2 87.7 794 Px_NP136_003
0 0.20 10.3 89.5 365 Px_NP136_003
1.80E-3 0.013 11.0 89.0 770 HA69
5.97E-4 2.39E-3 12.8 87.2 770 HCRT1-8
0 0.086 12.2 87.7 770 HCRT2-6
0 3.13E-3 11.3 88.6 770 NP136
0 8.56E-3 15.5 84.5 770 Px_HA69
0 0.028 16.0 84.0 770 Px_NP136
1.25E-3 0.21 10.9 88.9 365 HCRT2-6_003
1.75E-3 0.65 7.78 91.6 365 NP136
0 0.070 8.98 90.9 365 Px_HA69
0 0.058 9.09 90.9 365 Px_HA69_001
1.57E-4 0.057 9.60 90.3 365 Px_HA69_002
0 0.18 10.1 89.8 365 Px_NP136
0 0.18 10.2 89.6 365 Px_NP136_001
0 0.18 10.2 89.6 365 Px_NP136_002
6.44E-4 0.026 21.5 78.5 362 Px_HA69
0 0.038 21.3 78.7 362 Px_NP136
6.36E-4 4.45E-3 5.26 94.7 068 HA69
0 0.015 5.16 94.8 068 HCRT1-8
0 0.27 3.83 95.9 068 HCRT2-6
0 0.024 4.96 95.0 068 NP136
0 0.017 6.32 93.7 068 Px_HA69
0 0.015 6.68 93.3 068 Px_NP136
7.14E-4 0.073 21.3 78.6 0979 Px_NP136
2.15E-3 0.077 21.4 78.5 0979 Px_NP136_001
2.25E-3 0.077 21.4 78.5 0979 Px_NP136_002
1.82E-3 0.080 21.2 78.7 0979 Px_NP136_003
1.40E-3 0.019 11.5 88.4 032 HCRT1-8
5.25E-4 0.017 12.0 87.9 032 HCRT1-8_001
3.61E-4 0.034 12.6 87.4 032 HCRT2-6
7.60E-4 0.031 13.0 87.0 032 HCRT2-6_001

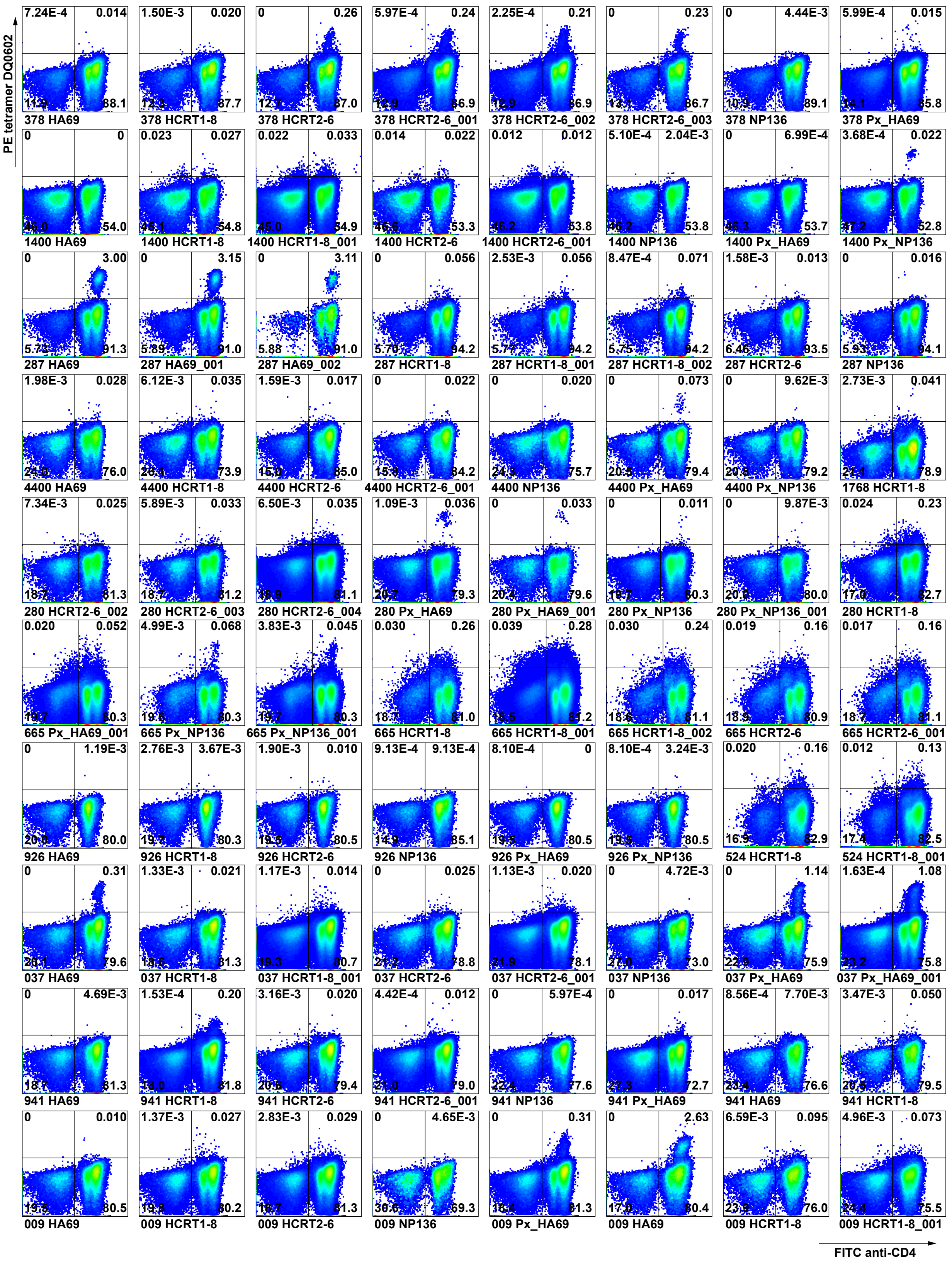

PE tetramer DQ0602
FITC anti-CD4
378 HA69
378 HCRT1-8
378 HCRT2-6
378 HCRT2-6_001
378 HCRT2-6_002
378 HCRT2-6_003
378 NP136
378 Px_HA69
1400 HA69
1400 HCRT1-8
1400 HCRT1-8_001
1400 HCRT2-6
1400 HCRT2-6_001
1400 NP136
1400 Px_HA69
1400 Px_NP136
287 HA69
287 HA69_001
287 HA69_002
287 HCRT1-8
287 HCRT1-8_001
287 HCRT1-8_002
287 HCRT2-6
287 NP136
4400 HA69
4400 HCRT1-8
4400 HCRT2-6
4400 HCRT2-6_001
4400 NP136
4400 Px_HA69
4400 Px_NP136
1768 HCRT1-8
280 HCRT2-6_002
280 HCRT2-6_003
280 HCRT2-6_004
280 Px_HA69
280 Px_HA69_001
280 Px_NP136
280 Px_NP136_001
280 HCRT1-8
665 Px_HA69_001
665 Px_NP136
665 Px_NP136_001
665 HCRT1-8
665 HCRT1-8_001
665 HCRT1-8_002
665 HCRT2-6
665 HCRT2-6_001
926 HA69
926 HCRT1-8
926 HCRT2-6
926 NP136
926 Px_HA69
926 Px_NP136
524 HCRT1-8
524 HCRT1-8_001
037 HA69
037 HCRT1-8
037 HCRT1-8_001
037 HCRT2-6
037 HCRT2-6_001
037 NP136
037 Px_HA69
037 Px_HA69_001
941 HA69
941 HCRT1-8
941 HCRT2-6
941 HCRT2-6_001
941 NP136
941 Px_HA69
941 HA69
941 HCRT1-8
009 HA69
009 HCRT1-8
009 HCRT2-6
009 NP136
009 Px_HA69
009 HA69
009 HCRT1-8
009 HCRT1-8_001

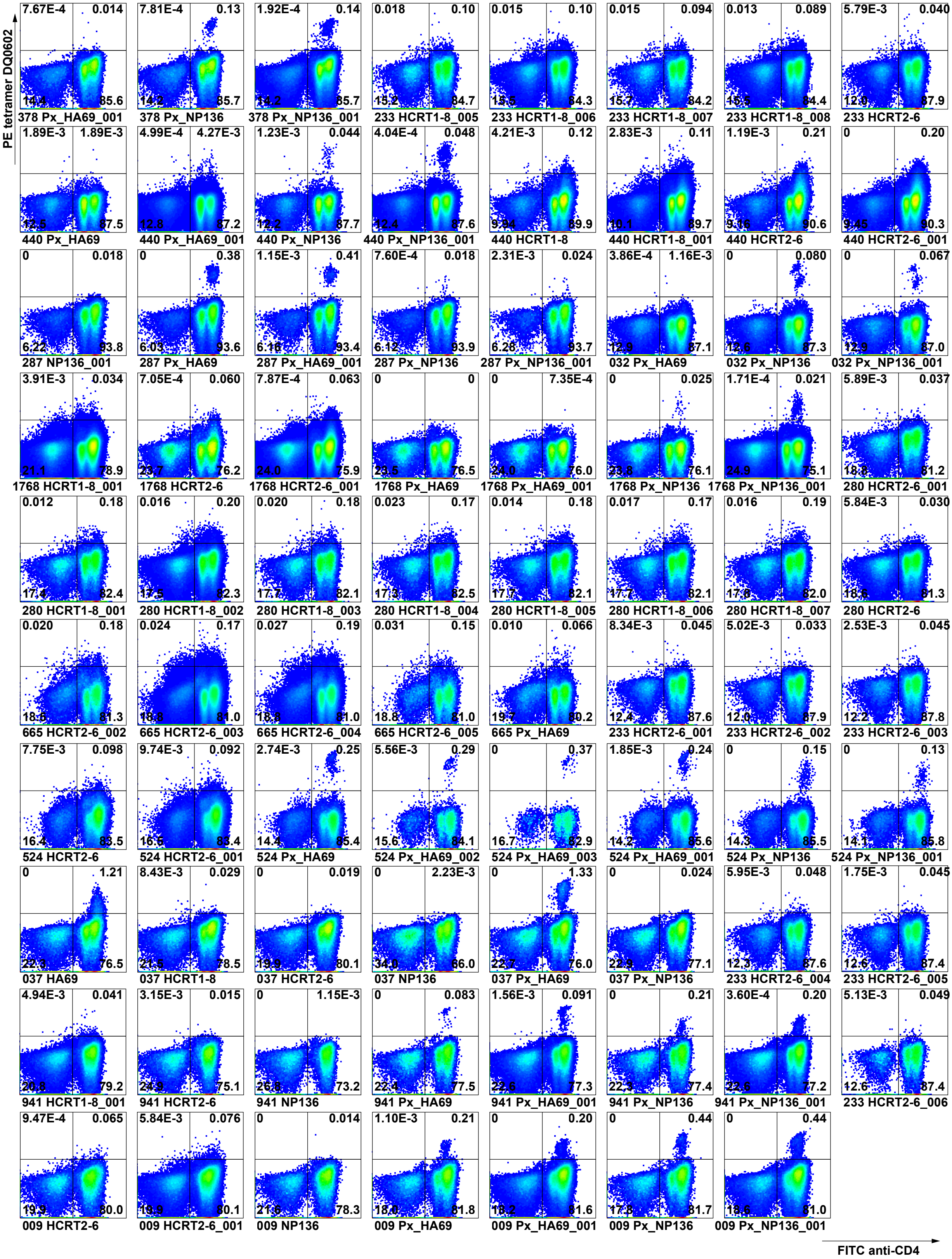
PE tetramer DQ0602
7.67E-4 0.014 14.4 85.6
378 Px_HA69_001
7.81E-4 0.13 14.2 85.7
378 Px_NP136
1.92E-4 0.14 14.2 85.7
378 Px_NP136_001
0.018 0.10 15.2 84.7
233 HCRT1-8_005
0.015 0.10 15.5 84.3
233 HCRT1-8_006
0.015 0.094 15.7 84.2
233 HCRT1-8_007
0.013 0.089 15.5 84.4
233 HCRT1-8_008
5.79E-3 0.040 12.0 87.9
233 HCRT2-6
1.89E-3 1.89E-3 12.5 87.5
440 Px_HA69
4.99E-4 4.27E-3 12.8 87.2
440 Px_HA69_001
1.23E-3 0.044 12.2 87.7
440 Px_NP136
4.04E-4 0.048 12.4 87.6
440 Px_NP136_001
4.21E-3 0.12 9.94 89.9
440 HCRT1-8
2.83E-3 0.11 10.1 89.7
440 HCRT1-8_001
1.19E-3 0.21 9.16 90.6
440 HCRT2-6
0 0.20 9.45 90.3
440 HCRT2-6_001
0 0.018 6.22 93.8
287 NP136_001
0 0.38 6.03 93.6
287 Px_HA69
1.15E-3 0.41 6.18 93.4
287 Px_HA69_001
7.60E-4 0.018 6.12 93.9
287 Px_NP136
2.31E-3 0.024 6.28 93.7
287 Px_NP136_001
3.86E-4 1.16E-3 12.9 87.1
032 Px_HA69
0 0.080 12.6 87.3
032 Px_NP136
0 0.067 12.9 87.0
032 Px_NP136_001
3.91E-3 0.034 21.1 78.9
1768 HCRT1-8_001
7.05E-4 0.060 23.7 76.2
1768 HCRT2-6
7.87E-4 0.063 24.0 75.9
1768 HCRT2-6_001
0 0 23.5 76.5
1768 Px_HA69
0 7.35E-4 24.0 76.0
1768 Px_HA69_001
0 0.025 23.8 76.1
1768 Px_NP136
1.71E-4 0.021 24.9 75.1
1768 Px_NP136_001
5.89E-3 0.037 18.8 81.2
280 HCRT2-6_001
0.012 0.18 17.4 82.4
280 HCRT1-8_001
0.016 0.20 17.5 82.3
280 HCRT1-8_002
0.020 0.18 17.7 82.1
280 HCRT1-8_003
0.023 0.17 17.3 82.5
280 HCRT1-8_004
0.014 0.18 17.7 82.1
280 HCRT1-8_005
0.017 0.17 17.7 82.1
280 HCRT1-8_006
0.016 0.19 17.8 82.0
280 HCRT1-8_007
5.84E-3 0.030 18.6 81.3
280 HCRT2-6
0.020 0.18 18.5 81.3
665 HCRT2-6_002
0.024 0.17 18.8 81.0
665 HCRT2-6_003
0.027 0.19 18.8 81.0
665 HCRT2-6_004
0.031 0.15 18.8 81.0
665 HCRT2-6_005
0.010 0.066 19.7 80.2
665 Px_HA69
8.34E-3 0.045 12.4 87.6
233 HCRT2-6_001
5.02E-3 0.033 12.0 87.9
233 HCRT2-6_002
2.53E-3 0.045 12.2 87.8
233 HCRT2-6_003
7.75E-3 0.098 16.4 83.5
524 HCRT2-6
9.74E-3 0.092 16.5 83.4
524 HCRT2-6_001
2.74E-3 0.25 14.4 85.4
524 Px_HA69
5.56E-3 0.29 15.6 84.1
524 Px_HA69_002
0 0.37 16.7 82.9
524 Px_HA69_003
1.85E-3 0.24 14.2 85.6
524 Px_HA69_001
0 0.15 14.3 85.5
524 Px_NP136
0 0.13 14.1 85.8
524 Px_NP136_001
0 1.21 22.3 76.5
037 HA69
8.43E-3 0.029 21.5 78.5
037 HCRT1-8
0 0.019 19.9 80.1
037 HCRT2-6
0 2.23E-3 34.0 66.0
037 NP136
0 1.33 22.7 76.0
037 Px_HA69
0 0.024 22.9 77.1
037 Px_NP136
5.95E-3 0.048 12.3 87.6
233 HCRT2-6_004
1.75E-3 0.045 12.6 87.4
233 HCRT2-6_005
4.94E-3 0.041 20.8 79.2
941 HCRT1-8_001
3.15E-3 0.015 24.9 75.1
941 HCRT2-6
0 1.15E-3 26.8 73.2
941 NP136
0 0.083 22.4 77.5
941 Px_HA69
1.56E-3 0.091 22.6 77.3
941 Px_HA69_001
0 0.21 22.3 77.4
941 Px_NP136
3.60E-4 0.20 22.6 77.2
941 Px_NP136_001
5.13E-3 0.049 12.6 87.4
233 HCRT2-6_006
9.47E-4 0.065 19.9 80.0
009 HCRT2-6
5.84E-3 0.076 19.9 80.1
009 HCRT2-6_001
0 0.014 21.6 78.3
009 NP136
1.10E-3 0.21 18.0 81.8
009 Px_HA69
0 0.20 18.2 81.6
009 Px_HA69_001
0 0.44 17.8 81.7
009 Px_NP136
0 0.44 18.6 81.0
009 Px_NP136_001
FITC anti-CD4

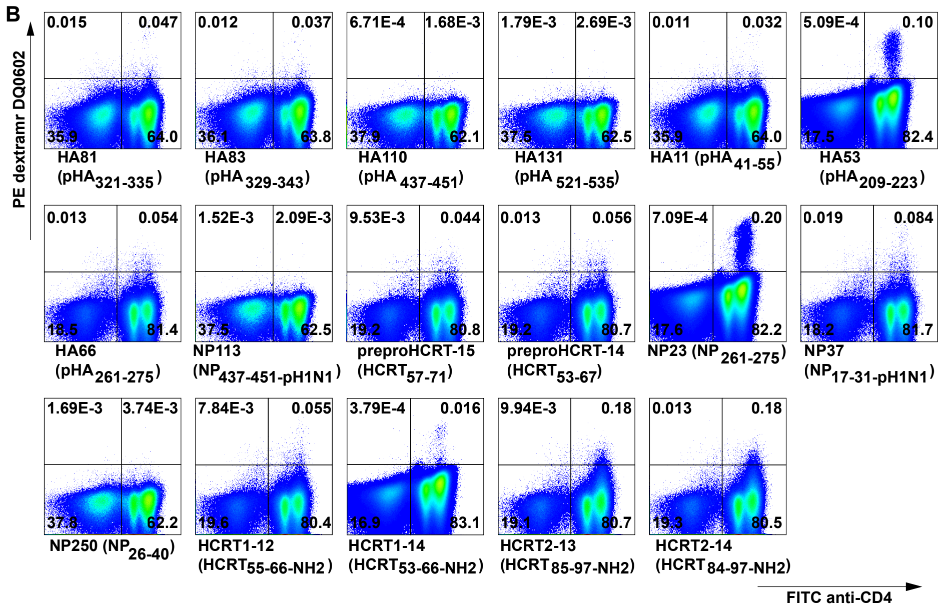
B
PE dextramr DQ0602
FITC anti-CD4
0.015 0.047 35.9 64.0
HA81 (pHA321-335)
0.012 0.037 36.1 63.8
HA83 (pHA329-343)
6.71E-4 1.68E-3 37.9 62.1
HA110 (pHA437-451)
1.79E-3 2.69E-3 37.5 62.5
HA131 (pHA521-535)
0.011 0.032 35.9 64.0
HA11 (pHA41-55)
5.09E-4 0.10 17.5 82.4
HA53 (pHA209-223)
0.013 0.054 18.5 81.4
HA66 (pHA261-275)
1.52E-3 2.09E-3 37.5 62.5
NP113 (NP437-451-pH1N1)
9.53E-3 0.044 19.2 80.8
preproHCRT-15 (HCRT57-71)
0.013 0.056 19.2 80.7
preproHCRT-14 (HCRT53-67)
7.09E-4 0.20 17.6 82.2
NP23 (NP261-275)
0.019 0.084 18.2 81.7
NP37 (NP17-31-pH1N1)
1.69E-3 3.74E-3 37.8 62.2
NP250 (NP26-40)
7.84E-3 0.055 19.6 80.4
HCRT1-12 (HCRT55-66-NH2)
3.79E-4 0.016 16.9 83.1
HCRT1-14 (HCRT53-66-NH2)
9.94E-3 0.18 19.1 80.7
HCRT2-13 (HCRT85-97-NH2)
0.013 0.18 19.3 80.5
HCRT2-14 (HCRT84-97-NH2)

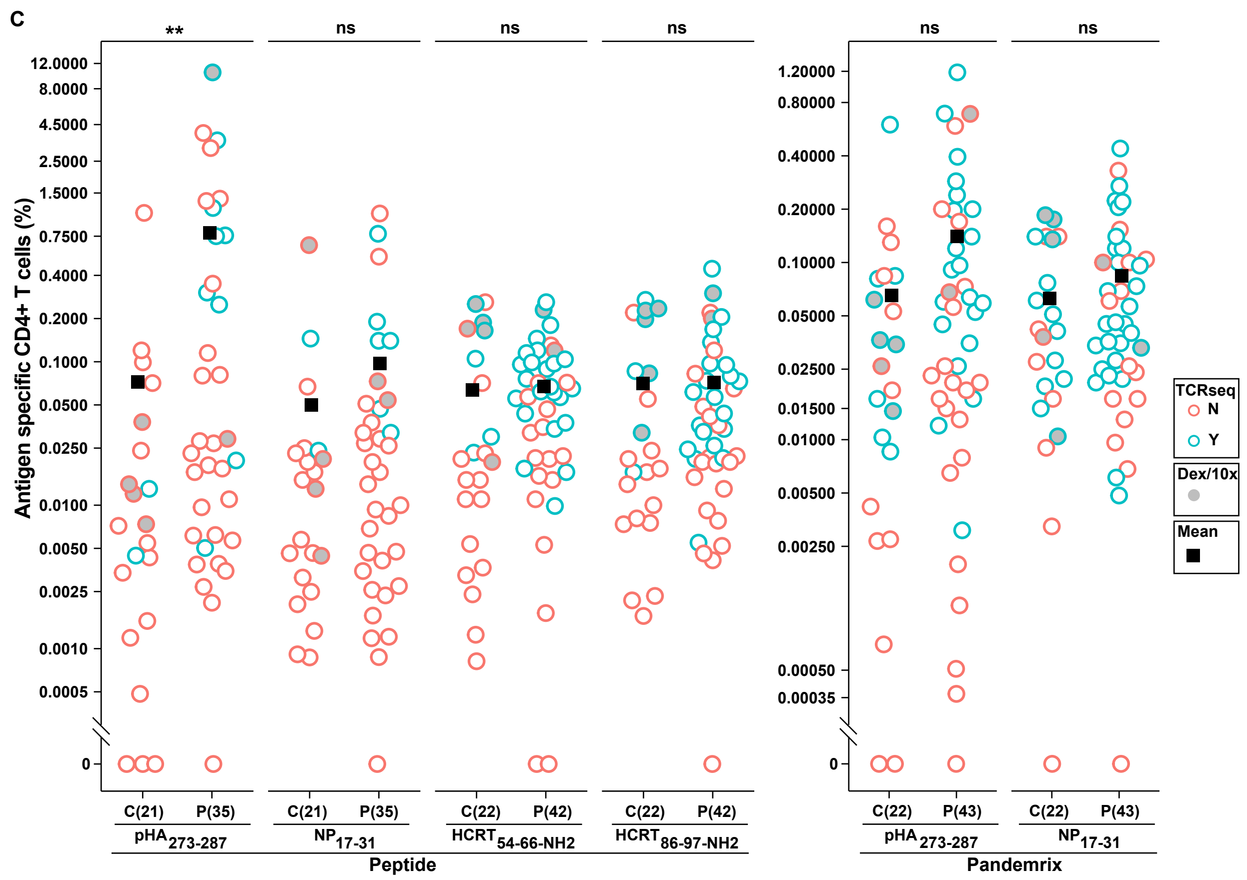
C
**
ns
ns
ns
ns
ns
Antigen specific CD4+ T cells (%)
12.0000
8.0000
4.5000
2.5000
1.5000
0.7500
0.4000
0.2000
0.1000
0.0500
0.0250
0.0100
0.0050
0.0025
0.0010
0.0005
0
1.20000
0.80000
0.40000
0.20000
0.10000
0.05000
0.02500
0.01500
0.01000
0.00500
0.00250
0.00050
0.00035
0
C(21) P(35)
pHA273-287
C(21) P(35)
NP17-31
C(22) P(42)
HCRT54-66-NH2
C(22) P(42)
HCRT86-97-NH2
Peptide
C(22) P(43)
pHA273-287
C(22) P(43)
NP17-31
Pandemrix
TCRseq
N
Y
Dex/10x
Mean

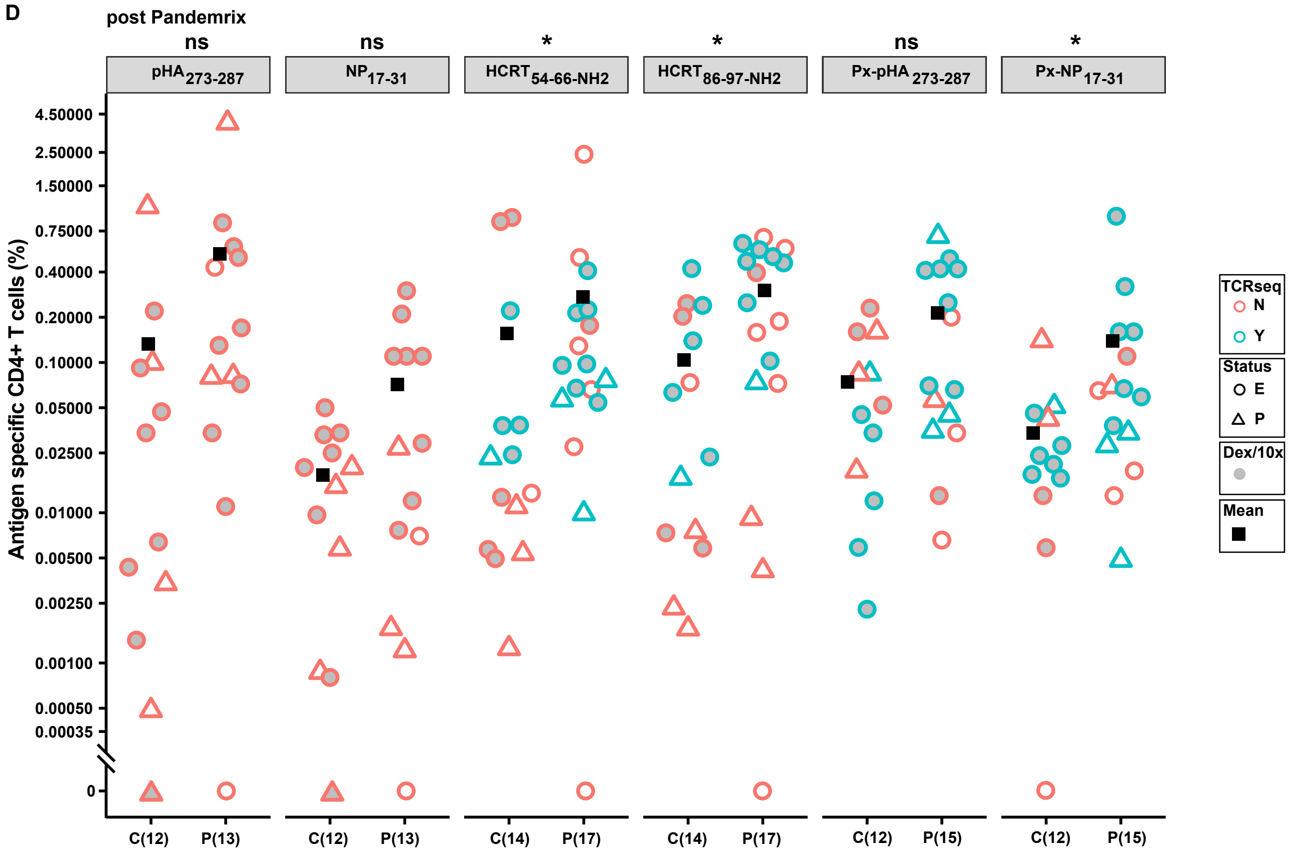
D
post Pandemrix
ns
ns
*
*
ns
*
pHA273-287
NP17-31
HCRT54-66-NH2
HCRT86-97-NH2
Px-pHA 273-287
Px-NP17-31
Antigen specific CD4+ T cells (%)
4.50000
2.50000
1.50000
0.75000
0.40000
0.20000
0.10000
0.05000
0.02500
0.01000
0.00500
0.00250
0.00100
0.00050
0.00035
0
C(12)
P(13)
C(12)
P(13)
C(14)
P(17)
C(14)
P(17)
C(12)
P(15)
C(12)
P(15)
TCRseq
N
Y
Status
E
P
Dex/10x
Mean

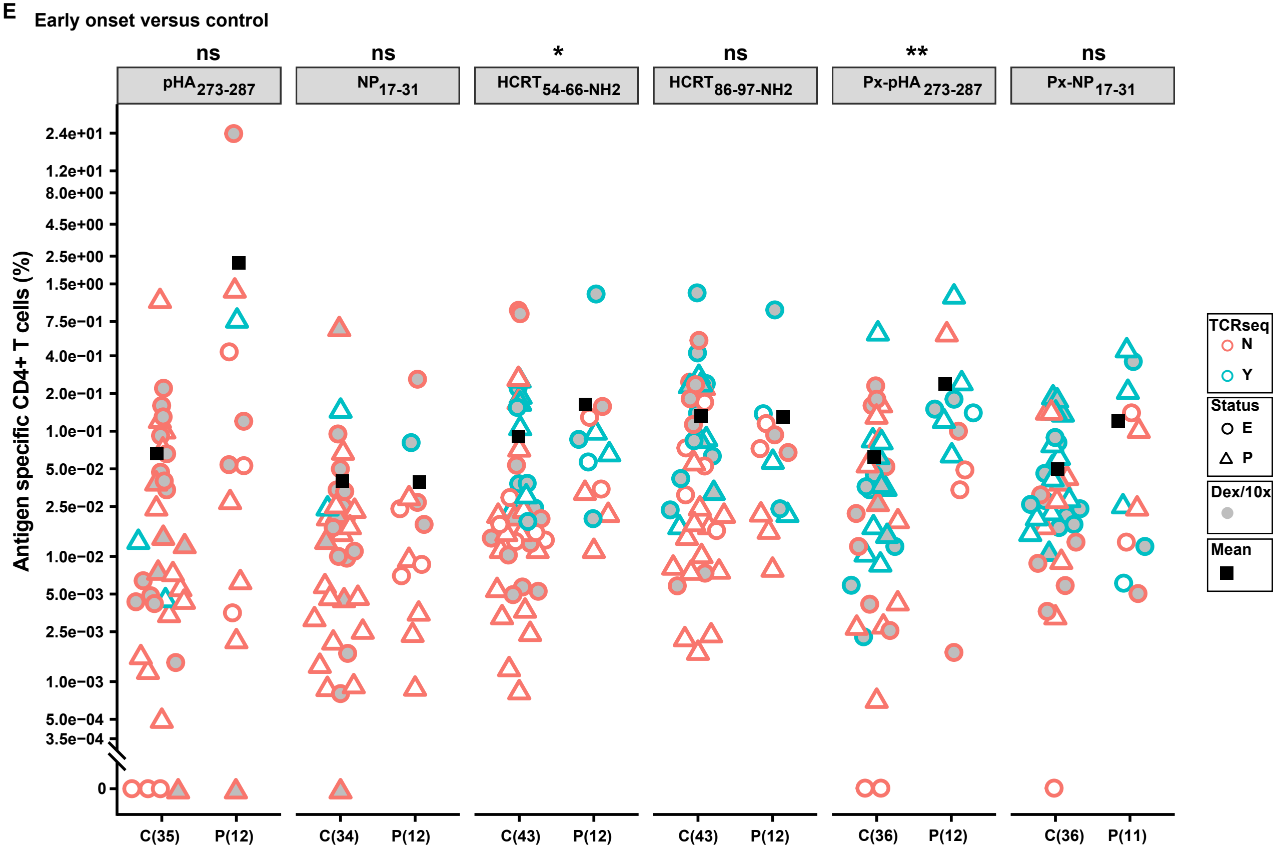
E
Early onset versus control
ns
ns
*
ns
**
ns
pHA273-287
NP17-31
HCRT54-66-NH2
HCRT86-97-NH2
Px-pHA 273-287
Px-NP17-31
Antigen specific CD4+ T cells (%)
2.4e+01
1.2e+01
8.0e+00
4.5e+00
2.5e+00
1.5e+00
7.5e−01
4.0e−01
2.0e−01
1.0e−01
5.0e−02
2.5e−02
1.0e−02
5.0e−03
2.5e−03
1.0e−03
5.0e−04
3.5e−04
0
C(35)
P(12)
C(34)
P(12)
C(43)
P(12)
C(43)
P(12)
C(36)
P(12)
C(36)
P(11)
TCRseq
N
Y
Status
E
P
Dex/10x
Mean

Fig. S1. Fluorescence activated cell sorting (FACS) plots of DQ0602 tetramer and dextramer staining. (A) Individual PBMCs were stimulated by Pandemrix® (Px) and stained by HA69 (Px_HA69) or NP136 (Px_NP136), or stimulated and tetramer stained by cognate peptide of (HA69, NP136, HCRT1-8 and HCRT2-6). More than one plots were recorded for some subjects. HA69, $pHA_{273\text{-}287}$; NP136, $NP_{17\text{-}31}$; HCRT1-8, $HCRT_{54\text{-}66\text{-}NH2}$; HCRT2-6, $HCRT_{86\text{-}97\text{-}NH2}$. (B) PBMCs stimulated by the same peptide were pooled and stained by cognate peptide DQ0602 dCODE® dextramer. Live CD3+ T cells are shown with frequency. For peptide and subject information, see Datasets S1and S2, respectively. (C) Frequency of antigen specific CD4+ T cells in extended subjects. PBMCs of 42 NT1 cases and 22 healthy controls were cultured individually with the cognate peptide (left panel) or Pandemrix® (right panel) and then stained with tetramer DQ0602 of $pHA_{273\text{-}287}$, $NP_{17\text{-}31}$, $HCRT_{54\text{-}66\text{-}NH2}$, and $HCRT_{86\text{-}97\text{-}NH2}$. Frequency of antigen specific CD4+ T cells in Pandemrix® vaccinated subjects (D) and in early onset versus healthy controls (E). PBMCs of NT1 cases and healthy controls were cultured individually with the cognate peptide or Pandemrix® and then stained with tetramer DQ0602 of $pHA_{273\text{-}287}$, $NP_{17\text{-}31}$, $HCRT_{54\text{-}66\text{-}NH2}$, and $HCRT_{86\text{-}97\text{-}NH2}$. Frequency was calculated in live CD3+ T cells. Each circle or triangle represents one subject. If multiple cultures or FACS recordings for one subject occurred, the mean of frequency was used. Subjects carried forward for single cell sorting and TCR sequencing are shown in blue. Subjects for dCODE® dextramer DQ0602 staining and 10x genomics sequencing are shown in grey (filled). Mean of frequency was shown in

black square. N, not sorted. Y, sorted and sequenced in 96-well plates. C, control. P, patient. *, $p < 0.05$; **, $p < 0.01$; ***, $p < 0.001$; ns, not significant.

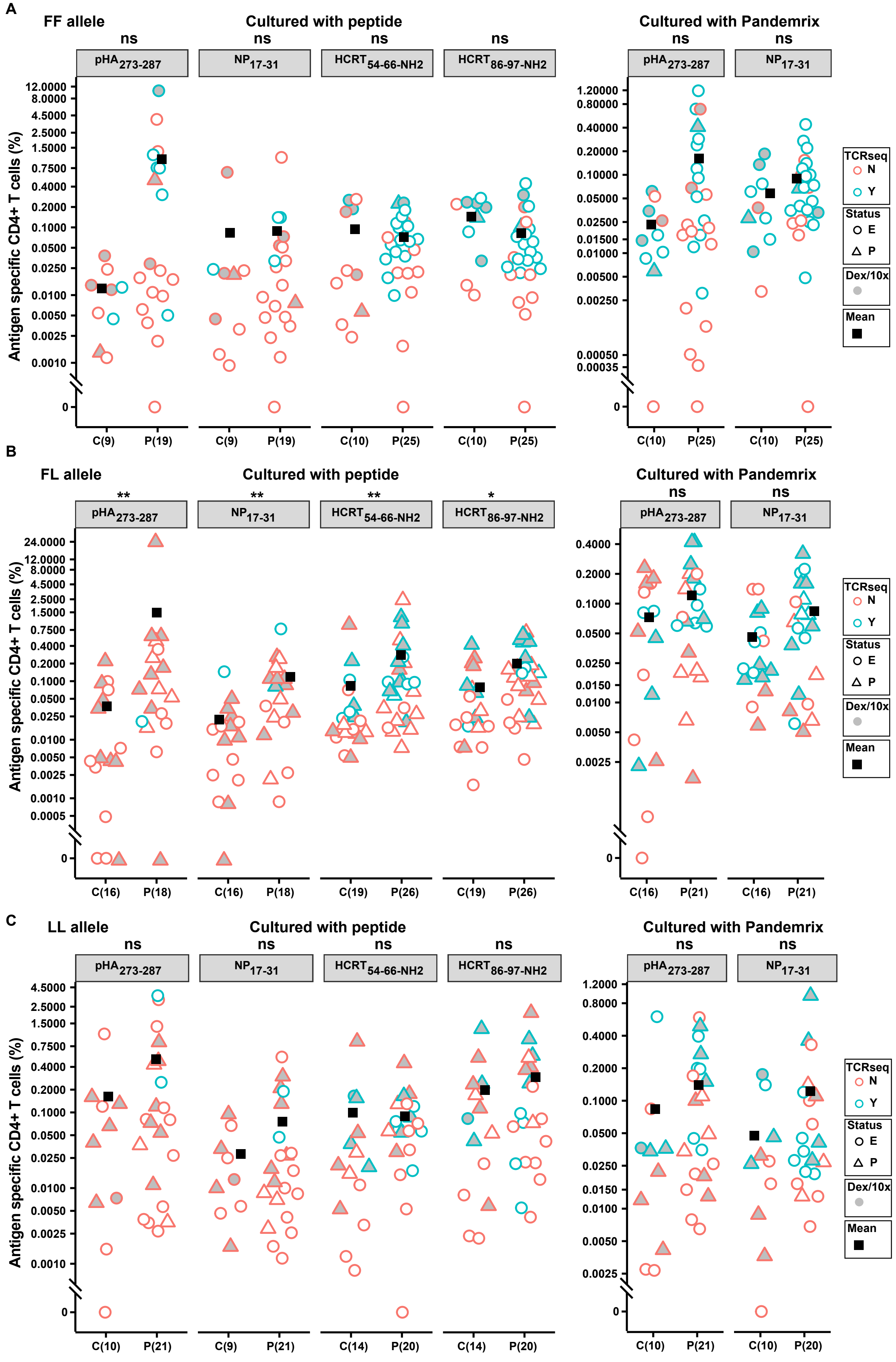
A
FF allele
Cultured with peptide
Cultured with Pandemrix
ns
pHA273-287
NP17-31
HCRT54-66-NH2
HCRT86-97-NH2
Antigen specific CD4+ T cells (%)
C(9)
P(19)
C(10)
P(25)
TCRseq
N
Y
Status
E
P
Dex/10x
Mean
B
FL allele
**
*
C(16)
P(18)
C(19)
P(26)
P(21)
C
LL allele
C(10)
P(21)
C(9)
C(14)
P(20)

Fig. S2. Frequency of antigen specific CD4+ T cells of TRAJ24 allele. PBMCs of 77 NT1 cases and 44 healthy controls (35 NT1 cases and 22 healthy controls of these were previously reported(1)) were cultured individually with cognate peptide or Pandemrix® and then stained with tetramer DQ0602 of $pHA_{273-287}$, NP_{17-31}, $HCRT_{54-66-NH2}$ and $HCRT_{86-97-NH2}$. Subjects were divided into three groups according to TRAJ24 genetic allele: FF (A), FL (B) and LL (C). Frequency was calculated in live CD3+ T cells. Each triangle or circle represents one subject, whereby subjects previously reported(1) are plotted as triangles and new data as circles. If multiple cultures or FACS recordings for one subject were completed, the mean of frequency was used. Subjects for single cell sorted and TCR sequenced are shown in blue and all other subjects in red. Subjects stained by dCODE® DQ0602 dextramer are shown in grey. For complete FACS plots, refer to Fig. S1. N, not sorted. Y, sorted and sequenced in 96-well plated. E, extended. P, previously reported(1). C, control. P, patient. *, $p < 0.05$. **, $p < 0.01$. ***, $p < 0.001$. ns, not significant.

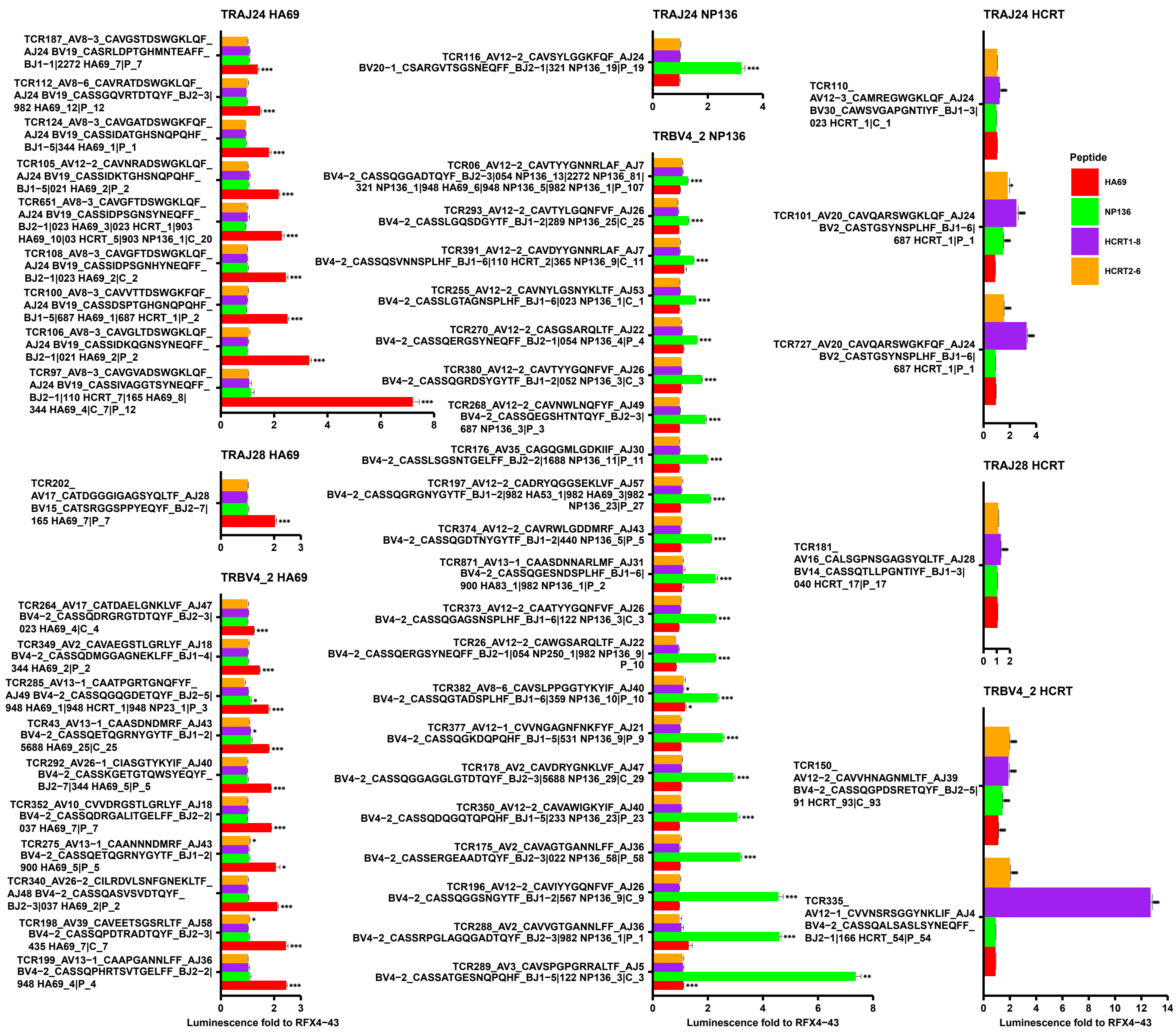
TRAJ24 HA69
TCR187_AV8−3_CAVGSTDSWGKLQF_AJ24 BV19_CASRLDPTGHMNTEAFF_BJ1−1|2272 HA69_7|P_7
TCR112_AV8−6_CAVRATDSWGKLQF_AJ24 BV19_CASSGQVRTDTQYF_BJ2−3|982 HA69_12|P_12
TCR124_AV8−3_CAVGATDSWGKFQF_AJ24 BV19_CASSIDATGHSNQPQHF_BJ1−5|344 HA69_1|P_1
TCR105_AV12−2_CAVNRADSWGKLQF_AJ24 BV19_CASSIDKTGHSNQPQHF_BJ1−5|021 HA69_2|P_2
TCR651_AV8−3_CAVGFTDSWGKLQF_AJ24 BV19_CASSIDPSGNSYNEQFF_BJ2−1|023 HA69_3|023 HCRT_1|903 HA69_10|03 HCRT_5|903 NP136_1|C_20
TCR108_AV8−3_CAVGFTDSWGKLQF_AJ24 BV19_CASSIDPSGNHYNEQFF_BJ2−1|023 HA69_2|C_2
TCR100_AV8−3_CAVVTTDSWGKFQF_AJ24 BV19_CASSDSPTGHGNQPQHF_BJ1−5|687 HA69_1|687 HCRT_1|P_2
TCR106_AV8−3_CAVGLTDSWGKLQF_AJ24 BV19_CASSIDKQGNSYNEQFF_BJ2−1|021 HA69_2|P_2
TCR97_AV8−3_CAVGVADSWGKLQF_AJ24 BV19_CASSIVAGGTSYNEQFF_BJ2−1|110 HCRT_7|165 HA69_8|344 HA69_4|C_7|P_12
***
0 2 4 6 8
TRAJ28 HA69
TCR202_AV17_CATDGGGIGAGSYQLTF_AJ28 BV15_CATSRGGSPPYEQYF_BJ2−7|165 HA69_7|P_7
***
0 2 3
TRBV4_2 HA69
TCR264_AV17_CATDAELGNKLVF_AJ47 BV4−2_CASSQDRGRGTDTQYF_BJ2−3|023 HA69_4|C_4
TCR349_AV2_CAVAEGSTLGRLYF_AJ18 BV4−2_CASSQDMGGAGNEKLFF_BJ1−4|344 HA69_2|P_2
TCR285_AV13−1_CAATPGRTGNQFYF_AJ49 BV4−2_CASSQGQGDETQYF_BJ2−5|948 HA69_1|948 HCRT_1|948 NP23_1|P_3
TCR43_AV13−1_CAASDNDMRF_AJ43 BV4−2_CASSQETQGRNYGYTF_BJ1−2|5688 HA69_25|C_25
TCR292_AV26−1_CIASGTYKYIF_AJ40 BV4−2_CASSKGETGTQWSYEQYF_BJ2−7|344 HA69_5|P_5
TCR352_AV10_CVVDRGSTLGRLYF_AJ18 BV4−2_CASSQDRGALITGELFF_BJ2−2|037 HA69_7|P_7
TCR275_AV13−1_CAANNNDMRF_AJ43 BV4−2_CASSQETQGRNYGYTF_BJ1−2|900 HA69_5|P_5
TCR340_AV26−2_CILRDVLSNFGNEKLTF_AJ48 BV4−2_CASSQASVSVDTQYF_BJ2−3|037 HA69_2|P_2
TCR198_AV39_CAVEETSGSRLTF_AJ58 BV4−2_CASSQPDTRADTQYF_BJ2−3|435 HA69_7|C_7
TCR199_AV13−1_CAAPGANNLFF_AJ36 BV4−2_CASSQPHRTSVTGELFF_BJ2−2|948 HA69_4|P_4
*** *
0 2 3
Luminescence fold to RFX4−43
TRAJ24 NP136
TCR116_AV12−2_CAVSYLGGKFQF_AJ24 BV20−1_CSARGVTSGSNEQFF_BJ2−1|321 NP136_19|P_19
***
0 2 4
TRBV4_2 NP136
TCR06_AV12−2_CAVTYYGNNRLAF_AJ7 BV4−2_CASSQGGADTQYF_BJ2−3|054 NP136_13|2272 NP136_81|321 NP136_1|948 HA69_6|948 NP136_5|982 NP136_1|P_107
TCR293_AV12−2_CAVTYLGQNFVF_AJ26 BV4−2_CASSLGQSDGYTF_BJ1−2|289 NP136_25|C_25
TCR391_AV12−2_CAVDYYGNNRLAF_AJ7 BV4−2_CASSQSVNNSPLHF_BJ1−6|110 HCRT_2|365 NP136_9|C_11
TCR255_AV12−2_CAVNYLGSNYKLTF_AJ53 BV4−2_CASSLGTAGNSPLHF_BJ1−6|023 NP136_1|C_1
TCR270_AV12−2_CASGSARQLTF_AJ22 BV4−2_CASSQERGSYNEQFF_BJ2−1|054 NP136_4|P_4
TCR380_AV12−2_CAVTYYGQNFVF_AJ26 BV4−2_CASSQGRDSYGYTF_BJ1−2|052 NP136_3|C_3
TCR268_AV12−2_CAVNWLNQFYF_AJ49 BV4−2_CASSQEGSHTNTQYF_BJ2−3|687 NP136_3|P_3
TCR176_AV35_CAGQGMLGDKIIF_AJ30 BV4−2_CASSLSGSNTGELFF_BJ2−2|1688 NP136_11|P_11
TCR197_AV12−2_CADRYQGGSEKLVF_AJ57 BV4−2_CASSQGRGNYGYTF_BJ1−2|982 HA53_1|982 HA69_3|982 NP136_23|P_27
TCR374_AV12−2_CAVRWLGDDMRF_AJ43 BV4−2_CASSQGDTNYGYTF_BJ1−2|440 NP136_5|P_5
TCR871_AV13−1_CAASDNNARLMF_AJ31 BV4−2_CASSQGESNDSPLHF_BJ1−6|900 HA83_1|982 NP136_1|P_2
TCR373_AV12−2_CAATYYGQNFVF_AJ26 BV4−2_CASSQGAGSNSPLHF_BJ1−6|122 NP136_3|C_3
TCR26_AV12−2_CAWGSARQLTF_AJ22 BV4−2_CASSQERGSYNEQFF_BJ2−1|054 NP250_1|982 NP136_9|P_10
TCR382_AV8−6_CAVSLPPGGTYKYIF_AJ40 BV4−2_CASSQGTADSPLHF_BJ1−6|359 NP136_10|P_10
TCR377_AV12−1_CVVNGAGNFNKFYF_AJ21 BV4−2_CASSQGKDQPQHF_BJ1−5|531 NP136_9|P_9
TCR178_AV2_CAVDRYGNKLVF_AJ47 BV4−2_CASSQGGAGGLGTDTQYF_BJ2−3|5688 NP136_29|C_29
TCR350_AV12−2_CAVAWIGKYIF_AJ40 BV4−2_CASSQDQGQTQPQHF_BJ1−5|233 NP136_23|P_23
TCR175_AV2_CAVAGTGANNLFF_AJ36 BV4−2_CASSERGEAADTQYF_BJ2−3|022 NP136_58|P_58
TCR196_AV12−2_CAVIYYGQNFVF_AJ26 BV4−2_CASSQGGSNGYTF_BJ1−2|567 NP136_9|C_9
TCR288_AV2_CAVVGTGANNLFF_AJ36 BV4−2_CASSRPGLAGQGADTQYF_BJ2−3|982 NP136_1|P_1
TCR289_AV3_CAVSPGPGRRALTF_AJ5 BV4−2_CASSATGESNQPQHF_BJ1−5|122 NP136_3|C_3
*** ** *
0 2 4 6 8
Luminescence fold to RFX4−43
TRAJ24 HCRT
TCR110_AV12−3_CAMREGWGKLQF_AJ24 BV30_CAWSVGAPGNTIYF_BJ1−3|023 HCRT_1|C_1
TCR101_AV20_CAVQARSWGKLQF_AJ24 BV2_CASTGSYNSPLHF_BJ1−6|687 HCRT_1|P_1
TCR727_AV20_CAVQARSWGKFQF_AJ24 BV2_CASTGSYNSPLHF_BJ1−6|687 HCRT_1|P_1
0 2 4
TRAJ28 HCRT
TCR181_AV16_CALSGPNSGAGSYQLTF_AJ28 BV14_CASSQTLLPGNTIYF_BJ1−3|040 HCRT_17|P_17
0 1 2
TRBV4_2 HCRT
TCR150_AV12−2_CAVVHNAGNMLTF_AJ39 BV4−2_CASSQGPDSRETQYF_BJ2−5|91 HCRT_93|C_93
TCR335_AV12−1_CVVNSRSGGYNKLIF_AJ4 BV4−2_CASSQALSASLSYNEQFF_BJ2−1|166 HCRT_54|P_54
0 2 4 6 8 10 12 14
Luminescence fold to RFX4−43
Peptide
HA69
NP136
HCRT1-8
HCRT2-6

Fig. S3. Activated TRAJ24, TRBV4-2 and TRAJ28. TCRs were co-cultured with artificial antigen presenting cells (APC) and $pHA_{273\text{-}287}$, $NP_{17\text{-}31}$, $HCRT_{54\text{-}66\text{-}NH2}$ and $HCRT_{86\text{-}97\text{-}NH2}$ for 8 hours in triplicate (n = 3). Luciferase activity was measured. TCR was considered as activation by a peptide with >=1.2 fold change of luminescence comparing to RFX4-43 and p <0.05. TCRs were grouped by TR genes and activation peptide and ordered by fold change. Y-axis is labeled as follows: TCR number, TR genes, sequence of CDR3α and CDR3β, subject identity and peptide used to recover TCR followed by clone count, diagnosis of subject followed by clone count. C, control. P, patient. *, p <0.05. **, p <0.01. ***, p <0.001.

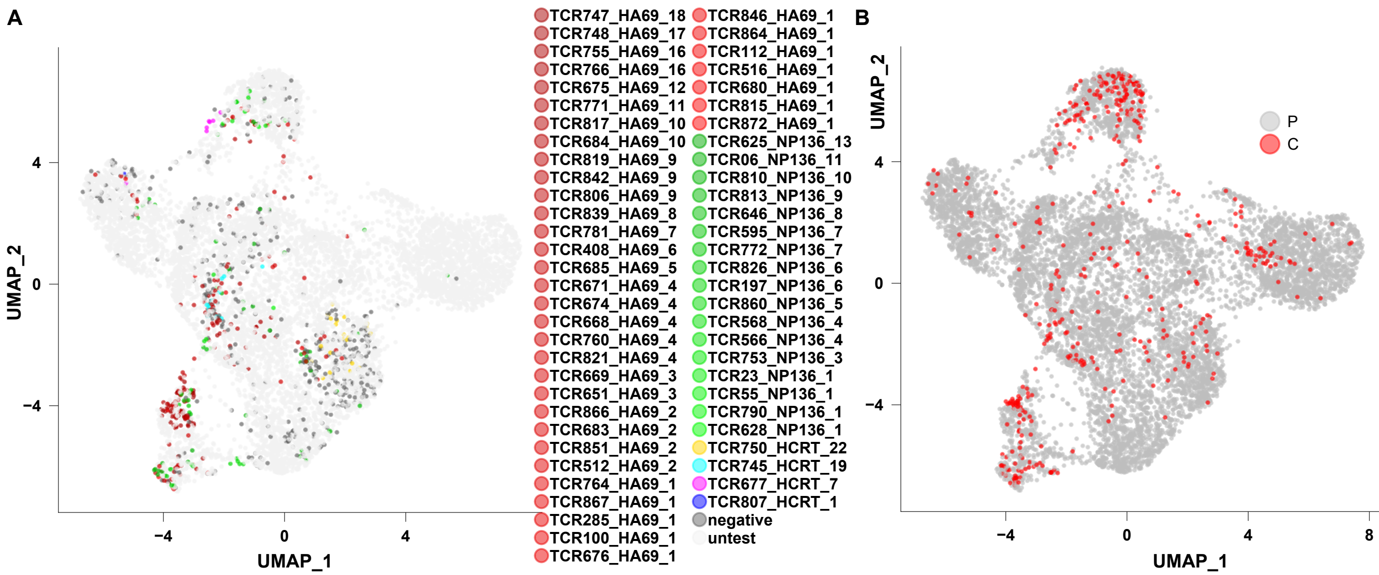

A
B
UMAP_2
UMAP_1
4
0
−4
8
TCR747_HA69_18
TCR748_HA69_17
TCR755_HA69_16
TCR766_HA69_16
TCR675_HA69_12
TCR771_HA69_11
TCR817_HA69_10
TCR684_HA69_10
TCR819_HA69_9
TCR842_HA69_9
TCR806_HA69_9
TCR839_HA69_8
TCR781_HA69_7
TCR408_HA69_6
TCR685_HA69_5
TCR671_HA69_4
TCR674_HA69_4
TCR668_HA69_4
TCR760_HA69_4
TCR821_HA69_4
TCR669_HA69_3
TCR651_HA69_3
TCR866_HA69_2
TCR683_HA69_2
TCR851_HA69_2
TCR512_HA69_2
TCR764_HA69_1
TCR867_HA69_1
TCR285_HA69_1
TCR100_HA69_1
TCR676_HA69_1
TCR846_HA69_1
TCR864_HA69_1
TCR112_HA69_1
TCR516_HA69_1
TCR680_HA69_1
TCR815_HA69_1
TCR872_HA69_1
TCR625_NP136_13
TCR06_NP136_11
TCR810_NP136_10
TCR813_NP136_9
TCR646_NP136_8
TCR595_NP136_7
TCR772_NP136_7
TCR826_NP136_6
TCR197_NP136_6
TCR860_NP136_5
TCR568_NP136_4
TCR566_NP136_4
TCR753_NP136_3
TCR23_NP136_1
TCR55_NP136_1
TCR790_NP136_1
TCR628_NP136_1
TCR750_HCRT_22
TCR745_HCRT_19
TCR677_HCRT_7
TCR807_HCRT_1
negative
untest
P
C

Fig. S4. UMAP of dextramer+CD4+ T cells. Gene expression of filtered single cells (see quality control in methods) were scaled and clustered using Seurat SCTransform. (A) Single cells containing activated TCR clones. Each TCR clone was followed by its reactive peptide and cell count in succession in legend. (B) Cells from NT1 cases and controls. C, control. P, patient.

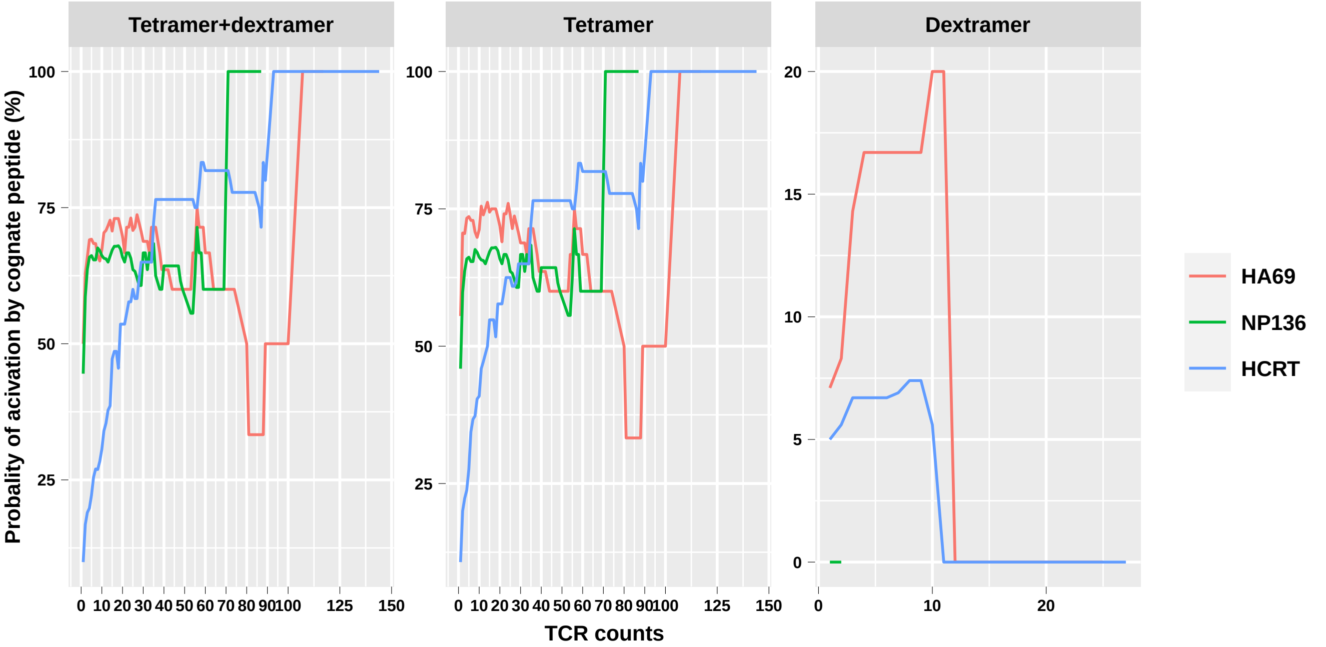

Tetramer+dextramer
Tetramer
Dextramer
Probality of acivation by cognate peptide (%)
TCR counts
HA69
NP136
HCRT
100
75
50
25
20
15
10
5
0
0 10 20 30 40 50 60 70 80 90 100 125 150
0 10 20

Fig. S5. Probability of TCR activation by cognate peptide versus clone count. Only TCRs recovered from a single peptide were used. The percentate was computed as number of TCRs activated by a peptide with >= a count divided by number of TCRs retrieved soly by that peptide with >= that count.

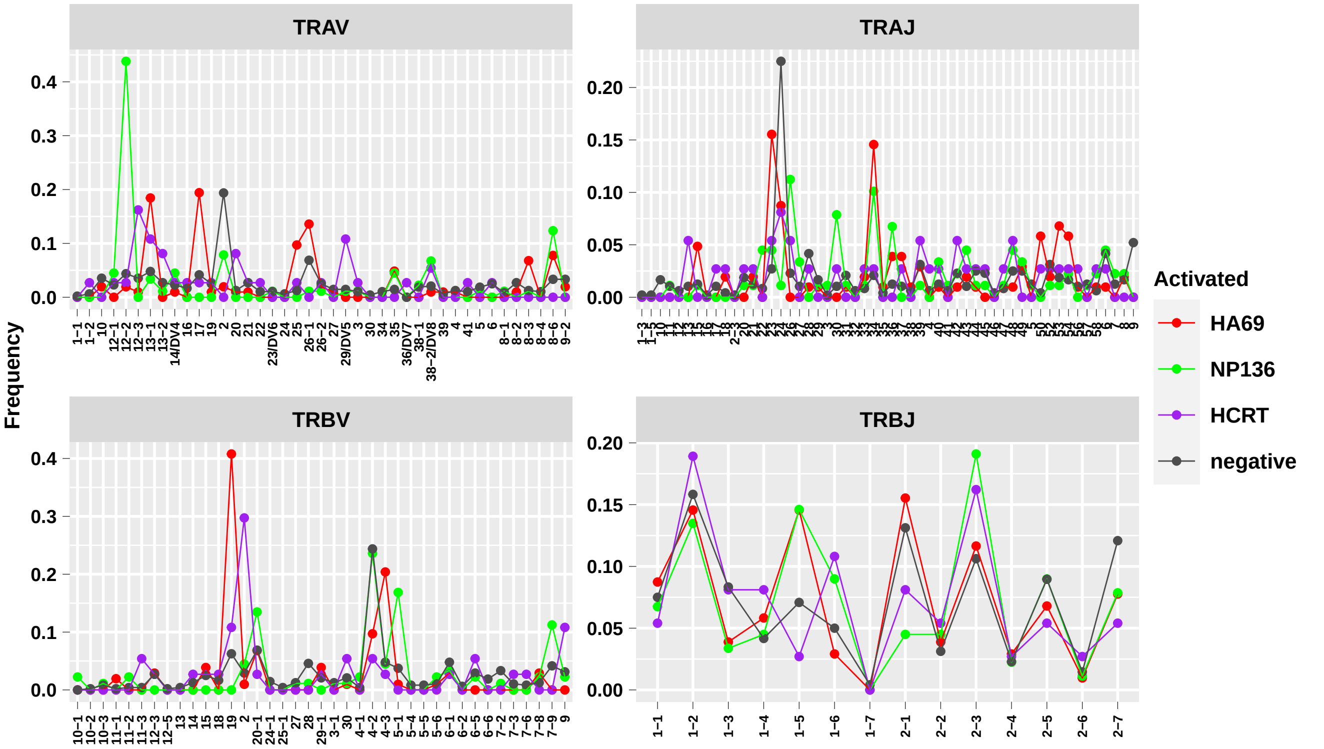
TRAV
TRAJ
TRBV
TRBJ
Frequency
Activated
HA69
NP136
HCRT
negative
0.0
0.1
0.2
0.3
0.4
0.00
0.05
0.10
0.15
0.20
1−1 1−2 10 12−1 12−2 12−3 13−1 13−2 14/DV4 16 17 19 2 20 21 22 23/DV6 24 25 26−1 26−2 27 29/DV5 3 30 34 35 36/DV7 38−1 38−2/DV8 39 4 41 5 6 8−1 8−2 8−3 8−4 8−6 9−2
10−1 10−2 10−3 11−1 11−2 11−3 12−3 12−5 13 14 15 18 19 2 20−1 24−1 25−1 27 28 29−1 3−1 30 4−1 4−2 4−3 5−1 5−4 5−5 5−6 6−1 6−2 6−5 6−6 7−2 7−3 7−6 7−8 7−9 9
1−1 1−2 1−3 1−4 1−5 1−6 1−7 2−1 2−2 2−3 2−4 2−5 2−6 2−7

Fig. S6. Frequency of TR gene usage by activation peptides $pHA_{273\text{-}287}$, $NP_{17\text{-}31}$, $HCRT_{NH2}$ and negative clones.

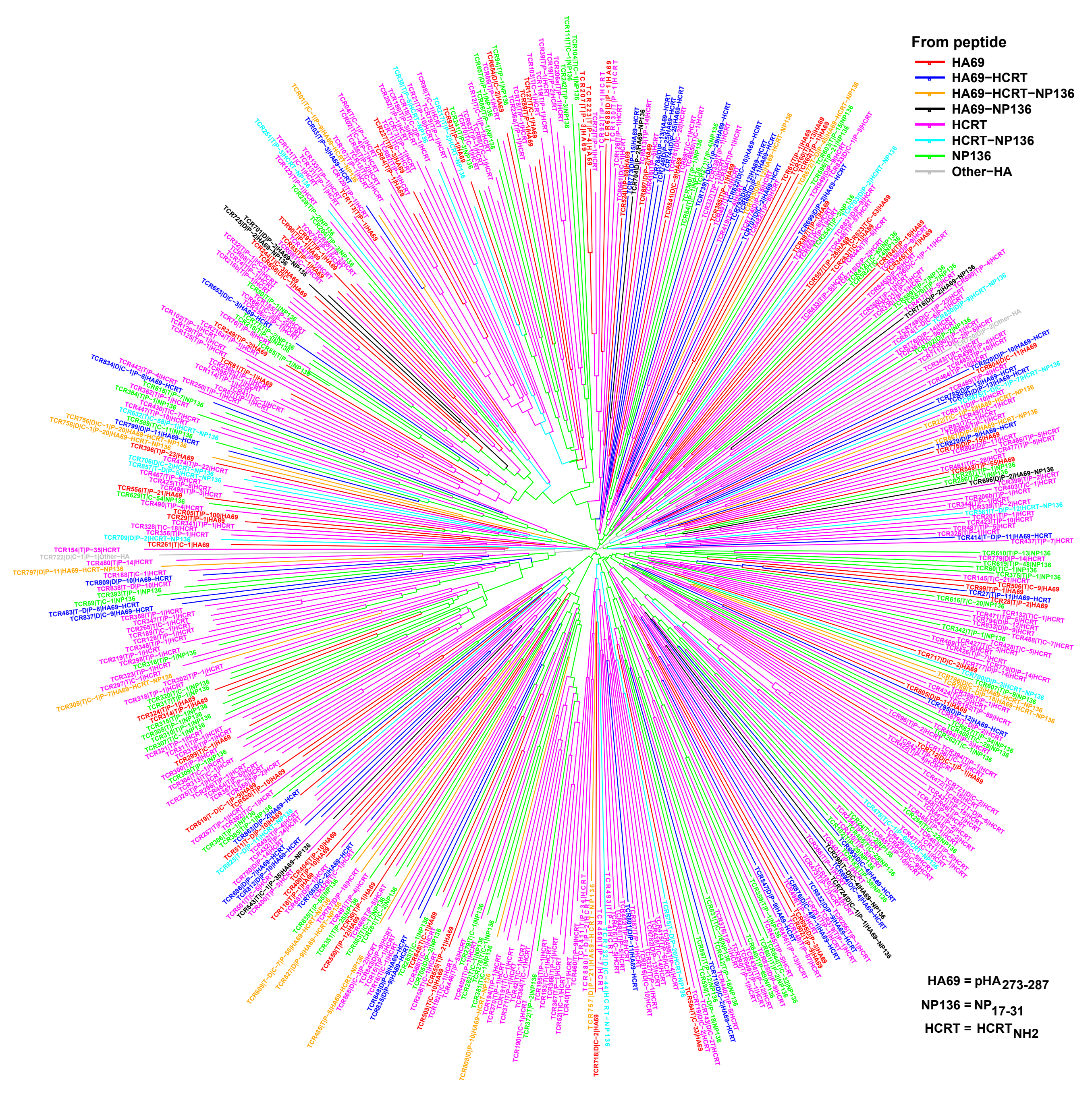
From peptide
HA69
HA69−HCRT
HA69−HCRT−NP136
HA69−NP136
HCRT
HCRT−NP136
NP136
Other−HA
HA69 = $pHA_{273-287}$
NP136 = NP_{17-31}
HCRT = $HCRT_{NH2}$

Fig. S7. Phylogenetic tree of negative TCRs. The distance between paired CDR3αβ sequences was calculated using the BLSOUM62 substitution matrix. Branch and tip label color encode the peptides from which TCRs were recovered. Tip label is ordered as follows: TCR number, recovered by tetramer (T) or dextramer (D), diagnosis of subject followed by clone count and peptides. For additional details, refer to Dataset S6.

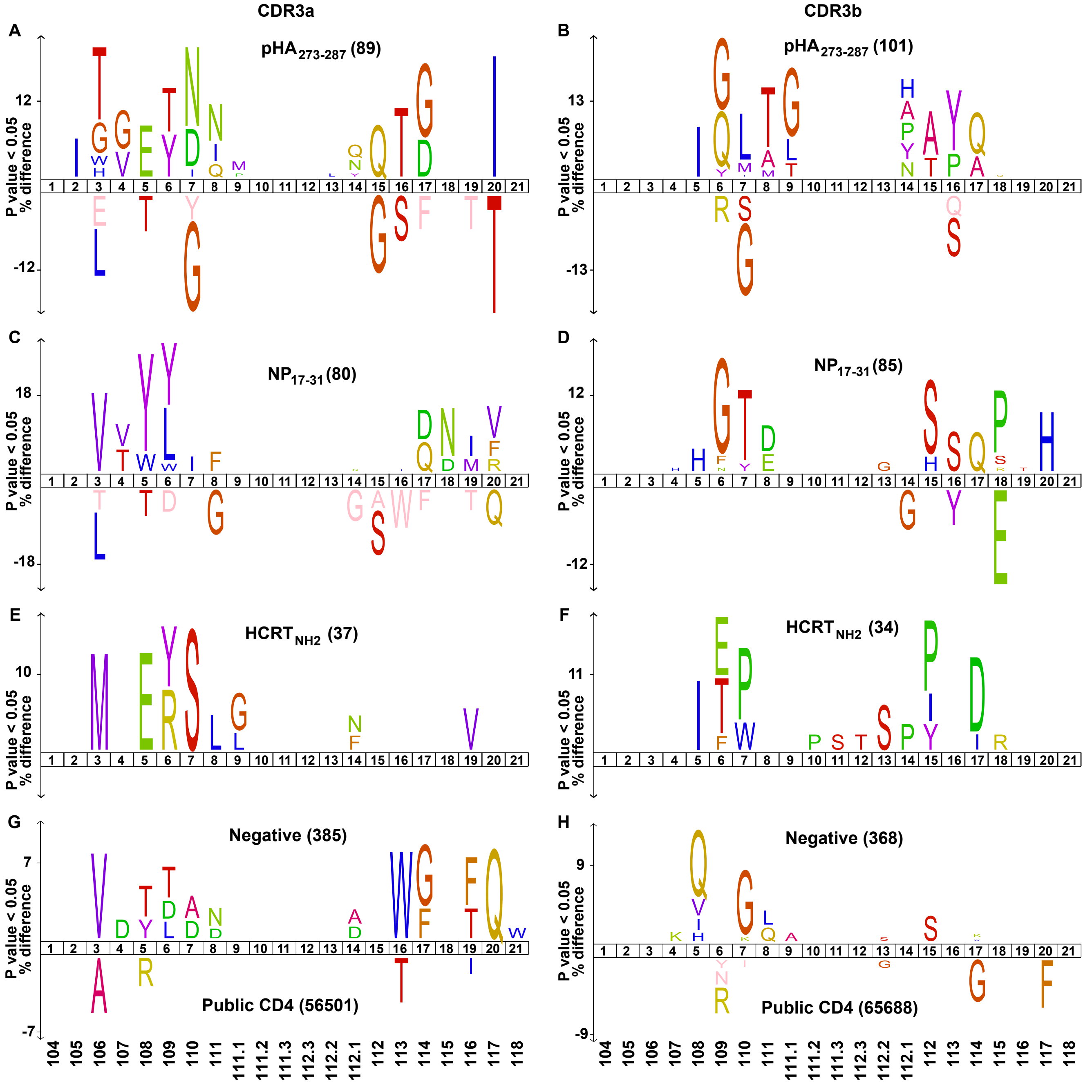
CDR3a
CDR3b
A
pHA273-287 (89)
B
pHA273-287 (101)
C
NP17-31 (80)
D
NP17-31 (85)
E
HCRTNH2 (37)
F
HCRTNH2 (34)
G
Negative (385)
Public CD4 (56501)
H
Negative (368)
Public CD4 (65688)
P value < 0.05
% difference
104 105 106 107 108 109 110 111 111.1 111.2 111.3 112.3 112.2 112.1 112 113 114 115 116 117 118

Fig. S8. Differential usage of aminoacids in activated and negative TCRs. Unique CDR3α (A, C, E) and CDR3β (B, D, F) activated by $pHA_{273-287}$, NP_{17-31} and $HCRT_{NH2}$ were compared with negative CDR3α and CDR3β, respectively. Negative CDR3α (G) and CDR3β (H) were compared with public CD4 TCR reference set (CDR3α, n = 56,501; CDR3β, n = 65,688) (see methods). Each aminoacid was assigned a position using unique numbering of TCR v-region and gaps were inserted in shorter sequences. Only aminoacids with significant different usage frequency ($p < 0.05$) are shown and visualized using Icelogo. Positively associated aminoacids at each relative position are shown above x-axis and negatively associated aminoacids are shown below. Aminoacid height is proportional to prevalence at that position. The counts of unique CDR3 sequences are shown between brackets.

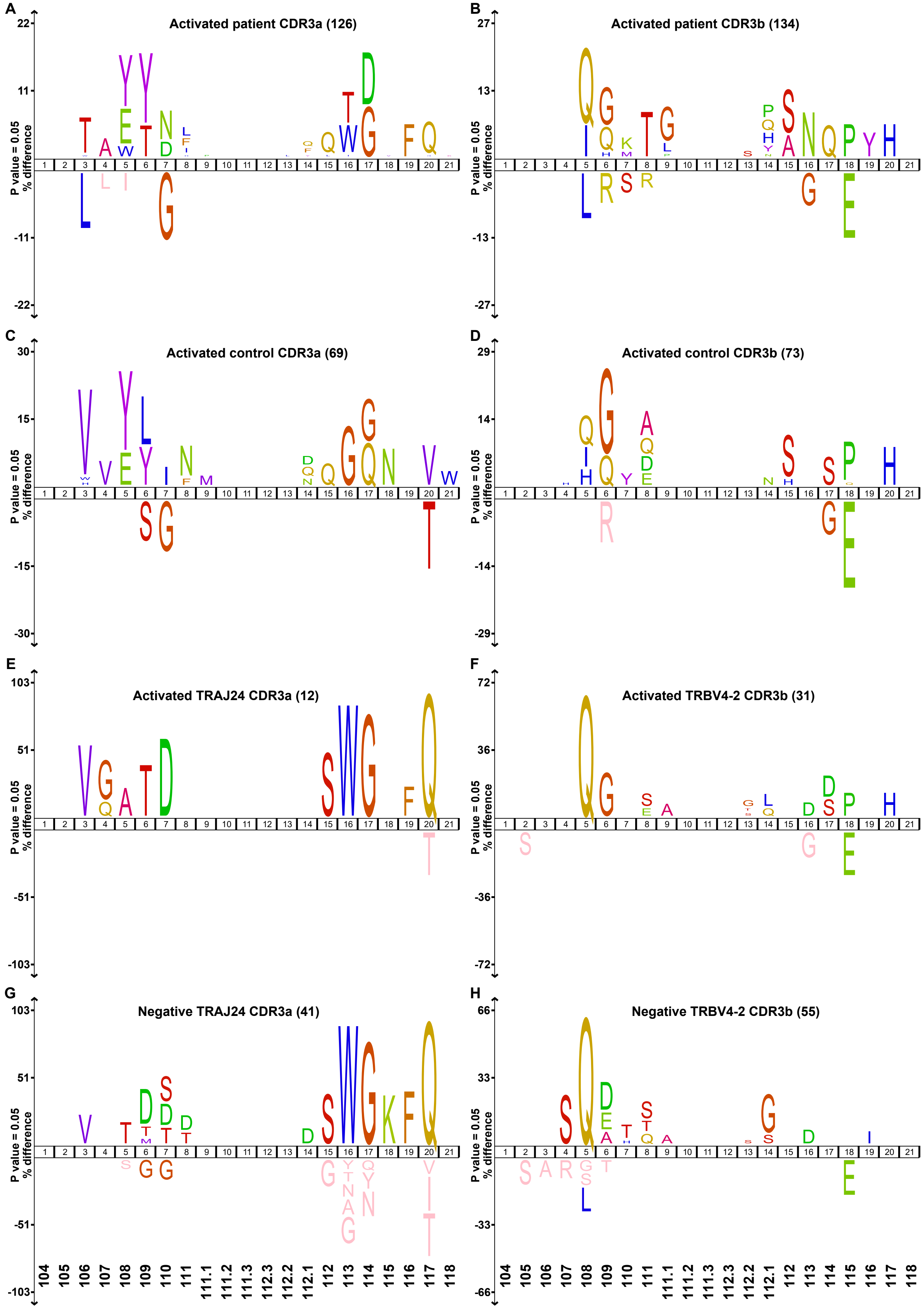
A
Activated patient CDR3a (126)
B
Activated patient CDR3b (134)
C
Activated control CDR3a (69)
D
Activated control CDR3b (73)
E
Activated TRAJ24 CDR3a (12)
F
Activated TRBV4-2 CDR3b (31)
G
Negative TRAJ24 CDR3a (41)
H
Negative TRBV4-2 CDR3b (55)
P value = 0.05
% difference
104 105 106 107 108 109 110 111 111.1 111.2 111.3 112.3 112.2 112.1 112 113 114 115 116 117 118

Fig. S9. Differential usage of aminoacids in activated NT1 case and control TCRs. Unique activated case (A and B) and control (C and D) CDR3α/β, activated and negative TRAJ24 CDR3α (E and G) and TRBV4-2 CDR3β (F and H) were compared with public CD4 TCR reference set (CDR3α, n = 56,501; CDR3β, n = 65,688) (see methods). Each aminoacid was assigned a position using unique numbering of TCR v-region and gaps were inserted in shorter sequences. Only aminoacids with significant different usage frequency ($p < 0.05$) are shown and visualized using Icelogo. Positively associated aminoacids at each relative position are shown above x-axis and negatively associated aminoacids are shown below. Aminoacid height is proportional to prevalence at that position. The counts of unique CDR3 sequences are shown between brackets.

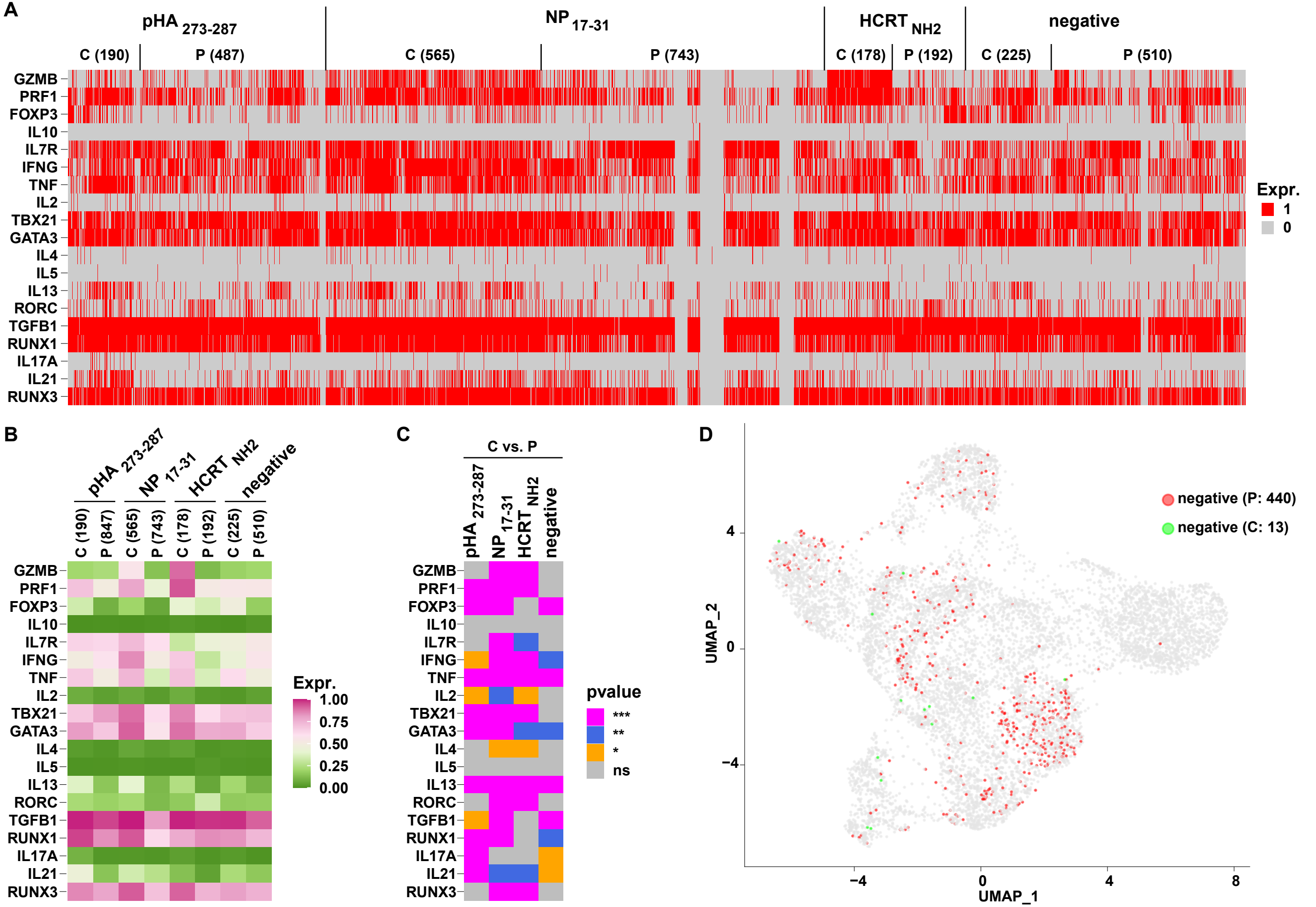

A
pHA 273-287
NP 17-31
HCRT NH2
negative
C (190)
P (487)
C (565)
P (743)
C (178)
P (192)
C (225)
P (510)
GZMB
PRF1
FOXP3
IL10
IL7R
IFNG
TNF
IL2
TBX21
GATA3
IL4
IL5
IL13
RORC
TGFB1
RUNX1
IL17A
IL21
RUNX3
Expr.
1
0
B
C (190)
P (847)
C (565)
P (743)
C (178)
P (192)
C (225)
P (510)
Expr.
1.00
0.75
0.50
0.25
0.00
C
C vs. P
pvalue
***
**
*
ns
D
negative (P: 440)
negative (C: 13)
UMAP_1
UMAP_2
−4
0
4
8

Fig. S10. Phenotypic features of antigen specific single cells. (A) Gene expression of tetramer+CD4+ cells expressing tested TCR clones (see Dataset S6). Each gene expression was assigned to 0 (reads is zero in Dataset S3) or 1 (reads is non-zero in Dataset S3) to indicate absence or presence of transcript, regardless actual reads. Cells were grouped according to their bearing TCRs activated by $pHA_{273-287}$, NP_{17-31}, $HCRT_{NH2}$ or non activation in NT1 patients and controls. Number of cells for each group is shown between brackets. (B) The mean expression of each group displayed in A. (C) P value of Wilcoxon rank sum test between patients and controls. (D) Dextramer+CD4+ cells expressing negative TCRs in the UMAP of antigen restricted CD4+ T cells of $pHA_{273-287}$, NP_{17-31} and $HCRT_{NH2}$ using dCODE® dextramer DQ0602. Numbers of patient and control cells are shown between brackets. C, control. P, patient. Expr., expression. *, p <0.05. **, p <0.01. ***, p <0.001. ns, not significant.

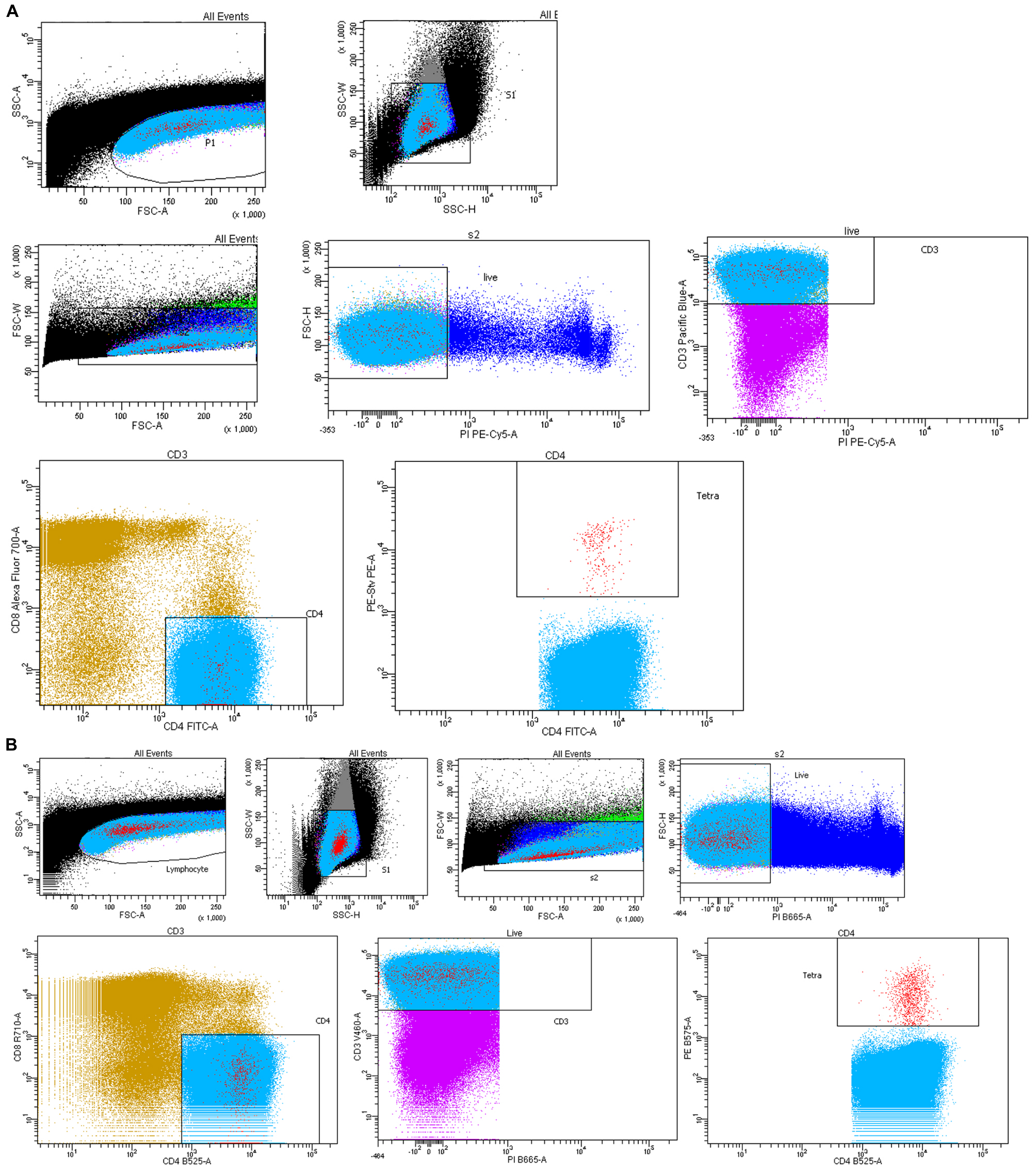
A
All Events
SSC-A
FSC-A
(x 1,000)
P1
SSC-W
SSC-H
S1
FSC-W
s2
FSC-H
live
PI PE-Cy5-A
CD3 Pacific Blue-A
CD3
CD8 Alexa Fluor 700-A
CD4 FITC-A
CD4
PE-Stv PE-A
Tetra
B
All Events
Lymphocyte
SSC-A
FSC-A
SSC-W
SSC-H
S1
FSC-W
s2
FSC-H
Live
PI B665-A
CD3
CD8 R710-A
CD4 B525-A
CD4
CD3 V460-A
PE B575-A
Tetra

Fig. S11. Gate strategy used for sorting DQ0602 tetramer (A) and dextramer (B) positive CD4+ T cells. Lymphocytes, single cells, viable cells, CD3+, CD4+CD8- and tetramer/dextramer+ T cell were selected in order, according to forward scatter (FSC) and side scatter (SSC), propidium iodide (PI) and fluorescence conjugated to antibody. Cells were gated for sorting in the last panel.

SI references

1. Luo G, *et al.* (2018) Autoimmunity to hypocretin and molecular mimicry to flu in type 1 narcolepsy. *Proceedings of the National Academy of Sciences* 115(52):E12323-E12332.
2. Kwok WW, *et al.* (2000) HLA-DQ Tetramers Identify Epitope-Specific T Cells in Peripheral Blood of Herpes Simplex Virus Type 2-Infected Individuals: Direct Detection of Immunodominant Antigen-Responsive Cells. *The Journal of Immunology* 164(8):4244-4249.
3. Day CL, *et al.* (2003) Ex vivo analysis of human memory CD4 T cells specific for hepatitis C virus using MHC class II tetramers. *Journal of Clinical Investigation* 112(6):831-842.
4. Luo G, Yogeshwar S, Lin L, & Mignot EJ (2021) T cell reactivity to regulatory factor X4 in type 1 narcolepsy. *Sci Rep* 11(1):7841.
5. Reijonen H & Kwok WW (2003) Use of HLA class II tetramers in tracking antigen-specific T cells and mapping T-cell epitopes. *Methods* 29(3):282-288.
6. Han A, Glanville J, Hansmann L, & Davis MM (2014) Linking T-cell receptor sequence to functional phenotype at the single-cell level. *Nature biotechnology* 32(7):684-692.
7. Glanville J, *et al.* (2017) Identifying specificity groups in the T cell receptor repertoire. *Nature* 547(7661):94-98.
8. Anonymous (2020) R Core Team (2020). *R: A language and environment for statistical computing. R Foundation for Statistical Computing, Vienna, Austria.*
9. Kang HM, *et al.* (2018) Multiplexed droplet single-cell RNA-sequencing using natural genetic variation. *Nature Biotechnology* 36(1):89-94.
10. Cano-Gamez E, *et al.* (2020) Single-cell transcriptomics identifies an effectorness gradient shaping the response of CD4(+) T cells to cytokines. *Nat Commun* 11(1):1801.
11. Hao Y, *et al.* (2021) Integrated analysis of multimodal single-cell data. *Cell* 184(13):3573-3587.e3529.
12. Butler A, Hoffman P, Smibert P, Papalexi E, & Satija R (2018) Integrating single-cell transcriptomic data across different conditions, technologies, and species. *Nature Biotechnology* 36(5):411-420.
13. Satija R, Farrell JA, Gennert D, Schier AF, & Regev A (2015) Spatial reconstruction of single-cell gene expression data. *Nature Biotechnology* 33(5):495-502.
14. Stuart T, *et al.* (2019) Comprehensive Integration of Single-Cell Data. *Cell* 177(7):1888-1902.e1821.
15. Kowalczyk MS, *et al.* (2015) Single-cell RNA-seq reveals changes in cell cycle and differentiation programs upon aging of hematopoietic stem cells. *Genome Res* 25(12):1860-1872.

16. McKinney DM, *et al.* (2013) A strategy to determine HLA class II restriction broadly covering the DR, DP, and DQ allelic variants most commonly expressed in the general population. *Immunogenetics* 65(5):357-370.
17. Bodenhofer U, Bonatesta E, Horejš-Kainrath C, & Hochreiter S (2015) msa: an R package for multiple sequence alignment. *Bioinformatics* 31(24):3997-3999.
18. Csardi GN, Tamas (2006) The igraph software package for complex network research. *InterJournal, Complex Systems* 1695(5):1--9.
19. Wagih O (2017) ggseqlogo: a versatile R package for drawing sequence logos. *Bioinformatics* 33(22):3645-3647.
20. Colaert N, Helsens K, Martens L, Vandekerckhove J, & Gevaert K (2009) Improved visualization of protein consensus sequences by iceLogo. *Nat Methods* 6(11):786-787.
21. Tran E, *et al.* (2015) Immunogenicity of somatic mutations in human gastrointestinal cancers. *Science* 350(6266):1387-1390.
22. Zacharakis N, *et al.* (2018) Immune recognition of somatic mutations leading to complete durable regression in metastatic breast cancer. *Nat Med* 24(6):724-730.
23. Leko V, *et al.* (2019) Identification of Neoantigen-Reactive Tumor-Infiltrating Lymphocytes in Primary Bladder Cancer. *J Immunol* 202(12):3458-3467.
24. Tran E, *et al.* (2014) Cancer immunotherapy based on mutation-specific CD4+ T cells in a patient with epithelial cancer. *Science* 344(6184):641-645.
25. Petersen J, *et al.* (2014) T-cell receptor recognition of HLA-DQ2-gliadin complexes associated with celiac disease. *Nat Struct Mol Biol* 21(5):480-488.
26. Veatch JR, *et al.* (2018) Tumor-infiltrating BRAFV600E-specific CD4+ T cells correlated with complete clinical response in melanoma. *J Clin Invest* 128(4):1563-1568.
27. Deng L, *et al.* (2007) Structural basis for the recognition of mutant self by a tumor-specific, MHC class II-restricted T cell receptor. *Nat Immunol* 8(4):398-408.
28. Beringer DX, *et al.* (2015) T cell receptor reversed polarity recognition of a self-antigen major histocompatibility complex. *Nat Immunol* 16(11):1153-1161.
29. Malekzadeh P, *et al.* (2019) Neoantigen screening identifies broad TP53 mutant immunogenicity in patients with epithelial cancers. *J Clin Invest* 129(3):1109-1114.
30. Veatch JR, *et al.* (2019) Endogenous CD4(+) T Cells Recognize Neoantigens in Lung Cancer Patients, Including Recurrent Oncogenic KRAS and ERBB2 (Her2) Driver Mutations. *Cancer immunology research* 7(6):910-922.
31. Rödström KE, Elbing K, & Lindkvist-Petersson K (2014) Structure of the superantigen staphylococcal enterotoxin B in complex with TCR and peptide-MHC demonstrates absence of TCR-peptide contacts. *J Immunol* 193(4):1998-2004.
32. Poncette L, Chen X, Lorenz FK, & Blankenstein T (2019) Effective NY-ESO-1-specific MHC II-restricted T cell receptors from antigen-negative hosts enhance tumor regression. *J Clin Invest* 129(1):324-335.
33. Ueda N, *et al.* (2018) BCR-ABL-specific CD4(+) T-helper cells promote the priming of antigen-specific cytotoxic T cells via dendritic cells. *Cell Mol Immunol* 15(1):15-26.
34. Hennecke J, Carfi A, & Wiley DC (2000) Structure of a covalently stabilized complex of a human alphabeta T-cell receptor, influenza HA peptide and MHC class II molecule, HLA-DR1. *The EMBO journal* 19(21):5611-5624.

35. Hennecke J & Wiley DC (2002) Structure of a complex of the human alpha/beta T cell receptor (TCR) HA1.7, influenza hemagglutinin peptide, and major histocompatibility complex class II molecule, HLA-DR4 (DRA*0101 and DRB1*0401): insight into TCR cross-restriction and alloreactivity. *J Exp Med* 195(5):571-581.
36. Li Y*, et al.* (2005) Structure of a human autoimmune TCR bound to a myelin basic protein self-peptide and a multiple sclerosis-associated MHC class II molecule. *The EMBO journal* 24(17):2968-2979.
37. Petersen J*, et al.* (2015) Determinants of gliadin-specific T cell selection in celiac disease. *J Immunol* 194(12):6112-6122.
38. Broughton SE*, et al.* (2012) Biased T cell receptor usage directed against human leukocyte antigen DQ8-restricted gliadin peptides is associated with celiac disease. *Immunity* 37(4):611-621.
39. Huang H, Wang C, Rubelt F, Scriba TJ, & Davis MM (2020) Analyzing the Mycobacterium tuberculosis immune response by T-cell receptor clustering with GLIPH2 and genome-wide antigen screening. *Nature Biotechnology* 38(10):1194-1202.

Datasets

Dataset S1. Peptide used in this research

Dataset S2. Frequency of antigen specific CD4+ T cells in each subject

Dataset S3. TCR from tetramer sorted single cell sequenced in 96-well plate

Dataset S4. TCR from dCODE® dextramer and 10x genomics

Dataset S5. TCR enrichment of tetramer and dextramer

Dataset S6. TCR tested

Dataset S7. Summary of TCR activation and retrieved by peptide

Dataset S8. Statistics of TCR activation according to hamming distance

Dataset S9. GLIPH motif of tested TCR

Dataset S10. Frequency versus age, diagnosis, vaccination and gender

Dataset S11. Frequency of Pandemrix vaccination versus non vaccination
